# High Fat Activates O-GlcNAcylation and Affects AMPK/ACC Pathway to Regulate Lipid Metabolism

**DOI:** 10.3390/nu13061740

**Published:** 2021-05-21

**Authors:** Yuning Pang, Xiang Xu, Xiaojun Xiang, Yongnan Li, Zengqi Zhao, Jiamin Li, Shengnan Gao, Qiangde Liu, Kangsen Mai, Qinghui Ai

**Affiliations:** 1 The Key Laboratory of Aquaculture Nutrition and Feed (Ministry of Agriculture and Rural Affairs), The Key Laboratory of Mariculture (Ministry of Education), Ocean University of China, 5 Yushan Road, Qingdao 266003, China; pyn2270@stu.ouc.edu.cn (Y.P.); xuxiang@stu.ouc.edu.cn (X.X.); xiangxiaojun@stu.ouc.edu.cn (X.X.); lyn@qdio.ac.cn (Y.L.); zhaozengqi@stu.ouc.edu.cn (Z.Z.); ljm5697@ouc.edu.cn (J.L.); gaoshengnan@stu.ouc.edu.cn (S.G.); lqd7230@stu.ouc.edu.cn (Q.L.); kmai@ouc.edu.cn (K.M.); 2 Laboratory for Marine Fisheries Science and Food Production Processes, Qingdao National Laboratory for Marine Science and Technology, 1 Wenhai Road, Qingdao 266237, China

**Keywords:** large yellow croaker, acetyl-CoA carboxylase, O-GlcNAc, AMPK, lipid metabolism

## Abstract

A high-fat diet often leads to excessive fat deposition and adversely affects the organism. However, the mechanism of liver fat deposition induced by high fat is still unclear. Therefore, this study aimed at acetyl-CoA carboxylase (ACC) to explore the mechanism of excessive liver deposition induced by high fat. In the present study, the ORF of ACC1 and ACC2 were cloned and characterized. Meanwhile, the mRNA and protein of ACC1 and ACC2 were increased in liver fed with a high-fat diet (HFD) or in hepatocytes incubated with oleic acid (OA). The phosphorylation of ACC was also decreased in hepatocytes incubated with OA. Moreover, AICAR dramatically improved the phosphorylation of ACC, and OA significantly inhibited the phosphorylation of the AMPK/ACC pathway. Further experiments showed that OA increased global O-GlcNAcylation and agonist of O-GlcNAcylation significantly inhibited the phosphorylation of AMPK and ACC. Importantly, the disorder of lipid metabolism caused by HFD or OA could be rescued by treating CP-640186, the dual inhibitor of ACC1 and ACC2. These observations suggested that high fat may activate O-GlcNAcylation and affect the AMPK/ACC pathway to regulate lipid synthesis, and also emphasized the importance of the role of ACC in lipid homeostasis.

## 1. Introduction

Excessive liver fat deposition caused by a high-fat diet (HFD) has gradually become a threat to organism health. The regulatory mechanisms of fat deposition induced by high fat has attracted research attention. Acetyl-CoA carboxylase (ACC) is the first rate-limiting enzyme in the de novo lipogenesis (DNL) process, which plays an important role in fatty acid synthesis and fatty acid oxidation (FAO). The inhibition or knockout of ACC1 and ACC2 has a positive effect for alleviating liver fat deposition, which promotes ACC to be an attractive target for metabolic diseases in mammals over the past few decades [1,2,3,4,5,6,7,8].

ACC catalyzes the conversion of acetyl-CoA to malonyl-CoA [9]. There are two isoforms of ACC in mammals: ACC1produced malonyl-CoA is mainly used in fatty acid synthesis [10], while malonyl CoA produced by ACC2 could effectively regulate the mitochondrial fatty acid oxidation by inhibiting carnitine palmitoyltransferase 1 (CPT1) [11]. ACC is closely related to the nutritional status in mammals. Kinnunen. et al. found that the expression of the *acc1* gene in the liver was significantly inhibited after starvation stress [12]. The high fructose and sucrose diets increased the expression of *acc1* in rats [13]. Besides, ACC could be post-transcriptionally modified and then affect its activity. AMPK is considered to be a classical phosphorylation upstream kinase of ACC in mammals. AMPK inhibited ACC1 and ACC2 activity by phosphorylating ACC1 Ser79 and ACC2 Ser212 in rats [14]. Studies on zebrafish have shown that the anti-adipogenic effect of cholecalciferole may be related to the activation of AMPK/ACC [15]. However, whether nutritional status could affect AMPK/ACC in fish has not been thoroughly elucidated.

O-GlcNAcylation is also an important post-translational modification, which is affected by the hexosamine synthesis pathway (HBP) [16]. The pathway integrates glucose, amino acids, fatty acids and nucleotide metabolism to produce the donor substrate of O-GlcNAcylation (UDP-GlcNAC), which is involved in the regulation of multiple intracellular signaling pathways and regulates growth, proliferation, and hormonal response [17,18,19]. However, whether O-GlcNAcylation is involved in the process of liver fat deposition induced by high fat is not completely understood.

The large yellow croaker is an important economically cultured marine fish in China. Previous studies have shown that large yellow croakers fed with HFD could cause excessive fat deposition in liver [20,21]. Thus, the large yellow croaker is an appropriate model to investigate excessive fat deposition [22,23,24]. However, it is not clear whether the excessive fat deposition caused by high fat is related to ACC. In this study, we studied the effects of ACC inhibition on lipid metabolism in the large yellow croaker, and explored the regulatory mechanism of high fat to ACC. This study contributed to alleviating excessive hepatic fat deposition caused by HFD, thereby improving the growth and quality of aquatic animals, and providing a guarantee for human food safety.

## 2. Materials and Methods

### 2.1. Animal Studies and Ethics Statements

In this present study, all experiments were performed in strict accordance with the Management Rule of Laboratory Animals (Chinese Order No. 676 of the State Council, revised 1 March 2017).

Large yellow croaker juveniles were provided by Xiangshan Harbor Aquatic Seed (Ningbo, China). The preparation of experimental diets and the fish feeding procedure referred to the research of Li. et al. [21]. Fishes were fed with a control diet (45% crude protein and 13% crude fat) and HFD (45% crude protein and 18% crude fat) for 10 weeks. At the end of the feeding trial, DMSO or ACC inhibitor (25 mg/kg CP-640186) was injected intraperitoneally into the control group and HFD group, respectively. After injection, fishes were put back into the cage and fed with the control diet or HFD normally. Intestine, spleen, kidney, gill, liver, eye, heart, muscle, brain, adipose and plasma were collected at 24 h post-injection. Samples were rapidly frozen in liquid nitrogen and stored at −80 °C for subsequent analysis.

### 2.2. Primary Hepatocytes Culture and Treatment

The isolation and culture of primary hepatocytes of large yellow croakers were referred to the previously described procedures with some modification [25]. In brief, the liver tissue was separated and rinsed with phosphate buffered saline (PBS, pH 7.4, Gibco, Carlsbad, CA, USA). Thereafter, the liver was aseptically chopped into small pieces and then digested in 0.25% sterile trypsin at room temperature for 15 min. The digestion reaction was neutralized with an equal volume of complete medium. The cell suspension was collected and filtered through a sterile 75 μm filter screen, and then centrifuged at low speed (1000 rpm, 5 min) and discarded supernatant. Hepatocytes were resuspended in DMEM/F12 medium (Biological Industries, Beit-Haemek, Israel) containing 15% FBS (Biological Industries) and 100 IU/mL penicillin–streptomycin (Solarbio, Beijing, China). After cell counting, cells were resuspended with complete medium, the diluted cells were poured into a 6-well plate and cultured in a 28 °C cell incubator for subsequent experiments.

The in vitro high-fat model in this study was established with 800 μM oleic acid (OA, C18:1n-9) incubation for 24 h, while the control group was incubated with 1% bovine serum albumin (BSA, Equitech-Bio, Kerrville, TX, USA). It is considered that the modeling is successful if the triglyceride (TG) content increases more than twice. Primary hepatocytes were preincubated with 50 μM CP-640186(dual inhibitor of ACC1 and ACC2, MCE, Shanghai, China), 500 μM AICAR (AMPK agonist, MCE), 20 μM PugNAc (O-GlcNAcase inhibitor, Sigma-Aldrich, St. Louis, MI, USA) for 12 h or 24 h to collect samples.

### 2.3. Cloning and Sequence Analysis of the ACC Gene

Total RNA extraction and cDNA synthesis were performed according to the method described previously [25]. Cloning primers were designed by Primer Premier 5.0 software according to the predicted sequence (ACC1, XM_027273319.1; ACC2, XM_027277579.1) on the National Center for Biotechnology Information (https://www.ncbi.nlm.nih.gov/, accessed on 4 May 2021), and PCR amplification was performed by PrimeSTAR@Max DNA Polymerase (Takara, Tokyo, Japan). PCR products were separated by 1% agarose gel electrophoresis, and then the target fragments were purified by SanPrep Column DNA Gel (Sangon Biotech, Shanghai, China). The product fragments were connected to pEASY-T1 simple cloning plasmid (TransGen, Beijing, China) and sequenced (Sangon Biotech).

The amino acid sequence analysis was performed using DNAMAN. The phylogenetic tree was performed using MEGA 7.0 through the neighbor joining method.

### 2.4. Subcellular Localization

Human embryonic kidney (HEK) 293T cells were cultured in Dulbecco’s modified Eagle’s medium (DMEM) containing 10% fetal bovine serum (FBS, Biological Industries) and 100 IU/mL penicillin–streptomycin (Solarbio) at 37 °C, 5% CO_2_ cell incubator. Specific procedures of culture and subculture were performed as described previously [26].

The open reading frame of croaker ACC1 and ACC2 with the deletion of stop codons were constructed into vector pcDNA 3.1-EGFP, respectively. Fusion plasmids were transfected into HEK293T cells with EZ Trans Cell transfection (Life Ilab, Shanghai, China). Cells were fixed in 4% paraformaldehyde after 24 h. The cell membrane, nucleus and mitochondria were stained with Dil (Beyotime, Shanghai, China), DAPI (Beyotime) and MitoLite (AAT Bioquest, Sunnyvale, CA, USA), respectively, and then visualized by laser confocal microscopy (Leica, Carl Zeiss, Jena, Germany).

### 2.5. TG Contents Determination

TG contents were determined by commercial kits (Applygen Technologies, Beijing, China) based on manufacturer’s instructions.

### 2.6. Relative mRNA Quantification

Real-time quantitative-polymerase chain reaction (RT-qPCR) was used to determine gene expression and was carried out by quantitative thermal cycler (BIO- RAD, Hercules, CA, USA) using the SYBR Green real-time PCR kit (Vazyme, Nanjing, China). Primers were designed by Primer Premier 5.0 (Table S1). *β-actin* was selected as the internal control. During the experiment, the melt curve analysis of each gene was analyzed to confirm that there was only one single peak in the thermal decomposition. Gene expression was analyzed using the 2^−ΔΔCt^ method [27].

### 2.7. Western Blot Analysis

Western blot assay was carried out referring to a previous publication [24]. Antibodies, including AMPKα (cat. no.2532), phosphor-AMPKα (Thr172) (cat. no. 2531), ACC (cat. no.3662), phosphor-ACC (Ser79) (cat. no.3661), and O-GlcNAc (cat. no.9875), were purchased from Cell Signaling Technology (Danvers, MA, USA). Anti-glyceraldehyde 3-phosphate dehydrogenase (GAPDH) antibodies (cat. no. 309154) and HRP-conjugated secondary antibodies were purchased from Golden Bridge Biotechnology (Beijing, China).

### 2.8. Statistical Analysis

All experimental results were analyzed by SPSS 22.0, and values were presented as means ± SEM. The data of two groups were analyzed by an independent sample *t*-test. The data of three groups and above were analyzed by one-way ANOVA, and then multiple comparisons were made by Tukey’s multiple range test. *p* < 0.05 was set as statistically significant.

## 3. Results

### 3.1. Molecular Characterization of Large Yellow Croaker ACC1 and ACC2

**Figure 1:**
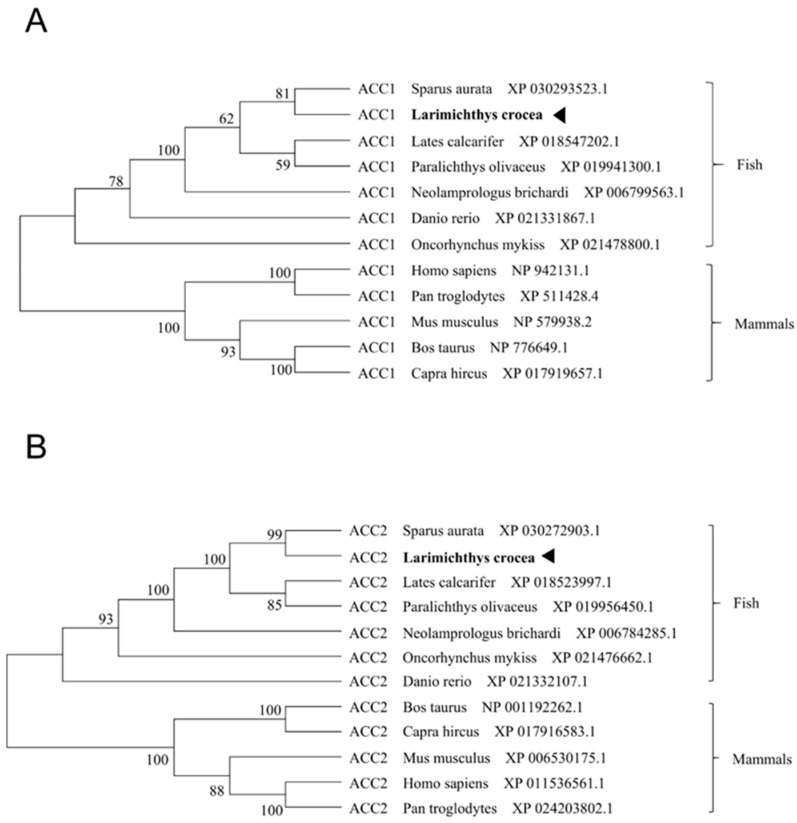
The phylogenetic tree of ACC1 (**A**) and ACC2 (**B**) from large yellow croaker and other species. The phylogenetic tree was constructed by the neighbor joining method based on amino acid sequences using MEGA 7.0. The numbers on the node were the percentage obtained by bootstrap after 1000 times iterations.

**Figure 2:**
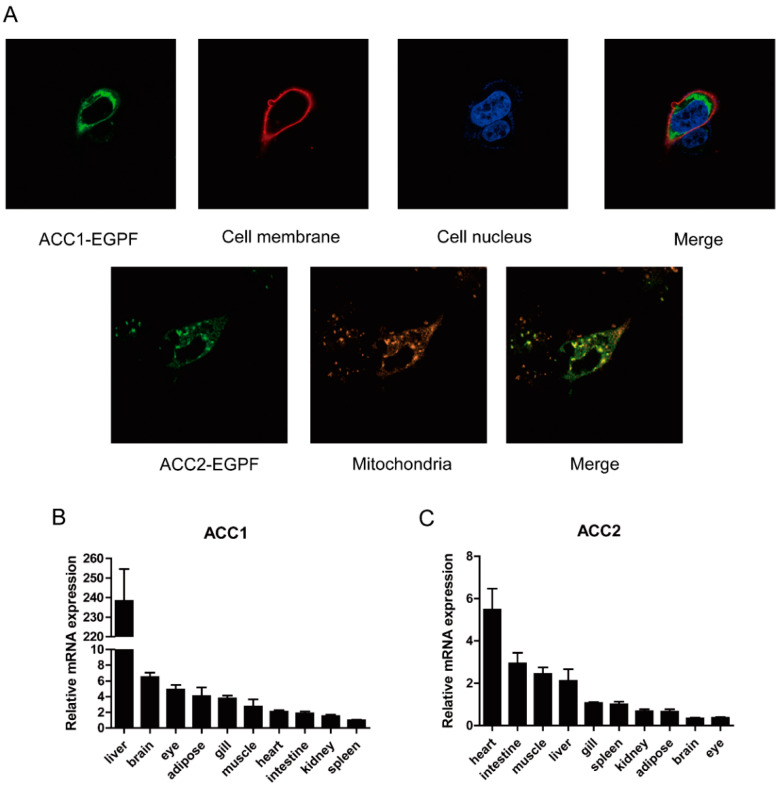
Subcellular localization and tissue distribution of large yellow croaker ACC1 and ACC2. (**A**) Subcellular localization of ACC1 and ACC2. ACC1-EGFP and ACC2-EGFP were transfected into the HEK293T cells. Cell membrane, nucleus and mitochondria were stained with Dil, DAPI and MitoLite. Confocal laser microscopy was used to visualize. (**B**,**C**) Tissue distribution of ACC1 and ACC2. The expression of *acc1* in the spleen was selected as control, the expression of *acc2* in the eye was selected as control. Results were shown as mean ± S.E.M in the figure (*n* = 3).

Large yellow croaker *acc1* contained an ORF of 7194 bp encoding a putative protein of 2397 amino acid (AA) (Figure S1). Large yellow croaker *acc2* contained an ORF of 7293 bp encoding a putative protein of 2430 AA (Figure S2). Multiple sequence alignments showed that the AA sequences of large yellow croaker ACC1 and ACC2 shared high identity with other species (ACC1 89%, ACC2 81%) (Figures S3 and S4). The phylogenetic tree analysis showed that ACC1 was clustered with teleost ACC1, nested Sparus aurata ACC1, and ACC2 was clustered with teleost ACC2, nested with Sparus aurata ACC2 (Figure 1A,B). Subcellular localization of large yellow croaker ACC1 and ACC2 were observed by transfecting fusion ACC1-EGFP protein and ACC2-EGFP protein into HEK293T cells. Results showed that croaker ACC1 was widely expressed in the cytoplasm rather than in the nucleus, and ACC2 was primarily located in mitochondria (Figure 2A).

The results of tissue distribution showed that mRNA levels of *acc1* and *acc2* were widely expressed in the various tissues of the large yellow croaker. The highest expression of *acc1* was observed in the liver while the lowest was detected in the spleen (Figure 2B). The highest expression of *acc2* was in the heart, followed by the intestine, muscle and liver, and the lowest in the eyes (Figure 2C).

### 3.2. High Fat Affects the Expression and Phosphorylation of ACC

**Figure 3:**
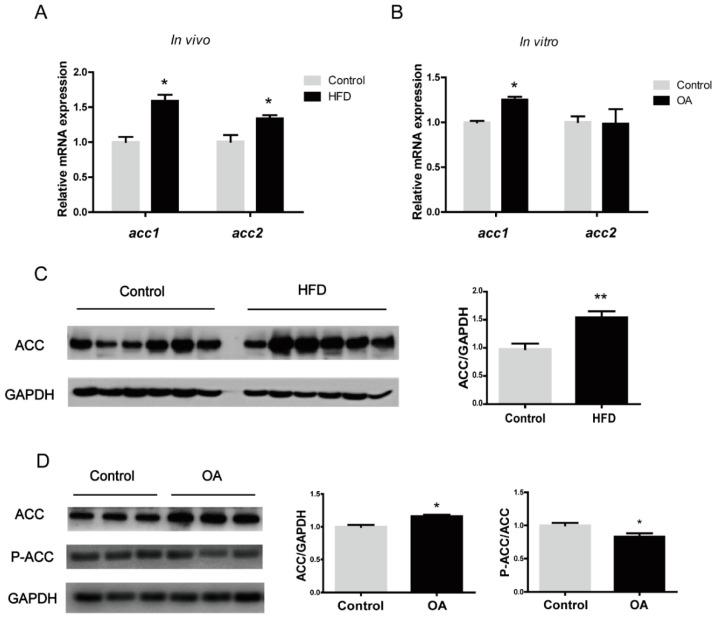
The response of ACC1 and ACC2 to high-fat diet (in vivo) and oleic acid (in vitro) of large yellow croaker. (**A**) mRNA levels of *acc1* and *acc2* in the liver of large yellow croaker fed with high-fat diet and control diet. (**B**) mRNA levels of *acc1* and *acc2* in primary hepatocytes of large yellow croaker incubated with 800 μM oleic acid (OA) or 1% BSA for 24 h. (**C**) Western blots of ACC in liver of large yellow croaker fed with high-fat diet and control diet. (**D**) Western blots of ACC in primary hepatocytes of large yellow croaker incubated with 800 μM oleic acid (OA) or 1% BSA for 24 h. The GAPDH was selected as the reference protein. Results were shown as mean ± S.E.M and analyzed by *t*-test. The same letter represented no significant difference (*p* > 0.05). * *p* < 0.05, ** *p* < 0.01.

In order to explore whether high-fat-induced fat deposition is related to ACC, we examined mRNA and protein expression of ACC in vivo and in vitro under high-fat conditions. HFD significantly increased the expression of *acc1* and *acc2* mRNA in the liver of large yellow croakers (Figure 3A) (*p* < 0.05). In addition, primary hepatocytes were incubated with 800 μM OA or 1% BSA for 24 h. The expression of *acc1* was increased significantly (*p* < 0.05), and *acc2* mRNA levels were not significantly different after OA treatment. (Figure 3B). Correspondingly, OA treatment significantly increased the protein level of ACC (Figure 3C,D).

### 3.3. Activation of AMPK/ACC Pathway Alleviated Excessive Fat Deposition Induced by OA

**Figure 4:**
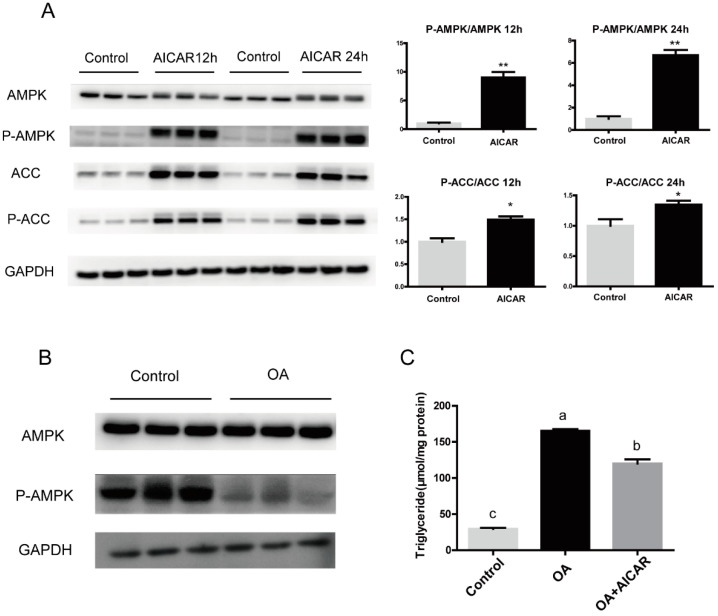
The regulation of AMPK/ACC pathway in primary hepatocytes of large yellow croaker incubated with oleic acid. (**A**) Western blots of total and phospho-proteins in primary hepatocytes of large yellow croaker incubated with 500 μM AICAR for 12 h or 24 h. (**B**) Western blots of total and phospho-proteins in primary hepatocytes of large yellow croaker incubated with 800 μM oleic acid (OA) or 1% BSA for 24 h. (**C**) Levels of triglyceride treated with 500 μM AICAR primary hepatocytes of large yellow croaker. The GAPDH was selected as the reference protein. Results were shown as mean ± S.E.M and analyzed by one-way ANOVA followed by Tukey’s multiple range test (*n* = 3). The same letter represented no significant difference (*p* > 0.05). * *p* < 0.05, ** *p* < 0.01.

In order to explore the regulatory mechanism of high fat to ACC, AICAR, an AMPK activator, was used to treat primary hepatocytes of large yellow croakers. The results indicated that AICAR significantly increased ACC phosphorylation (Figure 4A). Incubating primary hepatocytes with 800 μM OA decreased AMPK and ACC phosphorylation to a certain extent (Figure 4B). To test whether AMPK might be a target of OA induced fat deposition, we used OA and AICAR co-incubated primary hepatocytes and examined TG content. AICAR suppressed the OA-induced increasement of TG content (*p* < 0.05) (Figure 4C), suggesting the AMPK/ACC pathway effectively influenced OA-induced excessive fat deposition.

### 3.4. OA Regulated AMPK/ACC Pathway by Activating Global O-GlcNAcylation

**Figure 5:**
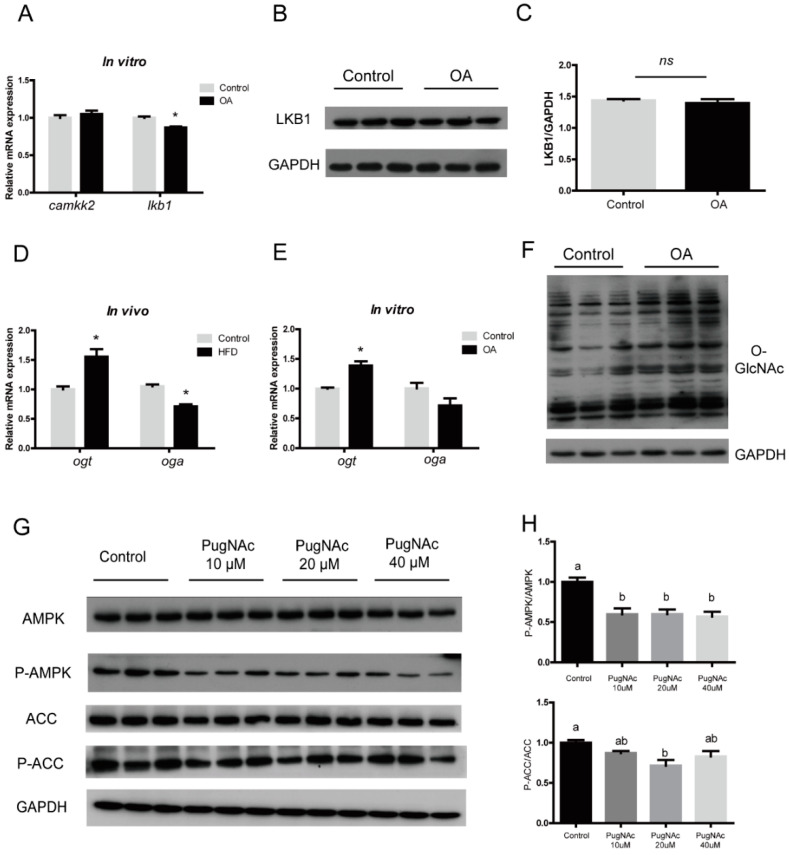
Oleic acid regulated AMPK/ACC pathway by activating global O-GlcNAc. (**A**) mRNA levels of *camkk2* and *lkb1* in the liver of large yellow croaker fed with high-fat diet and control diet. (**B**,**C**) Western blots of LKB1 in primary hepatocytes of large yellow croaker incubated with 800 μM oleic acid (OA) or 1% BSA for 24 h. (**D**) mRNA levels of *ogt* and *oga* in the liver of large yellow croaker fed with high-fat diet and control diet. (**E**) mRNA levels of *ogt* and *oga* in primary hepatocytes of large yellow croaker incubated with 800 μM oleic acid (OA) or 1% BSA for 24 h. (**F**) Western blots of O-GlcNAc in primary hepatocytes of large yellow croaker incubated with 800 μM oleic acid (OA) or 1% BSA for 24 h. (**G**,**H**) Western blots of total and phospho-AMPK and phospho-ACC in primary hepatocytes of large yellow croaker treated with 10 μM, 20 μM and 40 μM PugNAc. The GAPDH was selected as the reference protein. All results were shown as mean ± S.E.M and analyzed by Tukey’s multiple range test (*n* = 3). The same letter represented no significant difference (*p* > 0.05). ns represented no significant difference. * *p* < 0.05.

Given the pivotal role of AMPK in regulating lipid metabolism, we tried to find the upstream molecule of AMPK. The mRNA expression of liver kinase 1 (*lkb1*) decreased significantly in the HFD group (*p* < 0.05), while calcium/calmodulin-dependent protein kinase kinase 2 (*camkk2*) did not change significantly (Figure 5A). However, the protein level of LKB1 were not significantly different among treatments (Figure 5B,C), indicating that there are other stronger regulation modes in the upstream of AMPK. Our results revealed that O-GlcNAc transferase (ogt) and O-GlcNAcase (oga), the only pair of enzymes controlling O-GlcNAcylation in the organism, increased (*p* < 0.05) and decreased (*p* < 0.05) significantly in the HFD group in mRNA levels, respectively. In vitro, the mRNA level of *ogt* was significantly increased, while *oga* was decreased to some extent without significant difference (Figure 5D,E). Meanwhile, Western blot results showed that the O-GlcNAcylation also increased significantly after 24 h of OA incubation in hepatocytes (Figure 5F). When PugNAc was used to increase the level of O-GlcNAcylation in primary hepatocytes of large yellow croakers, the phosphorylation of AMPK and ACC was significantly inhibited (Figure 5G,H). In summary, these data suggested that OA may activate the O-GlcNAcylation level and inhibit the phosphorylation of the AMPK/ACC pathway.

### 3.5. Inhibition of ACC Activity Regulated Lipid Homeostasis

**Figure 6:**
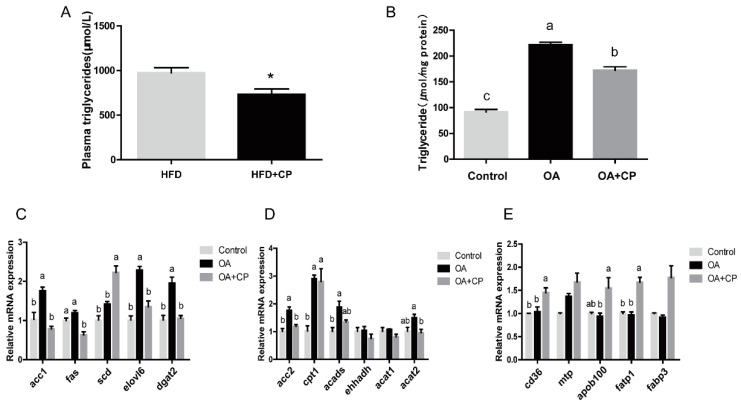
Effects of ACC inhibition on lipid metabolism of large yellow croakers. (**A**) Levels of plasma triglyceride in large yellow croaker fed with high-fat diet after injecting with 25 mg/kg CP-640186(CP) (*n* = 6). (**B**) Levels of triglyceride in primary hepatocytes of large yellow croaker after treated with 50 μM CP-640186 and 800 μM oleic acid (OA). (**C**–**E**) mRNA levels of lipogenesis genes *(acc1, fas, scd, elovl6, dgat2*), fatty acid oxidation genes (*acc2, cpt1, acads, ehhadh, acat1, acat2*) and lipid transport genes (*cd36, mtp, apob100, fatp1.fabp3*) were treated with 800 μM OA and 50 μM CP-640186. Results were shown as mean ± S.E.M and analyzed by one-way ANOVA followed by Tukey’s multiple range test (*n* = 3). The same letter represented no significant difference (*p* > 0.05), * *p* < 0.05.

To further investigate the role of ACC on fat deposition, CP-640186, a dual ACC1 and ACC2 inhibitor, was injected intraperitoneally into the large yellow croaker fed with HFD. Results showed that plasma TG was significantly decreased compared with the control group after 24 h injection (*p* < 0.05) (Figure 6A). In addition, TG content was significantly decreased after incubation with OA and CP-640186 for 24 h in primary hepatocytes (Figure 6B) (*p* < 0.05). RT-qPCR results showed that the CP-640186 inhibited the increased expression of lipogenesis genes induced by OA, including *acc1*, *fas*, *scd*, *elovl6* and *dgat2* (*p* < 0.05). Meanwhile, genes related to fatty acid oxidation (*acc2*, *cpt1*, *acads* and *acat2*) were significantly decreased after incubation with CP-640186 (*p* < 0.05), while the expression of *ehhadh* and *acat1* had no significant changes. CP-640186 further upregulated the expression of fatty acid transport-related genes, including *cd36*, *apob100*, *fatp1* (*p* < 0.05) (Figure 6C–E). These data implicated inhibiting ACC could regulate the lipid homeostasis in large yellow croakers.

## 4. Discussion

Excessive liver fat deposition in fish caused by a high-fat diet not only affects the healthy development of aquatic products, but also is not conducive to human food safety. ACC is considered as a therapeutic target for metabolic diseases in mammals [28]. However, few studies were reported on the role and regulation of ACC in fish. In the present study, the ORF of ACC1 and ACC2 in large yellow croakers were cloned and analyzed. The structure domain and subcellular localization of ACC were highly similar to mammals [29,30]. These observations suggested that the function of ACC is conserved from fish to mammals.

After cloning and identifying ACC, we explored the response of ACC in large yellow croakers to high fat conditions. The results of the present study showed high fat activated the expression of ACC and inhibited its phosphorylation, suggesting the increase in liver fat caused by high fat is likely related to the abnormal expression of ACC. Therefore, we further explored the regulatory mechanism of high fat to ACC. AMPK is considered to be a classical phosphorylation kinase of ACC in mammals [31,32]. In our study, AMPK significantly activated the phosphorylation of ACC, which means the AMPK/ACC signaling pathway is also applicable in fish. The present study indicated that high fat inhibited the phosphorylation of the AMPK/ACC pathway. AICAR significantly reduce the OA-induced increase in TG in large yellow croaker hepatocytes, which is consistent with previous studies [33,34].

AMPK is activated by LKB1 and CAMKK2 in mammals [35,36,37]. However, the change of LKB1 in large yellow croakers under high fat was not as obvious as that in mammals [38,39], indicating that there are other stronger regulation modes in the upstream of AMPK. O-GlcNAcylation and phosphorylation exhibit strong crosstalk due to the fact that they modify the same sites (Ser/Thr amino acids) [40]. AMPK also interrelated with O-GlcNAcylation in nutrition metabolism processes [41,42]. Previous study in mammals has confirmed the AMPK signaling pathway regulated the level of OGT transcripts in response to glucose deprivation [41]. In mouse embryonic fibroblasts, OGT knockdown reduced AMPK phosphorylation on Thr172, suggesting that O-GlcNAcylation upregulated AMPK activity [43]. In the present study, results showed O-GlcNAcylation agonist treatment could significantly inhibit the phosphorylation of AMPK. Furthermore, O-GlcNAcylation increased significantly after OA incubation in large yellow croaker hepatocytes. Consistent with this, recent studies have shown that levels of global O-GlcNAcylation in obese, type 2 diabetic people was higher compared with the control [44]. The current results indicated that the activation of global O-GlcNAcylation by high fat may inhibit AMPK/ACC phosphorylation and regulate lipid synthesis subsequently. These results provide new insights for revealing the mechanism of high fat-induced liver fat deposition in fish.

The above results demonstrated that ACC played an important role in the abnormal fat deposition induced by high fat. Finally, we explored whether the excessive fat deposition caused by high fat could be rescued by inhibiting ACC. A single intraperitoneal injection of ACC inhibitor significantly reduced the content of TG in the plasma of the HFD group, but it does not completely return to normal levels. This may be because the abnormal fat deposition was caused by the disorder of multiple metabolic processes. It is not ruled out that fatty acid intake or other ways affect the TG synthesis. However, studies in mammals have shown ACC inhibitor administered significantly reduced high-fat sucrose diet-induced hepatic steatosis, but increased plasma triglyceride [45]. The differences may be due to the duration of inhibition or diet sources. Apart from these discrepancies, the inhibition of ACC also has important effect on other lipid metabolism genes. CP-640186 inhibited the increase in lipogenesis genes induced by OA and further activated fatty acid transport-related genes. In HepG2 cells, the inhibition of ACC activity not only attenuated DNL and induced FAO, but also inhibited the synthesis of long chain saturated, mono- and polyunsaturated fatty acids [46]. The oral administration of ACC inhibitors inhibited DNL in the liver of non-alcoholic steatohepatitis patients, alleviated hepatic steatosis, and improved liver stiffness and injury [47]. Long term treatment with ACC inhibitors (21 days) significantly reduced high-fat sucrose diet-induced hepatic steatosis, and alleviated diet-induced nonalcoholic fatty liver disease and hepatic insulin resistance in rats [3]. Taken together, inhibiting the activity of ACC contributes to the regulation of lipid disorders of large yellow croakers under high fat conditions, which is in agreement with results in mammals. These results proved a strong regulatory effect of ACC on fat deposition, which could be considered as a target for regulating fat deposition in fish.

**Figure 7:**
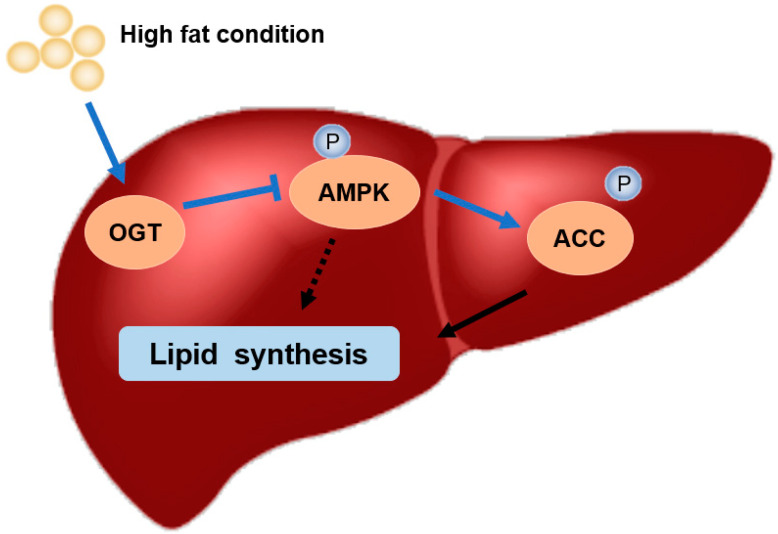
High fat condition activated the O-GlcNAcylation level and inhibit the phosphorylation of AMPK/ACC pathway. Inhibiting ACC regulated the lipid homeostasis of the liver of large yellow croaker.

In summary, we cloned and characterized the ACC1 and ACC2 genes and explored the role of ACC in large yellow croakers under high fat. Importantly, the present study demonstrated OA-decreased AMPK/ACC phosphorylation was associated with global O-GlcNAcylation for the first time (Figure 7). These results may contribute to the development of therapeutic strategies to alleviate high-fat-induced abnormal liver fat deposition and the experiment of green and healthy breeding.

## Data Availability

Not applicable.

## Supplementary Materials

The following are available online at https://www.mdpi.com/article/10.3390/nu13061740/s1, Figure S1: Nucleotide and deduced amino acid sequences of acc1 gene. Figure S2: Nucleotide and deduced amino acid sequences of acc2 gene. Figure S3: Multiple sequence alignment of the deduced AA sequences of large yellow croaker ACC1 with other fish species or mammals. Figure S4: Multiple sequence alignment of the deduced AA sequences of large yellow croaker ACC2 with other fish species or mammals. Table S1: Sequences of the PCR primers used in this study for gene cloning and qPCR.

## Abbreviations

ACC1, Acetyl CoA carboxylase 1; ACC2, Acetyl CoA carboxylase 2; FAS, fatty acid synthase; SCD1, stearoyl CoA desaturase1; ELOVL6, elongases of long-chain fatty acids 6; DGAT2, Diacylglycerol Acyltransferase 2; CPT1, carnitine palmitoyl transferase 1; ACADS, acyl-Coenzyme A dehydrogenase; EHHADH, enoyl-CoA hydratase/3-hydroxyacyl CoA dehydrogenase; ACAT1, Acetyl CoA Acetyltransferase 1; ACAT2, Acetyl CoA Acetyltransferase 2; CD36, cluster of differentiation 36; MTP, microsomal triglyceride transfer protein; APOB100, apolipoprotein B100; FATP1, fatty acid transport protein 1; FABP3, fatty acid binding protein 3; OGT, O-GlcNAc transferase; OGA, O-GlcNAcase; CAMKK2, calcium/calmodulin-dependent protein kinase kinase 2; LKB1, liver kinase 1; AMPK, Adenosine 5′-monophosphate (AMP)-activated protein kinase; GAPDH, glyceraldehyde-3-phosphate dehydrogenase.
