# Supplementary material for: High Fat Activates O-GlcNAcylation and Affects AMPK/ACC Pathway to Regulate Lipid Metabolism
Source: Nutrients. 2021 May 21;13(6):1740. doi: 10.3390/nu13061740 (PMC8223797; doi:10.3390/nu13061740)
Supplement: Supplementary file 1 [file nutrients-13-01740-s001.zip › Supplementary data.pdf]

Supplementary data

Table S1

Sequences of the PCR primers used in this study for gene cloning and qPCR.

Primer	Sequences	Primer Information
ACC1-ORF-F	ATGTTTCCCTGGCTCACGTTTG	ORF clone
ACC1-ORF-R	CTAGGAGGAGGCCGACGTCTCC	ORF clone
ACC2-ORF-F	ATGCTCCCTTTTGCAGTTTTGGG	ORF clone
ACC2-ORF-R	TTAACTGGTGCTGGTGCTGTCCAT	ORF clone
acc1-F	GACTTGGCGGAATACCTACTGG	qPCR
acc1-R	GCTTGCTGGATGATCTTTGCTT	qPCR
acc2-F	AAAGAATCCCTGTGCAGGCTGTC	qPCR
acc2-R	TCCTCCTCGGTCCAATCCACTC	qPCR
fas-F	CAGCCACAGTGAGGTCATCC	qPCR
fas-R	TGAGGACATTGAGCCAGACAC	qPCR
scd-F	AAAGGACGCAAGCTGGAACT	qPCR
scd-R	CTGGGACGAAGTACGACACC	qPCR
elovl6-F	TCAACGAGGACGAAGCCATACGA	qPCR
elovl6-R	CCCAGTGCGGACAGCACCAAATA	qPCR
dgat2-F	TTCGGTGCTTTCTGCAACTTCG	qPCR
dgat2-R	AAGGATGGGGAAGCGGAAGT	qPCR
cpt1-F	GCTGAGCCTGGTGAAGATGTTC	qPCR
cpt1-R	TCCATTTGGTTGAATTGTTTACTGTCC	qPCR
acads-F	ACGAGTGGGTGCTGAATGG	qPCR
acads-R	TGGATGAGGCTCTGATGCCCAACTT	qPCR
ehhadh-F	CCGTGGAGGTCCCATGTTCT	qPCR
ehhadh-R	GGCTGCCACTGGCCACGAGCCTCCT	qPCR
acat1-F	CGTTGCACCCATTGATTTCC	qPCR
acat1-R	GCCTCGTTGATCTCCCACAT	qPCR
acat2-F	CCTGACAGACGCCTTCCAT	qPCR
acat2-R	CCTTCACCTCCACTGAACCTTGTCT	qPCR
cd36-F	GAGCATGATGGAAAATGGTTCAAAG	qPCR
cd36-R	CTCCAGAAACTCCCTTTCACCTTAG	qPCR
mtp-F	CTTGAGTCGCTGATTGCTGC	qPCR
mtp-R	TGAGGTCGCTGTAACCCTTG	qPCR
apob100-F	AGAGTGTTGTCCAGGATAAAGATGC	qPCR
apob100-R	CAGGGCTCAGGGTCTCAGTC	qPCR
fatp1-F	CAACCAGCAGGACCCATTACG	qPCR
fatp1-R	CATCCATCACCAGCACATCACC	qPCR
fabp3-F	CCAAACCCACCACTATCATCTCAG	qPCR
fabp3-R	GCACCATCTTTCCCTCCTCTATTG	qPCR
ogt-F	ACATGGGTCTGCCTGGCTCTC	qPCR

ogt-R	ATGCCTGTAGTGTGACCGTTG	qPCR
oga-F	ACAGCCGACAGGTGCCTCAG	qPCR
oga-R	GCTGCTGGAACACCGTCGTAAC	qPCR
camkk2-F	TCACAGAGTACGAGCCCAGTAGAC	qPCR
camkk2-R	AGCAGGTCGCAGGAAGGAGTC	qPCR
lkb1-F	AAGAAGATCGGGATCAGGGGC	qPCR
lkb1-R	TGAGGGGTTGGATGAAGAGGA	qPCR
β-actin-F	GACCTGACAGACTACCTCATG	qPCR
β-actin-R	AGTTGAAGGTGGTCTCGTGGA	qPCR

ATGTTTCCCTGGCTCACGTTTGCGGCGTGTTTGGCGTCAGTGCTCGGCTTGTTTTTCTGGTCCCGCAAGCAGCTAACGTCGATAGTATTCGCAGCCTGTTCGTGTCGTCCAGCCATGGCA
M F P W L T F A A C L A S V L G L F F W S R K Q L T S I V F A A C S C R P A M A
CAGCAGGACGGTGCCGCCGCCGCTAAGAAGAACCCGGCCGTCGCGGCGTTGCACTCTCACTTTATCGTGGGCTCTGTGTCGGAGGAGAACTCGGAAGATGAAATCCAAGGGAAGCCTGAC
Q Q D G A A A A K K N P A V A A L H S H F I V G S V S E E N S E D E I Q G K P D
GTTCAGCTGGAGGAAAAGGAGAATCGGTCCTTGTCGCCGTCCTCCGCTAGCTCAGACAGCACCTGTGAAATGGGATTTGATCACATTGATGGCCCCGTGCAGAATCTAAGGCCGAGCATG
V Q L E E K E N R S L S P S S A S S D S T C E M G F D H I D G P V Q N L R P S M
TCAGGGCTGCACCTGGTGAAGCAAGGCAGAGATCGCCGCCGCATCGATCTCCAGAGAGACTTCACCGTGGCTTCTCCTGCTGAATTTGTCACCCGCTTCGGTGGCAATAAAGTTATCGAG
S G L H L V K Q G R D R R R I D L Q R D F T V A S P A E F V T R F G G N K V I E
AAGGTGCTTATCGCCAACAATGGCATTGCAGCAGTCAAATGCATGCGCTCCATCCGCCGGTGGGCCTATGAGATGTTCCGCAATGAAAGGGCGATCCGCTTCGTCGTTATGGTGACCCCA
K V L I A N N G I A A V K C M R S I R R W A Y E M F R N E R A I R F V V M V T P
GAGGACCTGAAAGCCAACGCGGAGTACATTAAAATGGCAGATCATTACGTGCCTGTGCCAGGAGGGACTAACAACAACAACTACGCCAACGTGGAGCTCATTCTAGACATTGCTAAACGC
E D L K A N A E Y I K M A D H Y V P V P G G T N N N N Y A N V E L I L D I A K R
ATACCTGTTCAGGCGGTGTGGGCCGGGTGGGGTCATGCTTCAGAGAACCCGAAACTTCCAGAGCTGCTTCACAAGCACGGCATTGCTTTCATGGGTCCCCCAAGTCAGGCTATGTGGGCT
I P V Q A V W A G W G H A S E N P K L P E L L H K H G I A F M G P P S Q A M W A
CTGGGAGACAAGATTGCTTCGTCTATTGTGGCTCAGACAGCTGGCATTCCCACCCTGCCATGGAGCGGCACAGGCCTTTCGGTAGAATGGACAGAGAACAACCAAAAGAAGAAAGTTATC
L G D K I A S S I V A Q T A G I P T L P W S G T G L S V E W T E N N Q K K K V I
AACGTCCCTACTGACGTGTATGAGCTTGGCTGCATCCAGGATGTCGAGGATGGCCTTAAAGCTGCAGAGAAGATCGGCTACCCTGTGATGGTGAAGGCCTCTGAGGGAGGCGGCGGAAAA
N V P T D V Y E L G C I Q D V E D G L K A A E K I G Y P V M V K A S E G G G G K
GGCATCCGTAAAGTGAACTGCGTCGATGATTTCCCAAACCTCTTCCGACAGGTCCAGGCAGAAGTTCCAGGCTCGCCTGTTTTCGTCATGCAGCTAGCCAAACACGCCCGCCATCTGGAG
G I R K V N C V D D F P N L F R Q V Q A E V P G S P V F V M Q L A K H A R H L E
GTCCAGATCTTGGCTGATCAGTATGGCAATGCCATTTCCCTGTTTGGTCGTGACTGCTCCGTGCAGCGACGGCATCAGAAAATTATAGAGGAAGCCCCCGCTACCATCGCGACCTCTGAT
V Q I L A D Q Y G N A I S L F G R D C S V Q R R H Q K I I E E A P A T I A T S D
GTGTTTGAGGATATGGAAAGGTGTGCAGTGAAGCTGGCTAAGATGGTGGGATACGTCAGCGCAGGTACAGTGGAGTACCTCTATAGCCAGGACGGCAGCTTCTACTTCCTGGAGCTCAAC
V F E D M E R C A V K L A K M V G Y V S A G T V E Y L Y S Q D G S F Y F L E L N
CCCCGTCTGCAGGTGGAGCACCCCTGCACCGAGATGGTGGCTGACGTCAACTTGCCTGCTGCCCAACTGCAGATTGCTATGGGTATTCCTCTTCAAAGGATCAAAGACATCAGGATGCTT
P R L Q V E H P C T E M V A D V N L P A A Q L Q I A M G I P L Q R I K D I R M L
TATGGAGTCCAGCCCTGGGGAGACTCACCCATTGACTTCGAGGGTCTGTCGACAGCCCCCTGCCCACGGGGCCACGTCATTGCAGCACGTATCACCAGTGAAAATCCTGATGAGGGTTTC
Y G V Q P W G D S P I D F E G L S T A P C P R G H V I A A R I T S E N P D E G F
AAGCCAAGCTCCGGAACAGTGCAAGAGTTGAATTTCCGCAGCAATAAAAACGTGTGGGGCTACTTCAGCGTGGCAGCGGCTGGAGGCCTCCACGAGTTCGCTGACTCCCAGTTTGGACAC
K P S S G T V Q E L N F R S N K N V W G Y F S V A A A G G L H E F A D S Q F G H
TGTTTCTCTTGGGGAGAAAATCGTGAAGAAGCCATCTCCAACATGGTGGTGGCTCTGAAGGAGCTGTCTATCAGAGGAGACTTCAGGACCACAGTCGAATACCTCATTAAGCTGCTGGAG
C F S W G E N R E E A I S N M V V A L K E L S I R G D F R T T V E Y L I K L L E
ACGGAAAGCTTTCAGCACAACAGCATCGACACTGGCTGGCTGGACAGGCTGATCTCAGAGAAGATGCAGGCGGAGCGCCCTGACACCATGCTGGGAATCGTGAGTGGGGCTCTTCACGTG
T E S F Q H N S I D T G W L D R L I S E K M Q A E R P D T M L G I V S G A L H V
GCAGATGTCAATCTGAGGAACAGTGTCTCCAACTTCCTGCATTCTCTAGAAAGGGGCCAGGTGCTGCCGGCACACACACTACTCAACACTGTGGATGTGGAGCTGATCTATGAAGGCACT
A D V N L R N S V S N F L H S L E R G Q V L P A H T L L N T V D V E L I Y E G T
AAGTACGTCCTGACGGTGACGCGCCAGTCTCCCAACTCCTACGTGGTCATCATGAACAACTCCTCCGCTGAGGTGGACGTCCATCGGCTCAGCGATGGAGGTCTTTTGCTGTCTTATGAC
K Y V L T V T R Q S P N S Y V V I M N N S S A E V D V H R L S D G G L L L S Y D
GGAAGCAGCTACACTACATACATGAAAGAAGAGGTGGACAGGTATCGCATCACAATTGGGAACAAGACTTGTGTTTTCGAAAGGGAGAACGACCCTTCGCTGCTACGATCTCCTTCAGCG
G S S Y T T Y M K E E V D R Y R I T I G N K T C V F E R E N D P S L L R S P S A
GGAAAACTCATCCAGTACACAGTCGAGGATGGCGGACATGTGTTTTCTGGCCAGTGCTACGCTGAAATAGAGGTGATGAAGATGGTAATGACCCTTACGGCTGCAGAGTCTGGATGTATT
G K L I Q Y T V E D G G H V F S G Q C Y A E I E V M K M V M T L T A A E S G C I
CACTATGTGAAGAGAGCTGGAGCGGCACTGGATCCAGGCTGTGTGATTGCCAAGCTGCAACTGGATGACCCAAGCCGAGTGCAACAGGCAGAGCTGCACACAGGGGCCTTGCCTTCTATC
H Y V K R A G A A L D P G C V I A K L Q L D D P S R V Q Q A E L H T G A L P S I
CAGGCAGTAGCTCTGAGAGGAGAGAAGCTGCACAGAGTTTTCCACAACACACTGGATCATCTCGTTCACATAATGAACGGCTACTGTCTCCCTGAGCCTTTCTTCAGTGCTAAACTGAAA
Q A V A L R G E K L H R V F H N T L D H L V H I M N G Y C L P E P F F S A K L K
GAGTGGGTGGAAAGGTTGATGAAAACCATGCGTGATCCCTCTTTGCCACTGTTGGAACTCCAAGACATCATGACCAGTGTGTCTGGTCGCATCCCTCCTGCCGTGGAGAAGGCTATCAAG
E W V E R L M K T M R D P S L P L L E L Q D I M T S V S G R I P P A V E K A I K
AAAGAAATGGCTCAGTATGCGAGCAACATCACCTCCGTGCTCTGCCAGTTTCCCAGCCAGCAGATCGCGAACATCCTGGACAGCCATGCTGCTACTCTAAACAAGAAGTCAGAGAGAGAA
K E M A Q Y A S N I T S V L C Q F P S Q Q I A N I L D S H A A T L N K K S E R E
GTCTTCTTTATGAACACACAAAGCATTGTTCAGCTGGTACAGAAATATCGCAGTGGCATCCGAGGTCACATGAAGGCCGTGGTGATGGACCTGCTGAGACAGTACCTGAAAGTAGAGATC
V F F M N T Q S I V Q L V Q K Y R S G I R G H M K A V V M D L L R Q Y L K V E I
CAGTTTCAGAATGGACACTACGACAAATGTGTGTTCGCACTACGTGAGGAAAACAAAGGCGACATGTCCAATGTGCTCAACTATATCTTCTCCCATGCTCAAGTCACCAAGAAGAACCTG
Q F Q N G H Y D K C V F A L R E E N K G D M S N V L N Y I F S H A Q V T K K N L
CTGGTTACCATGCTGATTGACCAGCTCTGTGGCCGCGATCCCACGCTGACAGATGAACTGATGGCCATCTTGACTGAACTCACCCAGCTCAGCAAAACGACCAACGCCAAGGTGGCACTG
L V T M L I D Q L C G R D P T L T D E L M A I L T E L T Q L S K T T N A K V A L
CGTGCCCGACAGGTGTTGATCGCTTCTCATCTCCCCTCATACGAGCTACGACACAACCAGGTGGAGTCCATCTTCCTCTCTGCCATCGATATGTACGGGCACCAGTTCTGCATTGAAAAC
R A R Q V L I A S H L P S Y E L R H N Q V E S I F L S A I D M Y G H Q F C I E N
CTGCAGAAACTGATCCTTTCGGAAACATCCATCTTTGATGTTCTGCCCAACTTCTTCTACCACAGCAATCAGGTAGTCAGGATGGCTGCCCTTGAGGTGTACGTTCGCAGAGCATACATT
L Q K L I L S E T S I F D V L P N F F Y H S N Q V V R M A A L E V Y V R R A Y I
GCGTATGAGCTGAACAGCGTTCAGCATCGGCAGCTGAGGGACAACACGTGTATAGTAGAGTTCCAGTTCATGCTTCCCACCTCGCACCCAAACAGAGGGAACATCCCCACTCTAAACAGG
A Y E L N S V Q H R Q L R D N T C I V E F Q F M L P T S H P N R G N I P T L N R
ATGTCATTCTCGTCTAACCTAAACCACTACGGCATGGTGCATGTAGCCAGCGTCAGTGACGTTCTGCTTGACACATCTTTTACACCACCTTGTCAGCGCATGGGAGCCATGGTCGCTTTC
M S F S S N L N H Y G M V H V A S V S D V L L D T S F T P P C Q R M G A M V A F
CGCTCCTTCCAGGAGTTCACAAGGAACATCGCAGACGTGTTGAGCTGCTTCTCCGACTCTCCTCCCCCAAGTCCAACCTTCCCAGAGGGAGGTAATCCTGTCCTGTATGGCGAAGAGGAC
R S F Q E F T R N I A D V L S C F S D S P P P S P T F P E G G N P V L Y G E E D

AACAAGAGTGTCCAGGACGAGCCTATCCATATCTTGAATGTGGCTATAAAGACCGACAGCGACATCGACGATGACGGCCTTGCAGCCAACTTTAGAGAGTTCACTCAGTCAAAGAAATCG

N K S V Q D E P I H I L N V A I K T D S D I D D D G L A A N F R E F T Q S K K S

CTGCTGTTTGAACACGGAATCCGTAGGCTGACTTTCCTTGTGGCTCAGAAGGATTTCAGGAAGCAAGTCAACTGTGAGGTGGACCAAAGGTTTCATAGAGAATTCCCAAAATTTTTCACA

L L F E H G I R R L T F L V A Q K D F R K Q V N C E V D Q R F H R E F P K F F T

TTCCGCGCCAGAGATAAGTTCGAAGAGGACAGGATCTATCGTCATCTAGAGCCGGCGCTCGCTTTCCAGTTGGAGCTCAACCGCATGCGCAATTTCGCCTTAACTGCCATCCCGTGCGCC

F R A R D K F E E D R I Y R H L E P A L A F Q L E L N R M R N F A L T A I P C A

AACCACAAGATGCACCTGTACCTGGGTGCAGCCCGCGTGGAGGTGGGCACAGAGGTTACGGACTACCGTTTCTTTGTGCGAGCCATTATCCGCCACTCTGATCTGGTCACGAAGGAGGCC

N H K M H L Y L G A A R V E V G T E V T D Y R F F V R A I I R H S D L V T K E A

TCTTTTGAATACCTTCACAATGAGGCAGAGCGTCTGCTGCTGGAAGCCATGGATGAGCTGGAGGTGGCTTTCAACAACACAACTGTACGGACCGACTGCAATCACATCTTCCTCAATTTT

S F E Y L H N E A E R L L L E A M D E L E V A F N N T T V R T D C N H I F L N F

GTCCCCACAGTCATTATGGACCCATCAAAGATCGAGGAGTCCGTGCGCTCCATGGTGATGCGTTACGGCAGCCGTCTGTGGAAGCTGCGCGTCCTGCAGGCCGAGCTGAAAATCAACATC

V P T V I M D P S K I E E S V R S M V M R Y G S R L W K L R V L Q A E L K I N I

CGCCTGACTCCAACAGGAAAGCAAATCCCCATCCGCCTCTTCCTCACTAATGAATCGGGCTACTATCTAGACATCAGCTTGTACAAGGAGGTCACTGATTCCCGAACGGGACAGGTGGGG

R L T P T G K Q I P I R L F L T N E S G Y Y L D I S L Y K E V T D S R T G Q V G

CCCAAAGACCGACAGATCATGTTCCAAGCATATGGAGACAAGCAGGGTCCGTTGCATGGCATGCTCATCAACACCCCGTATGTCACCAAGGATCTGCTGCAGTCGAAGCGCTTCCAGGCA

P K D R Q I M F Q A Y G D K Q G P L H G M L I N T P Y V T K D L L Q S K R F Q A

CAGTCTCTGGGCACCACCTACGTCTACGACTTCCCAGAAATGTTCAGACAGGCTCTAAAAAAGCTGTGGCACTCCAGCCAGGCCTTTGCCCACTTACCCAGATGCCCCCTCCCTTCTGAG

Q S L G T T Y V Y D F P E M F R Q A L K K L W H S S Q A F A H L P R C P L P S E

CTGCTCACATTCACAGAGCTGGTTCTCGACGCCCAAGGTCAGCTGGTGCAGATGAACCGACTGCCAGGGGGCAACGAGATTGGTATGGTGGCATGGCGGATGACCCTGCGCACGCCAGAA

L L T F T E L V L D A Q G Q L V Q M N R L P G G N E I G M V A W R M T L R T P E

TATCCGGCGGGACGCGAGATCATCGTCATAAGTAATGACATCACGCACAAGATTGGCTCGTTCGGGCCCCAGGAGGACGTGCTGTTCCTGCGAGCTTCAGAGATGGCCCGCGAGAGCGGC

Y P A G R E I I V I S N D I T H K I G S F G P Q E D V L F L R A S E M A R E S G

ATCCCGCGGATCTACATCGCAGCCAACAGCGGCGCCCGCATCGGGCTGGCAGAGGAAATCAGACACATGTTCCACGTGGCCTGGCAAGATCCAGCTGACCCCTATAAGGGCTTCAAGTAT

I P R I Y I A A N S G A R I G L A E E I R H M F H V A W Q D P A D P Y K G F K Y

CTCTACCTCACACCTCAGGATTACAAGAAAGTTTCAGCCCTGAACTCCGTGCATTGTGAACATGTGGAAGATGAGGGAGAATCCAGGTACAAGATCACCGACATCATAGGAAAAGACGAA

L Y L T P Q D Y K K V S A L N S V H C E H V E D E G E S R Y K I T D I I G K D E

GGGCTGGGTGTGGAGAATCTGAAAGGGTCTGGAATGATTGCCGGAGAATCCTCTCTGGCTTACGAGGAGATCATCACCATGAACCTGGTCACATGCAGAGCCATAGGAATCGGGGCCTAT

G L G V E N L K G S G M I A G E S S L A Y E E I I T M N L V T C R A I G I G A Y

TTGGTGAGGCTCGGGCAGAGAACCATTCAAGTGGACAACTCTCATATTATTCTTACTGGAGCTGGAGCACTCAACAAGGTGCTGGGTAGAGAAGTCTACACATCCAACAACCAGCTTGGT

L V R L G Q R T I Q V D N S H I I L T G A G A L N K V L G R E V Y T S N N Q L G

GGAATCCAAATCATGCACAACAACGGCGTGACCCACTGCACCGTTTGTGACGACTTTGAGGGAGTCTTCACACTTCTGCAGTGGCTGTCCTACATGCCCATGTGCAAATCTAGTCCGGTG

G I Q I M H N N G V T H C T V C D D F E G V F T L L Q W L S Y M P M C K S S P V

CCTATCGTCCATTCCAAGGATCCCATAGATCGGCCGGTGGAGTTTGTGCCTACAAAGGCTCCCTACGACCCTCGCTGGATGTTAGCGGGGCGTCCCAGCCAGACTCCAAAGGGTTCCTGG

P I V H S K D P I D R P V E F V P T K A P Y D P R W M L A G R P S Q T P K G S W

CAGAATGGCTTCTTTGACCATGGTTCCTTCATGGAAATCATGCAGCCTTGGGCTCAGAGTGTGGTGGTAGGCAGAGCCAGACTTGGCGGAATACCTACTGGAGTGGTTGCCGTGGAAACC

Q N G F F D H G S F M E I M Q P W A Q S V V V G R A R L G G I P T G V V A V E T

AGGTCAGTGGAGCTCTCCATCCCCGCCGATCCGGCCAATTTGGACTCAGAAGCAAAGATCATCCAGCAAGCAGGACAGGTGTGGTTCCCAGATTCTGCTTTCAAAACAGCTCAGGCTATT

R S V E L S I P A D P A N L D S E A K I I Q Q A G Q V W F P D S A F K T A Q A I

AAGGACCTGAACAGGGAGGGCCTACCTCTCATAGTGTTTGCCAACTGGAGGGGCTTTTCTGGAGGAATGAAAGATATGTACGACCAGGTGTTGAAGTTCGGGGCCTACATCGTGGACGGG

K D L N R E G L P L I V F A N W R G F S G G M K D M Y D Q V L K F G A Y I V D G

TTGAGGGAGTACCAGCAGCCGGTGCTGGTTTATATCCCCCCACAGGCTGAGCTGAGGGGAGGATCCTGGGTAGTTATAGATCCAACCATCAACCCTCGTCACATGGAGATGTACGCTGAC

L R E Y Q Q P V L V Y I P P Q A E L R G G S W V V I D P T I N P R H M E M Y A D

AAGGACAGCCGAGGTGGAGTGTTGGAGCCCGAGGGAACAGTGGAGATCAAGTTTAGGAGGAAGGACCTGGTGAAGACCATGAGAAGAGTAGATCCTGTCTACATGGGCTTGGCTGAAAAA

K D S R G G V L E P E G T V E I K F R R K D L V K T M R R V D P V Y M G L A E K

TTGGGAACCCCAGAGCTGAGTCCCCCCGATCGTAAAGAGCTGGAGACCAAGCTGAAGGAGCGTGAGGAGTTTCTCCTGCCCATCTACCACCAGGTTGCTGTGCAGTTCGCTGACCTCCAC

L G T P E L S P P D R K E L E T K L K E R E E F L L P I Y H Q V A V Q F A D L H

GACACACCAGGTCGCATGCAAGAGAAGGGTGTAATAACGGACATCCTTGAATGGCAAACGTCCCGTCAGTTCTTTTACTGGCGTCTGCGGCGCCTGCTGCTGGAGGAGACGGTGAAGAGA

D T P G R M Q E K G V I T D I L E W Q T S R Q F F Y W R L R R L L L E E T V K R

AAGATCCAAGCGGCCAACAGCGAGCTGACAGACGGTCAGGTCCAAGCGATGCTGCGCCGCTGGTTCGTAGAGGCCGAGGGTGCGGTCAAGGCCTATCTGTGGGATAACAATGAAGAGGTG

K I Q A A N S E L T D G Q V Q A M L R R W F V E A E G A V K A Y L W D N N E E V

GTGGGATGGCTGGAGAGGCAACTAGCTGAAGAAGAGGGCGCGAGGTCCGTCATCGACGAGAACATCAAGTACATCCGCCGAGATCACATCCTCAAGCAGATACGCAGCCTTGTCCAAGCC

V G W L E R Q L A E E E G A R S V I D E N I K Y I R R D H I L K Q I R S L V Q A

AATCCAGAGGTCGCCATGGATTCCATTGTGCACATGACCCAGCACATCTCGCCCACGCAGAGAACCGAGGTGGTACGTATCCTGTCCACCATGGAGACGTCGGCCTCCTCC**TAG**

N P E V A M D S I V H M T Q H I S P T Q R T E V V R I L S T M E T S A S S *

Figure S1 Nucleotide and deduced amino acid sequences of *acc1* gene. The start codon (ATG) and the stop codon (TGA) are in bold.

ATGCTCCCTTTTGCAGTTTTGGGATTAATCCTATGGATTTCATTGCTACTGTGGAGGATTATCACCAAGGCAGTAGCGATGCCCGTGAAAGGAGAGGAATCTCCGTCTGGCTGTGGTCCG
M L P F A V L G L I L W I S L L L W R I I T K A V A M P V K G E E S P S G C G P
CCTGCAGCTGAGGAGGACATGCCTGTTACACAATCCCCTCATCCAGGCGAACATTCCCTGTCAACAGCCCCTACTACCTCAGCGCACAATGAGGATAACAGTTCTCAGGCCGGCTGTGCC
P A A E E D M P V T Q S P H P G E H S L S T A P T T S A H N E D N S S Q A G C A
CCTGCAGGTCAAGTTAAAGCAGTGGACACAGAGGCTCTGCAGGAGTCCTCTGAGGTAAATTCAGAAAAACCACCAACCACACAGACATCTTCCAAAACCAAAGTGCCAATGCTCAAATCA
P A G Q V K A V D T E A L Q E S S E V N S E K P P T T Q T S S K T K V P M L K S
GGGCCTGAGGGGAGGGAACGCCTCAAGTTCATTCTCGGAGCATCTGAGGATAACTCTTCAGACGAGGAGCCTCTGGTTAGCAAACCTCCAAGTGGCGCATCTCAGCCACCGGCCTCCACA
G P E G R E R L K F I L G A S E D N S S D E E P L V S K P P S G A S Q P P A S T
AGCAAATCTTATCCCCAGGAGGGATCCACAGCAGCACGACGATCCAGCTCCTCAGGCATCAAGCCTAGCATGTCTGGTCTTCACTTGGTGAGAAAAGGACGCGAACACAGAAAGATGGAT
S K S Y P Q E G S T A A R R S S S S G I K P S M S G L H L V R K G R E H R K M D
CTCCAGAGGGACTTCACTGTGGCCTCTCCTGCTGAGTTTGTCACTAGATTTGGTGGCAACCGTGTCATTGAAAAAGTGCTGATAGCTAACAATGGCATTGCTGCGGTAAAATGTATGCGC
L Q R D F T V A S P A E F V T R F G G N R V I E K V L I A N N G I A A V K C M R
TCTATCCGTCGCTGGTCCTACGAAATGTTTCGCAACGAGAGGACCATCCGTTTTGTGGTCATGGTAACCCCTGAAGACCTGAAAGCAAATGCAGAATACATTAAAATGGCAGACCATTAT
S I R R W S Y E M F R N E R T I R F V V M V T P E D L K A N A E Y I K M A D H Y
GTTCCTGTACCCGGTGGGCCCAACAATAACAACTACGCCAACGTAGAGCTGATAGTAGACATTGCTAAAAGAATCCCTGTGCAGGCTGTCTGGGCTGGATGGGGTCATGCCTCTGAAAAT
V P V P G G P N N N N Y A N V E L I V D I A K R I P V Q A V W A G W G H A S E N
CCCAAACTGCCTGAGCTGTTGAACAAAGCAGGAATCTCATTTTTAGGACCGTCCAGTAAAGCCATGTGGGCTCTTGGAGATAAAGTGGCTTCTTCTATCGTGGCTCAGAGTGCTGACATT
P K L P E L L N K A G I S F L G P S S K A M W A L G D K V A S S I V A Q S A D I
CCCACACTACCATGGAGCGGATCAGGTCTAAGAGTGGATTGGACCGAGGAGGACCAAAAACTGGGCAACGTAATCAGTGTTCCTTCAGAGATCTATGCAAACGGCTGTGTTCGGGATGTT
P T L P W S G S G L R V D W T E E D Q K L G N V I S V P S E I Y A N G C V R D V
GATGATGGGCTGGCAGGGGCTGAGAGAATTGGTTATCCGGTTGTTATCAAGGCCTCTGAGGGTGGAGGTGGAAAAGGCATCCGTAAAGTAGAATGCTCTGAGGATTTTCCAGGTTTCTTT
D D G L A G A E R I G Y P V V I K A S E G G G G K G I R K V E C S E D F P G F F
AGACAGGTTCAGACAGAGGTACCCGGTTCCCCTATCTTCGTCATGCAGCTAGCTCAGCACGCCAGACACCTTGAGGTTCAGATACTGGCTGATGAGTATGGAAATGCCATCTCTCTGTTT
R Q V Q T E V P G S P I F V M Q L A Q H A R H L E V Q I L A D E Y G N A I S L F
GGACGAGACTGCTCCATCCAGAGGAGGCACCAGAAGATTATAGAGGAGGCTCCGGCCACCATTGCTGCTACCTCAACATTCGAGCAAATGGAATGGTGTGCTGTGCGACTGGCCAAGATG
G R D C S I Q R R H Q K I I E E A P A T I A A T S T F E Q M E W C A V R L A K M
GTTGGCTACGTGAGCGCAGGTACCGTGGAATATCTCTTCTCTGAAGACGGAAATTTCCATTTCCTGGAGCTGAATCCTCGCCTGCAGGTGGAACATCCCTGTACAGAGATGATCGGAGAT
V G Y V S A G T V E Y L F S E D G N F H F L E L N P R L Q V E H P C T E M I G D
GTAAACCTGCCAGCTGCCCAGCTTCAGATTGCGATGGGCATCCCCCTTTATAGAATTAAGGACATCCGTCTACTTTATGGAGAGACTCCGTGGGGTGATACCATCATTAACTTTGAAACT
V N L P A A Q L Q I A M G I P L Y R I K D I R L L Y G E T P W G D T I I N F E T
CCAGACTGCATACCAAGTCCAAGAGGGCACGTCATAGCTGCTCGGATCACCAGTGAGAACCCTGATGAGGGGTTTAAGCCCAGTTCGGGCACAGTGCAGGAGCTGAACTTCCGCAGCAGT
P D C I P S P R G H V I A A R I T S E N P D E G F K P S S G T V Q E L N F R S S
AAAAACGTGTGGGGTTATTTCAGCGTGGGAGCAACTGGTGGCCTGCATGAATTTGCCGACTCCCAGTTTGGACACTGTTTCTCCTGGGGAGAGAATCGTGAAGAAGCCATTTCGAACATG
K N V W G Y F S V G A T G G L H E F A D S Q F G H C F S W G E N R E E A I S N M
GTCGTGGCTATGAAGGAGCTGTCCATCAGAGGTGACTTCAGGACCACGGTTGAGTACCTCATCAAGTTACTGGAGACAGAATGCTTCAGAAACAATGACATCGACACGGGGTGGCTGGAT
V V A M K E L S I R G D F R T T V E Y L I K L L E T E C F R N N D I D T G W L D
CATCTGATTGCAGAGAAAGTGCAGGCGGAGAGACCAGATACGATGCTGGGGATTGTTTGTGGGGCTTTGCATGTTGCTGATGCGAGCTTTCGAAAGAGCATGTCTGATTACCTGCATTCA
H L I A E K V Q A E R P D T M L G I V C G A L H V A D A S F R K S M S D Y L H S
CTTGAAAGAGGCCAAGTACTGCCTGCGGCCAGTCTCCTCAACTCTGTCAGCGTGGACCTAATATATGAAGGAGTCAAGTACTGCCTCAAGGTGGCTCGCCAATCTCCGACAACTTACGTC
L E R G Q V L P A A S L L N S V S V D L I Y E G V K Y C L K V A R Q S P T T Y V
ATCATGATGAACGGCTCCAACATCGAGATAGATGTCCACAGGTTGAGCGACGGTGGCCTCTTGCTGTCCTATGACGGCAGCAGCCACACCACCTACATGAAGGAGGAAGTGGACAGCTAC
I M M N G S N I E I D V H R L S D G G L L L S Y D G S S H T T Y M K E E V D S Y
CGCATCACTGTTGGCAACAAGACGTGTGTATTTGAGAAGGAGAAGGATCCCACGGTGCTGAGGTCGCCCTCTGCTGGCAAACTGCTTCAGTACATCTGTGAGGATGGAGCTCACGTTTGT
R I T V G N K T C V F E K E K D P T V L R S P S A G K L L Q Y I C E D G A H V C
GCAGGAGAGACCTACGCGGAGATCGAGGTCATGAAGATGGTGATGACATTGACTGTGCAGCAGTCTGGTTGTATTCATTTTGTCAAGAGACCTGGAGCTGTTCTGGAGCCGGGCTGTGTG
A G E T Y A E I E V M K M V M T L T V Q Q S G C I H F V K R P G A V L E P G C V
GTGGCACACATAGAGCTAGATGACCCGAGCAGTATACATCTGGTGGAGCTCAACACGGCCATACTACCACCACAGCAACCCCTGCCCATTGTTGGGGAAAAGCTTCACCAGGTGTTTCAC
V A H I E L D D P S S I H L V E L N T A I L P P Q Q P L P I V G E K L H Q V F H
AGTGTGCTGGAAAACTTGGGTAAAGTCATGGATGGTTACTGCCTTGAGGAGCCGTTATTCAGCAGCAAGCTCAAACAGTGGGTGACTACCCTGATGAAGACTTTAAGGGACCCCTCACTA
S V L E N L G K V M D G Y C L E E P L F S S K L K Q W V T T L M K T L R D P S L
CCGCTGCTGGAACTTCAGGAGATCATGACGAGTGTGGGGGGTCGGATTCCTCCCGGTGTAGAGAAAGATATCCGTAAAGTCATGGCTCAGTATGCGAGCAACATCACCTCTGTCCTCTGC
P L L E L Q E I M T S V G G R I P P G V E K D I R K V M A Q Y A S N I T S V L C
CAGTTCCCTAGCCAAAGGATTGCAAACGTTTTAGACAGCCATGCAGCAACCCTGCAGAGGAAAGCTGATCGCGAGGTGTTCTTCATGAACACTCAGAGTATTGTTCAGCTGGTGCAGAGG
Q F P S Q R I A N V L D S H A A T L Q R K A D R E V F F M N T Q S I V Q L V Q R
TACCGCAGTGGAATCCGTGGTTATATGAAGTCTGTGGTTCTCGATCTGCTGAAGCGATACCTGCAAGTAGAGATGCAGTTTCAGCAAGCTCACTATGACAAGTGTGTCATCAACTTGAGA
Y R S G I R G Y M K S V V L D L L K R Y L Q V E M Q F Q Q A H Y D K C V I N L R
GAGCAGCACAAACCTGACATGAGTCGCGTGCTGGAGTACATCTTCTCTCATGCCCAAGTCTCCAAGAAGAACATCCTTGTCACAATGCTCATAGACCAGCTGTGTGGAAGAGATTCAACG
E Q H K P D M S R V L E Y I F S H A Q V S K K N I L V T M L I D Q L C G R D S T
CTAGCAGAAGAGCTGATGGCCATTTTGAACGAGCTCACGCAGCTTAGCAAGATGGAGAACTCAAAGGTTGCCTTGAGAGCCAGACAGGTTTTGATTGCCTCCCATTTACCATCATACGAA
L A E E L M A I L N E L T Q L S K M E N S K V A L R A R Q V L I A S H L P S Y E
CTGAGGCACAACCAGGTGGAGTCCATCTTCCTGTCAGCCATCGACATGTACGGCCACCAGTTCTGTCCGGAAAACTTGAAGAAACTTATCCTCTCTGAAACCTCCATTTTTGACGTTTTG
L R H N Q V E S I F L S A I D M Y G H Q F C P E N L K K L I L S E T S I F D V L
CCCAATTTCTTCTATCACTCCAATCAAGTTGTCTGCATGGCTGCCCTGGAGGTGTACGTACGCAGAGCTTACATCGCCTATGAGCTGAATAGCATCCAGCATCACCAGCTGCAGGATGGA
P N F F Y H S N Q V V C M A A L E V Y V R R A Y I A Y E L N S I Q H H Q L Q D G
TCGTGCGCTGTAGACTTCCAGTTCATGCTGCCGTCATCACATCCGAACAGAGGGAGCAGCCCTACTCTGAACAGGGTTCCCGTGCCAGTCAGTGGATCAGGCCAGTTTAAAATGAGGCGA
S C A V D F Q F M L P S S H P N R G S S P T L N R V P V P V S G S G Q F K M R R

CAGAGCAGTGAGCTCTTCCTGGAGGGAGCCTTGTCTCCACCCTGCCAGCGCATGGGCGCCATGGTGGCTTTCCAGTGTTTTGATGACTTCAAAAGGAATGTCGATGAAGTTCTCTCCAGC

Q S S E L F L E G A L S P P C Q R M G A M V A F Q C F D D F K R N V D E V L S S

TTTGCAGAACCACTCTTAGAGAGCTCTCCGTTCTCAGAGGCCTGTTCCAGTCTCTATGAGGATGAGAACCTCAAGAATACGAGGGAGAACCCAATCCACATCATTAATGTGTCCATAAAA

F A E P L L E S S P F S E A C S S L Y E D E N L K N T R E N P I H I I N V S I K

ACAGCCGACACTGAAGATGACGATGCCTTGGTCACAGCCCTCACTGCCTTTGCCCAGTCAAAGAAAGCTGTCCTCTTTGAGTACGGAATAAGGAGAATCACATTTTTGATTGCACAGAAG

T A D T E D D D A L V T A L T A F A Q S K K A V L F E Y G I R R I T F L I A Q K

AGAGAATTTCCCAAGTTCTTCACCTTCAGAGCTAGAGATGGGTTCCAGGAGGATCGCATTTATCGTAATCTGGAGCCAGCTTTAGCGTTCCAGCTGGAGCTCAACCGCATGAGGAACTTT

R E F P K F F T F R A R D G F Q E D R I Y R N L E P A L A F Q L E L N R M R N F

GACCTGACGGCCGTTCCCTGCGCCAACCACAAGATGCAACTCTACCTCGGTGCTGCTCGTGTCCAGGAGGGGGCAGAAGTCACGGACTACCGATTCTTCATACGAGCTATTATCCGCCAC

D L T A V P C A N H K M Q L Y L G A A R V Q E G A E V T D Y R F F I R A I I R H

TCAGATCTCATTACAAAGGAAGCCTCCTTTGAATACCTTCAAAATGAAGGAGAACGTCTCCTGTTGGAGGCCATGGATGAGTTGGAGGTGGCCTTCAGTAACACCAGCGTCCGCACAGAC

S D L I T K E A S F E Y L Q N E G E R L L L E A M D E L E V A F S N T S V R T D

TGCAACCATATCTTCCTCAACTTCGTCCCCACTGTCATCATGGACCCCTCTAAAATAGAGGAGTCTGTTCGCTCCATGGTGATGCGCTATGGCAGCCGTCTTTGGAAGCTGAGGGTCTTG

C N H I F L N F V P T V I M D P S K I E E S V R S M V M R Y G S R L W K L R V L

CAGGCTGAGCTGAAGATCAACATCCGCCTGACACCAACTGGGAATGCCATTCCTGTCCGCCTGTTTCTCACTAATGAATCTGGCTATTACTTGGACATTAGCCTGTACAAGGAGGTCACT

Q A E L K I N I R L T P T G N A I P V R L F L T N E S G Y Y L D I S L Y K E V T

GACCCAAGTTCAGGACAGATCATGTTCCAGTCGTACGGAGATAAGCAGGGTCCTCTGCATGGCATGCTGATCAACACTCCATATGTGACCAAAGACCTGCTGCAGGCCAAGCGTTTCCAG

D P S S G Q I M F Q S Y G D K Q G P L H G M L I N T P Y V T K D L L Q A K R F Q

GCTCAAACTCTGGGCACTACATACGTCTACGACTTCCCTGAGATGTTCAGACAGGCACTGTTTAAGCAGTGGGGTTCGGGGGACAAACACCCTAAAGACGTGCTGATGTGCACTGAGCTG

A Q T L G T T Y V Y D F P E M F R Q A L F K Q W G S G D K H P K D V L M C T E L

GTTCTGGACCCTCAAGGTCGACTTGTGCAGATGAATCGCCTGCCTGGAGACAATGATGTGGGAATGGTTGCCTTCAGGATGAAGATGAAGACTCCAGAGTACCCAGAAGGCAGAGATATC

V L D P Q G R L V Q M N R L P G D N D V G M V A F R M K M K T P E Y P E G R D I

ATCGTTATCTGCAATGACATCACTCACATGATCGGATCATTTGGCCCTCCAGAGGATGAGCTGTTCCTTAAGGCGTCAGAGTTGGCTCGGGCTGAGGGCATCCCGCGCATTTACATCGCA

I V I C N D I T H M I G S F G P P E D E L F L K A S E L A R A E G I P R I Y I A

GCCAACAGTGGCGCGCGCATCGGCCTCGCTGAAGAGATCAAACACATGTTCCAGGTGGCCTGGATTGACCCCTCTGACCCCTACAAGGGTTTCAAGTATCTTTACCTGACACCGCAGGAC

A N S G A R I G L A E E I K H M F Q V A W I D P S D P Y K G F K Y L Y L T P Q D

TACACACGTATCAGCTCCACCGGTGCTGTTCACTGTCGTCACGTAGAGGAAGGTGGAGAGTCCAGGTACATCATCACTGACATCATCGGGAAGGACGATGGTCTTGGGGTTGAGAACCTG

Y T R I S S T G A V H C R H V E E G G E S R Y I I T D I I G K D D G L G V E N L

CGAGGTTCTGGCACCATCGCTGGAGAATCCTCTCAGGCCTACGAGGAGATTATTACAATTAGTATGGTGACGTGTCGCGCTATTGGAATCGGAGCCTATCTGGTGCGTTTGGGACAGAGA

R G S G T I A G E S S Q A Y E E I I T I S M V T C R A I G I G A Y L V R L G Q R

GTGATACAAGTGGAAAACTCTCATATTATCCTGACTGGGGCAGGCGCTCTGAACAAGGTTTTGGGCAGAGAGGTCTATACCTCCAACAACCAGCTGGGAGGGATCCAGATCATGCACAAC

V I Q V E N S H I I L T G A G A L N K V L G R E V Y T S N N Q L G G I Q I M H N

AATGGAGTCACACACACCACTGTGCCAGATGACTTTGAGGGCGTCTTCACCATCCTCCGGTGGCTCTCCTATATGCCAAAGAACAAATACTCCCCCGTGCCTGTTATATCAACTACAGAT

N G V T H T T V P D D F E G V F T I L R W L S Y M P K N K Y S P V P V I S T T D

CCAGTGGACAGAGAGATAGAATATACTCCTACAAAAGCACCGTATGACCCCCGCTGGATGCTGGCTGGGAGACCTCATCCCACGGTGAGAGGTGCCTGGCAGAGCGGATTCTTCGATCAT

P V D R E I E Y T P T K A P Y D P R W M L A G R P H P T V R G A W Q S G F F D H

GGCTCTTTCATGGAGATCATGGAGTCTTGGGCTCAGACAGTGGTAGTGGGCAGAGCACGGTTAGGAGGAATTGCCCTCGGTGTCATCGCTGTTGAAACACGTACGGTTGAGTTCACAGTC

G S F M E I M E S W A Q T V V V G R A R L G G I A L G V I A V E T R T V E F T V

CCAGCAGATCCAGCAAACCTGGATTCAGAATCTAAGGTCCTGCAGCAGGCTGGCCAGGTGTGGTTTCCAGATTCAGCCTTTAAAACTGCTCAGGCCATTAGTGACTTCAACCGGGAGCGT

P A D P A N L D S E S K V L Q Q A G Q V W F P D S A F K T A Q A I S D F N R E R

CTGCCTCTCATGGTGTTTGCCAACTGGAGGGGCTTCTCTGGTGGAATGAAGGATATGTACGATCAGATATTGAAGTTTGGGGCCTACATCGTGGACGCCCTGCGTGGTTTTCATCAGCCG

L P L M V F A N W R G F S G G M K D M Y D Q I L K F G A Y I V D A L R G F H Q P

GTGCTGGTGTACATCCCACCTCAGGCTGAGCTAAGAGGAGGCTCATGGGTGGTGATAGACCCAACCATCAACCCGTTGTGTATGGAGCTCTATGCAGACAGGGAGAGCAGAGGTGGTGTG

V L V Y I P P Q A E L R G G S W V V I D P T I N P L C M E L Y A D R E S R G G V

CTGGAGGCTGAGGGTACAGTGGAGATCAAATTCAGGAGGAAGGACCTGCTGAAGACAATGAGAAGACTTGATTCAGTTTATGCTGGCCTGGTTGAGCAGCTTGCTTCCCTAGAGCTGTCT

L E A E G T V E I K F R R K D L L K T M R R L D S V Y A G L V E Q L A S L E L S

GACAAACAGTGCAAAGAGCTGGAGTCAAAGCTCAAAGCGAGGGAGGAATTCCTGTTGCCCATCTACCACCAGGTGGCAGTGCAGTTTGTAGAGCTCCATGACACCCCAGGAAGGATGCAG

D K Q C K E L E S K L K A R E E F L L P I Y H Q V A V Q F V E L H D T P G R M Q

GAGAAGGGTGTCATCACTGATATTTTGGATTGGAAAAATGTGCGGACCTTCTTCTACTGGCGTCTTCGTCGCCTCCTTTTGGAACAAGTGGTGAAGTGTGAGATTCTGCAGGCCAACAAG

E K G V I T D I L D W K N V R T F F Y W R L R R L L L E Q V V K C E I L Q A N K

GACCTCAGTGACGGGCACATGCAGTCCATGCTGCGACGCTGGTTTGTTGAAACAGAAGGAACAGTCAAGGCTTACCTGTGGGATAATAACCAAGCAGTAGTCGAGTGGCTTGAGAAGCAT

D L S D G H M Q S M L R R W F V E T E G T V K A Y L W D N N Q A V V E W L E K H

TTGTCTGAAGAGGATGGCGCTCGATCTGCGATCCGAGAGAACATCAAGTACCTGAAAAGGGAAAACACTTTGAAACACATCCGCAGCCTGGTGCAGGCCAACCCTGACGTAGCCATGGAC

L S E E D G A R S A I R E N I K Y L K R E N T L K H I R S L V Q A N P D V A M D

TGCATCATCCACATGAGCCATAATATCACTCCATCGCAGAGGGCCAAACTCTCACATTTACTCGCAACTATGGACAGCACCAGCACCAGT**TAA**

C I I H M S H N I T P S Q R A K L S H L L A T M D S T S T S *

Figure S2 Nucleotide and deduced amino acid sequences of *acc2* gene. The start codon (ATG) and the stop codon (TGA) are in bold.

```
Homo_sapiens ACC1            ..MWWSTLMSILRARSFWKWISTQTVRIIRAVRAHFGGIMDEPS...PLAQPLELNQHSR     55
Mus_musculus ACC1            .......................................MDEPS...PLAKTLELNQHSR     18
Bos_taurus ACC1              .......................................MDEPS...SLAKPLELNQHSR     18
Capra_hircus ACC1            ..MWWSTLMSILRASSFWKWISAQTIRIIRALRARFEGTMDEPS...SLAKPLELNQHSR     55
Danio_rerio ACC1             ......................................MAEQDS..TEKKLPAVAALHSH     20
Sparus_aurata ACC1           MFPWLTFVACLTAVLGLFFWSRKQLS.IVFAACSCRPAMAQQDG..AAKKSPAVAALHSH     57
Lates_calcarifer ACC1        ......................................MAQQDG.AAAKKNPAVAALHSH     21
Oncorhynchus_mykiss ACC1     ......................................MAQQDAPANKAAAPNLAVLHSN     22
Larimichthys_crocea ACC1     MFPWLTFAACLASVLGLFFWSRKQLTSIVFAACSCRPAMAQQDGAAAAKKNPAVAALHSH     60
Consensus                                                                           hs

Homo_sapiens ACC1            FIIGSVSEDNSEDEISNLVKLDLLEE.KEGSLSPAS..VGSDTLSDLGISSLQDGLALH.    111
Mus_musculus ACC1            FIIGSVSEDNSEDEISNLVKLDLEE..KEGSLSPAS..VSSDTLSDLGISGLQDGLAFH.     73
Bos_taurus ACC1              FIIGSVSEDNSEDEISNLVKLDLLEE.KEGSLSPAS..VSSDTLSDLGISSLQDGLALH.     74
Capra_hircus ACC1            FIIGSVSEDNSEDEISNLVKLDLLEE.KEGSLSPAS..VSSDTLSDLGISSLQDGLALH.    111
Danio_rerio ACC1             FIVGSVSEENSEDETAGKLDIQLEDN..PRSLSPSS..VSSDSTYEMGFDSLDGPLHN..     74
Sparus_aurata ACC1           FLVGSVSEENSEDEIQGKPDLQLEEKE.TRSLSPSS..GSSDSTFEMGFDHIDGPIHN..    112
Lates_calcarifer ACC1        FIVGSVSEENSEDEIQGKLDMQLEEKE.TRSLSPSS..GSSDSTYEMGFDHIDGPMHN..     76
Oncorhynchus_mykiss ACC1     FIIGSVSEENSEDEFLGKPDLTLGLEEKERSISPTSSLSSENSSYEMGFDNIDGPHMSCS     82
Larimichthys_crocea ACC1     FIVGSVSEENSEDEIQGKPDVQLEEKE.NRSLSPSS..ASSDSTCEMGFDHIDGPVQN..    115
Consensus                    f  gsvse nsede       l       s sp s          g

Homo_sapiens ACC1            IRSSMSGLHLVKQGRDRKKIDSQRDFTVASPAEFVTRFGGNKVIEKVLIANNGIAAVKCM    171
Mus_musculus ACC1            MRSSMSGLHLVKQGRDRKKIDSQRDFTVASPAEFVTRFGGNKVIEKVLIANNGIAAVKCM    133
Bos_taurus ACC1              MRSSMSGLHLVKQGRDRKKIDSQRDFTVASPAEFVTRFGGNKVIEKVLIANNGIAAVKCM    134
Capra_hircus ACC1            MRSSMSGLHLVKQGRDRKKIDSQRDFTVASPAEFVTRFGGNKVIEKVLIANNGIAAVKCM    171
Danio_rerio ACC1             MRPSMSGLHLVKQGRDRRRIDLQRDFTVASPAEFVTRFGGNKVIEKVLIANNGIAAVKCM    134
Sparus_aurata ACC1           LRPSMSGLHLVKQGRDRRRIDLQRDFTVASPAEFVTRFGGNKVIEKVLIANNGIAAVKCM    172
Lates_calcarifer ACC1        LRPSMSGLHLVKQGRDRRRIDLQRDFTVASPAEFVTRFGGNKVIEKVLIANNGIAAVKCM    136
Oncorhynchus_mykiss ACC1     HRPSMSGLHLVKQGRDRRRIDLQRDFTVASPAEFVTRFGGNKVIEKVLIANNGIAAVKCM    142
Larimichthys_crocea ACC1     LRPSMSGLHLVKQGRDRRRIDLQRDFTVASPAEFVTRFGGNKVIEKVLIANNGIAAVKCM    175
Consensus                     r smsglhlvkqgrdr  id qrdftvaspaefvtrfggnkviekvlianngiaavkcm

Homo_sapiens ACC1            RSIRRWSYEMFRNERAIRFVVMVTPEDLKANAEYIKMADHYVPVPGGPNNNNYANVELIL    231
Mus_musculus ACC1            RSIRRWSYEMFRNERAIRFVVMVTPEDLKANAEYIKMADHYVPVPGGPNNNNYANVELIL    193
Bos_taurus ACC1              RSIRRWSYEMFRNERAIRFVVMVTPEDLKANAEYIKMADHYVPVPGGPNNNNYANVELIL    194
Capra_hircus ACC1            RSIRRWSYEMFRNERAIRFVVMVTPEDLKANAEYIKMADHYVPVPGGPNNNNYANVELIL    231
Danio_rerio ACC1             RSIRRWAYEMFRNERAIRFVVMVTPEDLKANAEYIKMADHYVPVPGGTNNNNYANVELIL    194
Sparus_aurata ACC1           RSIRRWAYEMFRNERAIRFVVMVTPEDLKANAEYIKMADHYVPVPGGTNNNNYANVELIL    232
Lates_calcarifer ACC1        RSIRRWAYEMFRNERAIRFVVMVTPEDLKANAEYIKMADHYVPVPGGTNNNNYANVELIL    196
Oncorhynchus_mykiss ACC1     RSIRRWAYEMFRNERAIRFVVMVTPEDLKANAEYIKMADHYVPVPGGTNNNNYANVELIL    202
Larimichthys_crocea ACC1     RSIRRWAYEMFRNERAIRFVVMVTPEDLKANAEYIKMADHYVPVPGGTNNNNYANVELIL    235
Consensus                    rsirrw yemfrnerairfvvmvtpedlkanaeyikmadhyvpvpgg nnnnyanvelil

Homo_sapiens ACC1            DIAKRIPVQAVWAGWGHASENPKLPELLLKNGIAFMGPPSQAMWALGDKIASSIVAQTAG    291
Mus_musculus ACC1            DIAKRIPVQAVWAGWGHASENPKLPELLLKNGIAFMGPPSQAMWALGDKIASSIVAQTAG    253
Bos_taurus ACC1              DIAKRIPVQAVWAGWGHASENPKLPELLLKNGIAFMGPPSQAMWALGDKIASSIVAQTAG    254
Capra_hircus ACC1            DIARRIPVQAVWAGWGHASENPKLPELLLKNGIAFMGPPSQAMWALGDKIASSIVAQTAG    291
Danio_rerio ACC1             DIAKRIPVQAVWAGWGHASENPKLPELLHKNGIAFMGPPSQAMWALGDKIASSIVAQTAG    254
Sparus_aurata ACC1           DIAKRIPVQAVWAGWGHASENPKLPELLHKNGIAFMGPPSQAMWSLGDKIASSIVAQTAG    292
Lates_calcarifer ACC1        DIAKRIPVQAVWAGWGHASENPKLPELLQKHGIAFMGPPSQAMWALGDKIASSIVAQTAG    256
Oncorhynchus_mykiss ACC1     DIAKRIPVQAVWAGWGHASENPKLPELLMKNGIAFMGPPSQAMWALGDKIASSIVAQTAG    262
Larimichthys_crocea ACC1     DIAKRIPVQAVWAGWGHASENPKLPELLHKHGIAFMGPPSQAMWALGDKIASSIVAQTAG    295
Consensus                    dia ripvqavwagwghasenpklpell k giafmgppsqamw lgdkiassivaqtag

Homo_sapiens ACC1            IPTLPWSGSGLRVDWQENDFSKRILNVPQELYEKGYVKDVDDGLQAAEEVGYPVMIKASE    351
Mus_musculus ACC1            IPTLPWSGSGLRVDWQENDFSKRILNVPQDLYEKGYVKDVDDGLKAAEEVGYPVMIKASE    313
Bos_taurus ACC1              IPTLPWSGSGLCVDWHENDFSKRILNVPQELYEKGYVKDVDDGLKAAEEVGYPVMIKASE    314
Capra_hircus ACC1            IPTLPWSGSGLCVDWHENDFSKRILNVPQELYEKGYVKDVDDGLKAAEEVGYPVMIKASE    351
Danio_rerio ACC1             IPTLPWSGTGLTVEWTENDQKKGIINVPTELYEQGCVHDVEAGLKAAEQVGFPVMVKASE    314
Sparus_aurata ACC1           IPTLPWSGTALTVEWTENDQRKKIINVPTDVYELGCIQDVEDGLKAAEKVGYPVMVKASE    352
Lates_calcarifer ACC1        IPTLPWSGTGLTVEWTENNQKKRIINVPHDVYELGCIQDVEDGLKAAEKVGYPLMVKASE    316
Oncorhynchus_mykiss ACC1     IPTLPWSGAGLTVDWTESDQKKRIINVPPDLYELGCIHDVEAGLKAAEVVGYPVMVKASE    322
Larimichthys_crocea ACC1     IPTLPWSGTGLSVEWTENNQKKKVINVPTDVYELGCIQDVEDGLKAAEKIGYPVMVKASE    355
Consensus                    iptlpwsg  l v w e     k   nvp    ye g    dv   gl aae   g p m kase

Homo_sapiens ACC1            GGGGKGIRKVNNADDFPNLFRQVQAEVPGSPIFVMRLAKQSRHLEVQILADQYGNAISLF    411
Mus_musculus ACC1            GGGGKGIRKVNNADDFPNLFRQVQAEVPGSPIFVMRLAKQSRHLEVQILADQYGNAISLF    373
Bos_taurus ACC1              GGGGKGIRKVNNADDFPNLFRQVQAEVPGSPIFVMRLAKQSRHLEVQILADQYGNAISLF    374
Capra_hircus ACC1            GGGGKGIRKVNNADDFPNLFRQVQAEVPGSPIFVMRLAKQSRHLEVQILADQYGNAISLF    411
Danio_rerio ACC1             GGGGKGIRKVNCAEDFPNLYRQVQAEVPGSPIFVMQLAKHARHLEVQILADQYGNAISLF    374
Sparus_aurata ACC1           GGGGKGIRKVNCADDFPNLFRQVQAEVPGSPIFVMQLAKHARHLEVQILADQYGNAISLF    412
Lates_calcarifer ACC1        GGGGKGIRKVNSADDFPNLFRQVQAEVPGSPIFIMELAKHARHLEVQILADQYGSAISLF    376
Oncorhynchus_mykiss ACC1     GGGGKGIRKVNCADDFPNLFRQVQTEVPGSPIFVMQLAKHARHLEVQLLADQYGNAISLF    382
Larimichthys_crocea ACC1     GGGGKGIRKVNCVDDFPNLFRQVQAEVPGSPVFVMQLAKHARHLEVQILADQYGNAISLF    415
Consensus                    ggggkgirkvn   dfpnl rqvq evpgsp f m lak  rhlevq ladqyg aislf

Homo_sapiens ACC1            GRDCSVQRRHQKIIEEAPATIATPAVFEHMEQCAVKLAKMVGYVSAGTVEYLYSQDGSFY    471
Mus_musculus ACC1            GRDCSVQRRHQKIIEEAPAAIATPAVFEHMEQCAVKLAKMVGYVSAGTVEYLYSQDGSFY    433
Bos_taurus ACC1              GRDCSVQRRHQKIIEEAPAAIATPAVFEHMEQCAVKLARMVGYVSAGTVEYLYSQDGSFY    434
Capra_hircus ACC1            GRDCSVQRRHQKIIEEAPAAIATPAVFEHMEQCAVKLARMVGYVSAGTVEYLYSQDGSFY    471
Danio_rerio ACC1             GRDCSVQRRHQKIIEEAPATIATSDVFEDMEKCAVKLAKMVGYVSAGTVEYLYSQDSSFY    434
Sparus_aurata ACC1           GRDCSVQRRHQKIIEEAPATIATSDVFEDMERCAVKLAKMVGYVSAGTVEYLYSQDGSFY    472
Lates_calcarifer ACC1        GRDCSVQRRHQKIIEEAPATIATSDVFEDMERCAVKLAKMVGYVSAGTVEYLYSQEGSFY    436
Oncorhynchus_mykiss ACC1     GRDCSVQRRHQKIIEEAPATISTSAVFEDMERCAVKLAKMVGYVSAGTVEYLYSQDGSFY    442
Larimichthys_crocea ACC1     GRDCSVQRRHQKIIEEAPATIATSDVFEDMERCAVKLAKMVGYVSAGTVEYLYSQDGSFY    475
Consensus                    grdcsvqrrhqkiieeapa i t  vfe me cavkla mvgyvsagtveylysq  sfy
```

```
Homo_sapiens ACC1              FLELNPRLQVEHPCTEMVADVNLPAAQLQIAMGIPLYRIKDIRMMYGVSPWGDSPIDFED   531
Mus_musculus ACC1              FLELNPRLQVEHPCTEMVADVNLPAAQLQIAMGIPLFRIKDIRMMYGVSPWGDAPIDFEN   493
Bos_taurus ACC1                FLELNPRLQVEHPCTEMVADVNLPAAQLQIAMGIPLYRIKDIRMMYGVSPWGDAPIDFEN   494
Capra_hircus ACC1              FLELNPRLQVEHPCTEMVADVNLPAAQLQIAMGIPLYRIKDIRMMYGVSPWGDAPIDFEN   531
Danio_rerio ACC1               FLELNPRLQVEHPCTEMVADVNLPAAQLQIAMGIPLHRIKDIRVLYGMQPWGDSPIDFDG   494
Sparus_aurata ACC1             FLELNPRLQVEHPCTEMVADVNLPAAQLQIAMGIPLQRIKDIRMLYGVQPWGDSPIDFEG   532
Lates_calcarifer ACC1          FLELNPRLQVEHPCTEMVADVNLPAAQLQIAMGIPLHRIKDIRMLYGVQPWGDSPIDFEG   496
Oncorhynchus_mykiss ACC1       FLELNPRLQVEHPCTEMVADVNLPAAQLQIAMGIPLHRIKDIRMMYGVQPWGDSMIDFEG   502
Larimichthys_crocea ACC1       FLELNPRLQVEHPCTEMVADVNLPAAQLQIAMGIPLQRIKDIRMLYGVQPWGDSPIDFEG   535
Consensus                      flelnprlqvehpctemvadvnlpaaqlqiamgipl rikdir  yg  pwgd  idf

Homo_sapiens ACC1              SAHVPCPRGHVIAARITSENPDEGFKPSSGTVQELNFRSNKNVWGYFSVAAAGGLHEFAD   591
Mus_musculus ACC1              SAHVPCPRGHVIAARITSENPDEGFKPSSGTVQELNFRSNKNVWGYFSVAAAGGLHEFAD   553
Bos_taurus ACC1                SAHVPCPRGHVIAARITSENPDEGFKPSSGTVQELNFRSNKNVWGYFSVAAAGGLHEFAD   554
Capra_hircus ACC1              SAHVPCPRGHVIAARITSENPDEGFKPSSGTVQELNFRSNKNVWGYFSVAAAGGLHEFAD   591
Danio_rerio ACC1               LSTTPCPRGHVIAARITSENPDEGFKPSSGTVQELNFRSNKNVWGYFSVAAAGGLHEFAD   554
Sparus_aurata ACC1             LSTAPSPRGHVIAARITSENPDEGFKPSSGTVQELNFRSNKNVWGYFSVAAAGGLHEFAD   592
Lates_calcarifer ACC1          LSTAPSPRGHVIAARITSENPDEGFKPSSGTVQELNFRSNKNVWGYFSVAAAGGLHEFAD   556
Oncorhynchus_mykiss ACC1       LSTAPSPRGHVIAARITSENPDEGFKPSSGTVQELNFRSNKNVWGYFSVAAAGGLHEFAD   562
Larimichthys_crocea ACC1       LSTAPCPRGHVIAARITSENPDEGFKPSSGTVQELNFRSNKNVWGYFSVAAAGGLHEFAD   595
Consensus                          p prghviaaritsenpdegfkpssgtvqelnfrsnknvwgyfsvaaagglhefad

Homo_sapiens ACC1              SQFGHCFSWGENREEAISNMVVALKELSIRGDFRTTVEYLIKLLETESFQMNRIDTGWLD   651
Mus_musculus ACC1              SQFGHCFSWGENREEAISNMVVALKELSIRGDFRTTVEYLIKLLETESFQLNRIDTGWLD   613
Bos_taurus ACC1                SQFGHCFSWGENREEAISNMVVALKELSIRGDFRTTVEYLIKLLETESFQLNRIGTGWLD   614
Capra_hircus ACC1              SQFGHCFSWGENREEAISNMVVALKELSIRGDFRTTVEYLIKLLETESFQLNRIDTGWLD   651
Danio_rerio ACC1               SQFGHCFSWGENREEAISNMVVALKELSIRGDFRTTVEYLIKLLETESFQHNSIDTGWLD   614
Sparus_aurata ACC1             SQFGHCFSWGENREEAISNMVVALKELSIRGDFRTTVEYLIKLLETESFQHNSIDTGWLD   652
Lates_calcarifer ACC1          SQFGHCFSWGENREEAISNMVVALKELSIRGDFRTTVEYLIKLLETESFQHNSIDTGWLD   616
Oncorhynchus_mykiss ACC1       SQFGHCFSWGENREEAISNMVVALKELSIRGDFRTTVEYLIKLLETESFQHNTIDTGWLD   622
Larimichthys_crocea ACC1       SQFGHCFSWGENREEAISNMVVALKELSIRGDFRTTVEYLIKLLETESFQHNSIDTGWLD   655
Consensus                      sqfghcfswgenreeaisnmvvalkelsirgdfrttveylikllletesfq n i tgwld

Homo_sapiens ACC1              RLIAEKVQAERPDTMLGVVCGALHVADVSLRNSVSNFLHSLERGQVLPAHTLLNTVDVEL   711
Mus_musculus ACC1              RLIAEKVQAERPDTMLGVVCGALHVADVSLRNSISNFLHSLERGQVLPAHTLLNTVDVEL   673
Bos_taurus ACC1                RLIAEKVQAERPDTMLGVVCGALHVADVSLRNSISNFLHSLERGQVLTAHTLLNTVDVEL   674
Capra_hircus ACC1              RLIAEKVQAERPDTMLGVVCGALHVADVSLRNSISNFLHSLERGQVLSAHTLLNTVDVEL   711
Danio_rerio ACC1               RLISEKMQAERPDTMLGVVSGALHVADVNFRNSVSNFLHSLERGQVLPAHTLLNTVDVEL   674
Sparus_aurata ACC1             RLISEKMQAERPDTMLGIVSGALHVADVNLRNSVSNFLHSLERGQVLPAHTLLNTVDVEL   712
Lates_calcarifer ACC1          RLISEKMQAERPDTMLGIVSGALHVADVNLRNSVSNFLHSLERGQVLPAHTLLNTVDVEL   676
Oncorhynchus_mykiss ACC1       RLISEKMQAERPDTMLGIVSGALHVADVNLRNSVSNFLHSLERGQVLPAHTLLNTVDVEL   682
Larimichthys_crocea ACC1       RLISEKMQAERPDTMLGIVSGALHVADVNLRNSVSNFLHSLERGQVLPAHTLLNTVDVEL   715
Consensus                      rli ek qaerpdtmlg v galhvadv  rns snflhslergqvl ahtllntvdvel

Homo_sapiens ACC1              IYEGVKYVLKVTRQSPNSYVVIMNGSCVEVDVHRLSDGGLLLSYDGSSYTTYMKEEVDRY   771
Mus_musculus ACC1              IYEGIKYVLKVTRQSPNSYVVIMNGSCVEVDVHRLSDGGLLLSYDGSSYTTYMKEEVDRY   733
Bos_taurus ACC1                IYEGVKYVLKVTRQSPNSYVVIMNGSCVEVDVHRLSDGGLLLSYDVSSYTTYMKEEVDRY   734
Capra_hircus ACC1              IYEGIKYVLKVTRQSPNSYVVIMNGSCVEVDVHRLSDGGLLLSYDGSSYTTYMKEEVDRY   771
Danio_rerio ACC1               IYEGTKYVLKVTRQSPNSYVVIMNCSSVEVDVHRLSDGGMLLSYDGSSYTTYMKEEVDRY   734
Sparus_aurata ACC1             IYEGIKYVLTVTRQSPNSYVVIMNNSSAEVDVHRLSDGGLLLSYDGSSYTTYMKEEVDRY   772
Lates_calcarifer ACC1          IYEGTKYVLTVTRQSPNSYVVIMNNSSAEVDVHRLSDGGLLLSYDGSSYTTYMKEEVDRY   736
Oncorhynchus_mykiss ACC1       IYEGTKYCLKVTRQSPNSYVVIMNSTAAEVDVHRLSDGGLLLSYDGSSYTTYMKEEVDRY   742
Larimichthys_crocea ACC1       IYEGTKYVLTVTRQSPNSYVVIMNNSSAEVDVHRLSDGGLLLSYDGSSYTTYMKEEVDRY   775
Consensus                      iyeg ky l vtrqspnsyvvimn    evdvhrlsdgg llsyd ssyttymkeevdry

Homo_sapiens ACC1              RITIGNKTCVFEKENDPSVMRSPSAGKLIQYIVEDGGHVFAGQCYAEIEVMKMVMTLTAV   831
Mus_musculus ACC1              RITIGNKTCVFEKENDPSVMRSPSAGKLIQYIVEDGGHVFAGQCYAEIEVMKMVMTLTAV   793
Bos_taurus ACC1                RITIGNKTCVFEKENDPSVLRSPSAGKLIQYIVEDGGHVFAGQCYAEIEVMKMVMTLTAA   794
Capra_hircus ACC1              RITIGNKTCVFEKENDPSVLRSPSAGKLIQYIVEDGGHVFAGQCYAEIEVMKMVMTLTAA   831
Danio_rerio ACC1               RIIIGNKTCVFEKENDPSVLRSPSAGKLIQYTVEDGGHVFAGQCYAEIEVMKMVMTLTAS   794
Sparus_aurata ACC1             RITIGNKTCVFEKENDPSLLRSPSAGKIIQYTVEDGGHVFSGQCYAEIEVMKMVMTLTAA   832
Lates_calcarifer ACC1          RITIGNKTCVFERENDPSLLRSPSAGKLIQYTVEDGGHVFSGQCYAEIEVMKMVMTLTAA   796
Oncorhynchus_mykiss ACC1       RITIGNKTCVFERENDPSLLRSPSAGKLIQYTVEDGGHVFSGQCYAEIEVMKMVMTLTAL   802
Larimichthys_crocea ACC1       RITIGNKTCVFERENDPSLLRSPSAGKLIQYTVEDGGHVFSGQCYAEIEVMKMVMTLTAA   835
Consensus                      ri ignktcvfe endps  rspsagk iqy vedgghvf gqcyaeievmkmvmtlta

Homo_sapiens ACC1              ESGCIHYVKRPGAALDPGCVLAKMQLDNPSKVQQAELHTGSLPRIQSTALRGEKLHRVFH   891
Mus_musculus ACC1              ESGCIHYVKRPGAALDPGCVIAKMQLDNPSKVQQAELHTGSLPQIQSTALRGEKLHRVFH   853
Bos_taurus ACC1                ESGCIHYVKRPGAALDPGCVIAKMQLDNPSKVQQAELHTGSLPRIQSTALRGEKLHRVFH   854
Capra_hircus ACC1              ESGCIHYVKRPGAALDPGCVIAKMQLDNPSKVQQAELHTGSLPRIQSTALRGEKLHRVFH   891
Danio_rerio ACC1               ESGCIHYVKRAGAVLEPGCIIGKLQLDDPSRVQQAELYTGTLPSIQSVALRGEKLHRVFH   854
Sparus_aurata ACC1             ESGCIHYVKRAGAALEPGCVIAKLQLDDPSRVQQAELHTGALPSIQAVALRGEKLHRVFH   892
Lates_calcarifer ACC1          ESGCIHYVKRAGAALEPGCVIAKLQLDDPSRVQQAELHTGALPSIQAVALRGEKLHRVFH   856
Oncorhynchus_mykiss ACC1       VSGCIHYVKRPGAVLEPGCVIAKLQLDDPGRVQQAELHHGALPVIQAAALRGEKLHRVFH   862
Larimichthys_crocea ACC1       ESGCIHYVKRAGAALDPGCVIAKLQLDDPSRVQQAELHTGALPSIQAVALRGEKLHRVFH   895
Consensus                       sgcihyvkr ga l pgc   k qld p  vqqael  g lp iq  alrgeklhrvfh

Homo_sapiens ACC1              YVLDNLVNVMNGYCLPDPFFSSKVKDWVERLMKTLRDPSLPLLELQDIMTSVSGRIPPNV   951
Mus_musculus ACC1              YVLDNLVNVMNGYCLPDPFFSSRVKDWVERLMKTLRDPSLPLLELQDIMTSVSGRIPLNV   913
Bos_taurus ACC1                YVLDNLVNVMNGYCLPDPFFSSRVKDWVERLMKTLRDPSLPLLELQDIMTSVSGRIPPNV   914
Capra_hircus ACC1              YVLDNLVNVMNGYCLPDPFFSSRVKDWVERLMKTLRDPSLPLLELQDIMTSVSGRIPPNV   951
Danio_rerio ACC1               STLGHLVHIMNGYCLPEPFFSAKLKEWVERLMKTMRDPSLPLLELQDIMTSVSGRIPPAV   914
Sparus_aurata ACC1             NTLDHLVHIMNGYCLPEPFFSAKLKEWVERLMKTMRDPSLPLLELQDIMTSVSGRIPPAV   952
Lates_calcarifer ACC1          NTLDHLVHIMNGYCLPEPFFSAKLKEWVERLMKTMRDPSLPLLELQDIMTSVSGRIPPAV   916
Oncorhynchus_mykiss ACC1       STLDHLVHIMSGFCLPEPFFSGKLKEWVERLMKTFRDPSLPLLELQDIMTSVSGRIPLAV   922
Larimichthys_crocea ACC1       NTLDHLVHIMNGYCLPEPFFSAKLKEWVERLMKTMRDPSLPLLELQDIMTSVSGRIPPAV   955
Consensus                       l  lv  m g clp pffs   k wverlmkt rdpslpllelqdimtsvsgrip  v
```

```
Homo_sapiens ACC1            EKSIKKEMAQYASNITSVLCQFPSQQIANILDSHAATLNRKSEREVFFMNTQSIVQLVQR  1011
Mus_musculus ACC1            EKSIKKEMAQYASNITSVLCQFPSQQIANILDSHAATLNRKSEREVFFMNTQSIVQLVQR   973
Bos_taurus ACC1              EKSIKKEMAQYASNITSVLCQFPSQQIANILDSHAATLNRKSEREVFFMNTQSIVQLVQR   974
Capra_hircus ACC1            EKSIKKEMAQYASNITSVLCQFPSQQIANILDSHAATLNRKSEREVFFMNTQSIVQLVQR  1011
Danio_rerio ACC1             EKAIKKEMAQYASNITSVLCQFPSQQIANILDSHAATLNRKSEREVFFMNTQSIVQLVQK   974
Sparus_aurata ACC1           EKAIKKEMAQYASNITSVLCQFPSQQIANILDSHAATLNKKSEREVFFMNTQSIVQLVQK  1012
Lates_calcarifer ACC1        EKAIKKEMAQYASNITSVLCQFPSQQIANILDSHAATLNKKSEREVFFMNTQSIVQLVQK   976
Oncorhynchus_mykiss ACC1     EKCIKKEMAQYASNITSVLCQFPSQQIANILDSHAATLNRKSEREVFFMNTQSIVQLVQK   982
Larimichthys_crocea ACC1     EKAIKKEMAQYASNITSVLCQFPSQQIANILDSHAATLNKKSEREVFFMNTQSIVQLVQK  1015
Consensus                    ek ikkemaqyasnitsvlcqfpsqqianildshaatln kserevffmntqsivqlvq

Homo_sapiens ACC1            YRSGIRGHMKAVVMDLLRQYLRVETQFQNGHYDKCVFALREENKSDMNTVLNYIFSHAQV  1071
Mus_musculus ACC1            YRSGIRGHMKAVVMDLLRQYLRVETQFQNGHYDKCVFALREENKSDMNTVLNYIFSHAQV  1033
Bos_taurus ACC1              YRSGIRGHMKAVVMDLLRQYLRVETQFQNGHYDKCVFALREENKSDMNTVLNYIFSHAQV  1034
Capra_hircus ACC1            YRSGIRGHMKAVVMDLLRQYLRVETQFQNGHYDKCVFALREENKSDMNTVLNYIFSHAQV  1071
Danio_rerio ACC1             YRSGIRGHMKAVVMDLLRQYLRVEVQFQHGHYDKCVFALREENKVDMANVLNYIFSHAQV  1034
Sparus_aurata ACC1           YRSGIRGHMKAVVMDLLRQYLKVEIQFQNGHYDKCVFTLREENKGDMANVLNYIFSHAQV  1072
Lates_calcarifer ACC1        YRSGIRGHMKAVVMDLLRQYLKVEIQFQNGHYDKCVFALREENKGDMANVLNYIFSHAQV  1036
Oncorhynchus_mykiss ACC1     YRSGIRGHMKAVVMDLLRQYLKVEVQFQHGHYEKCVFTLREENKSDMANVLNYIFSHAQV  1042
Larimichthys_crocea ACC1     YRSGIRGHMKAVVMDLLRQYLKVEIQFQNGHYDKCVFALREENKGDMSNVLNYIFSHAQV  1075
Consensus                    yrsgirghmkavvmdllrqyl ve qfq ghy kcvf lreenk dm  vlnyifshaqv

Homo_sapiens ACC1            TKKNLLVTMLIDQLCGRDPTLTDELLNILTELTQLSKTTNAKVALRARQVLIASHLPSYE  1131
Mus_musculus ACC1            TKKNLLVTMLIDQLCGRDPTLTDELLNILTELTQLSKTTNAKVALRARQVLIASHLPSYE  1093
Bos_taurus ACC1              TRKNLLVTMLIDQLCGRGPTLTDELLNILTELTQLSKTTNAKVALRARQVLIASHLPSYE  1094
Capra_hircus ACC1            TKKNLLVTMLIDQLCGRDPTLTDELLNILTELTQLSKTTNAKVALRARQVLIASHLPSYE  1131
Danio_rerio ACC1             TKKNCLVTMLIDQLCGRDPTLTDELMAILTELTQLSKTTNAKVALRARQVLIASHLPSYE  1094
Sparus_aurata ACC1           TKKNLLVTMLIDQLCGRDPTLTDELMAILTELTQLSKTTNAKVALRARQVLIASHLPSYE  1132
Lates_calcarifer ACC1        TKKNLLVTMLIDQLCGRDPTLTDELMAILTELTQLSKTTNAKVALRARQVLIASHLPSYE  1096
Oncorhynchus_mykiss ACC1     TKKNLLVTMLIDQLCGRDPTLTDELMAILTELTQLSKTTNAKVALRARQVLIASHLPSYE  1102
Larimichthys_crocea ACC1     TKKNLLVTMLIDQLCGRDPTLTDELMAILTELTQLSKTTNAKVALRARQVLIASHLPSYE  1135
Consensus                    t kn lvtmlidqlcgr ptltdel  ilteltqlskttnakvalrarqvliashlpsye

Homo_sapiens ACC1            LRHNQVESIFLSAIDMYGHQFCIENLQKLILSETSIFDVLPNFFYHSNQVVRMAALEVYV  1191
Mus_musculus ACC1            LRHNQVESIFLSAIDMYGHQFCIENLQKLILSETSIFDVLPNFFYHSNQVVRMAALEVYV  1153
Bos_taurus ACC1              LRLNQVESIFLSAIDMYGHQFCIENLQKLILSETSIFDVLPNFFYHSNQVVRMAALEVYV  1154
Capra_hircus ACC1            LRHNQVESIFLSAIDMYGHQFCIENLQKLILSETSIFDVLPNFFYHSNQVVRMAALEVYV  1191
Danio_rerio ACC1             LRHNQVESIFLSAIDMYGHQFCIENLQKLILSETSIFDVLPNFFYHSNQVVRMAALEVYV  1154
Sparus_aurata ACC1           LRHNQVESIFLSAIDMYGHQFCIENLQKLILSETSIFDVLPNFFYHSNQVVRMAALEVYV  1192
Lates_calcarifer ACC1        LRHNQVESIFLSAIDMYGHQFCIENLQKLILSETSIFDVLPNFFYHSNQVVRMAALEVYV  1156
Oncorhynchus_mykiss ACC1     LRHNQVESIFLSAIDMYGHQFCIENLQKLILSETSIFDVLPNFFYHSNQVVRMAALEVYV  1162
Larimichthys_crocea ACC1     LRHNQVESIFLSAIDMYGHQFCIENLQKLILSETSIFDVLPNFFYHSNQVVRMAALEVYV  1195
Consensus                    lr nqvesiflsaidmyghqfcienlqklilsetsifdvlpnffyhsnqvvrmaalevyv

Homo_sapiens ACC1            RRAYIAYELNSVQHRQLKDNTCVVEFQFMLPTSHPNRGNIPTLN................  1235
Mus_musculus ACC1            RRAYIAYELNSVQHRQLKDNTCVVEFQFMLPTSHPNRGNIPTLN................  1197
Bos_taurus ACC1              RRAYIAYELNSVQHRQLKDNTCVVEFQFMLPTSHPNRGNIPTLN................  1198
Capra_hircus ACC1            RRAYIAYELNSVQHRQLKDNTCVVEFQFMLPTSHPNRGNIPTLN................  1235
Danio_rerio ACC1             RRAYIAYELNSVQHRQLKDNTCIVEFQFMLPTSHPNRGNIPTLNRKLSAP..........  1204
Sparus_aurata ACC1           RRAYIAYELNSVQHRQLRDNTCVVEFQFMLPTSHPNRGNIPTLN................  1236
Lates_calcarifer ACC1        RRAYIAYELNSVQHRQLKDNTCVVEFQFMLPTSHPNRGNIPTLN................  1200
Oncorhynchus_mykiss ACC1     RRAYIAYELNSVQHRQLKDNTCIVEFQFMLPTSHPNRGNIPTLN................  1206
Larimichthys_crocea ACC1     RRAYIAYELNSVQHRQLRDNTCIVEFQFMLPTSHPNRGNIPTLNRRMSTPFPDIVKATDT  1255
Consensus                    rrayiayelnsvqhrql dntc vefqfmlptshpnrgniptln

Homo_sapiens ACC1            ..................................RMSFSSNLNHYGMTHVASVSDVLLDN  1261
Mus_musculus ACC1            ..................................RMSFASNLNHYGMTHVASVSDVLLDN  1223
Bos_taurus ACC1              ..................................RMSFSSNLNHYGMTHVASVSDVLLDN  1224
Capra_hircus ACC1            ..................................RMSFSSNLNHYGMTHVASVSDVLLDN  1261
Danio_rerio ACC1             ......LQDYKPPANTANTSGATEAASDSAESEDRMSFSSNLNHYGMVHVASVSDVLLDT  1258
Sparus_aurata ACC1           ..................................RMSFSSNLNHYGMVHVASVSDVLLDT  1262
Lates_calcarifer ACC1        ..................................RMSFSSNLNHYGMVHVASVSDVLLDT  1226
Oncorhynchus_mykiss ACC1     ..................................RMSFSSNLNHYGMVHMGSVSDVLLDT  1232
Larimichthys_crocea ACC1     KFQETKPQDLNAQASKSKDDNAEKRNGSDSETVDRMSFSSNLNHYGMVHVASVSDVLLDT  1315
Consensus                                                      rmsf snlnhygm h  svsdvlld

Homo_sapiens ACC1            SFTPPCQRMGGMVSFRTFEDFVRIFDEVMGCFSDSPPQSPTFPEAGHTSLYDED..KVPR  1319
Mus_musculus ACC1            AFTPPCQRMGGMVSFRTFEDFVRIFDEIMGCFCDSPPQSPTFPESGHTSLYDED..KVPR  1281
Bos_taurus ACC1              AFTPPCQRMGGMVSFRTFEDFVRIFDEVMGCFCDSPPQSPTFPEAGHTSLYDED..KVPR  1282
Capra_hircus ACC1            AFTPPCQRMGGMVSFRTFEDFVRIFDEVMGCFCDSPPQSPTFPEAGHTSLYDED..KVPR  1319
Danio_rerio ACC1             SFTPPCQRMGAMVSFRSFQEFTRNIKDVLSCFSDSPPSTPTFPEGGNPVLYREEDSKSIQ  1318
Sparus_aurata ACC1           SFTPPCQRMGAMVSFRSFLEFTRNITDVLSCFSDSPPPSPTFPEGGNPVLYGEEDNKSVQ  1322
Lates_calcarifer ACC1        SFTPPCQRMGAMVAFRSFPEFTRNITDVLSCFSDSPPPSPTFPEGGNPVLYGEEDNKSAQ  1286
Oncorhynchus_mykiss ACC1     SFTPPCQRMGAMVSFRSFQEFTRDIKDVMSCFSDSPPHSPTFPEGGNPVLYGEEDTKSIQ  1292
Larimichthys_crocea ACC1     SFTPPCQRMGAMVAFRSFQEFTRNIADVLSCFSDSPPPSPTFPEGGNPVLYGEEDNKSVQ  1375
Consensus                     ftppcqrmg mv fr f  f r       cf dspp  ptfpe g   ly e   k

Homo_sapiens ACC1            DEPIHILNVAIKTDCDIEDDRLAAMFREFTQQNKATLVDHGIRRLTFLVAQKDFRKQVNY  1379
Mus_musculus ACC1            DEPIHILNVAIKTDGDIEDDRLAAMFREFTQQNKATLVEHGIRRLTFLVAQKDFRKQVNC  1341
Bos_taurus ACC1              DEPIHILNVAIKTDCDIEDDSLAAMFREFTQQNKATLVEHGIRRLTFLVAQKDFRKQVNY  1342
Capra_hircus ACC1            DEPIHILNVAIKTDCDIEDDSLAAMFREFTQQNKATLVEHGIRRLTFLVAQKDFRKQVNY  1379
Danio_rerio ACC1             DEPIHILNVAIKTDSDIDDDGLAAMFREFTQSKKSLLFDHGIRRLTFLVAQKDFRKQINC  1378
Sparus_aurata ACC1           DEPIHILNVAIKTDSDIEDDGLAANFREFTQSNKSLLFEHGIRRLTFLVAQKDFRKQVNC  1382
Lates_calcarifer ACC1        DEPIHILNVAIKTDSDIDDDGLAAMFRDFTQSKKSLLFEHGIRRLTFLVAQKDFRKQVNC  1346
Oncorhynchus_mykiss ACC1     DEPVHILNVAIKTDGDIDDDGLAAMFREFTQSEKSLLFEHGIRRLTFLVAQKDFRKAVNY  1352
Larimichthys_crocea ACC1     DEPIHILNVAIKTDSDIDDDGLAANFREFTQSKKSLLFEHGIRRLTFLVAQKDFRKQVNC  1435
Consensus                    dep hilnvaiktd di dd laa fr ftq  k  l  hgirrltflvaqkdfrk  n
```

```
Homo_sapiens ACC1             EVDRRFHREFPKFFTFRARDKFEEDRIYRHLEPALAFQLELNRMRNFDLTAIPCANHKMH   1439
Mus_musculus ACC1             EVDQRFHREFPKFFTFRARDKFEEDRIYRHLEPALAFQLELNRMRNFDLTAIPCANHKMH   1401
Bos_taurus ACC1               EVDQRFHREFPKFFTFRARDKFEEDRIYRHLEPALAFQLELNRMRNFDLTAIPCANHKMH   1402
Capra_hircus ACC1             EVDQRFHREFPKFFTFRARDKFEEDRIYRHLEPALAFQLELNRMRNFDLTAIPCANHKMH   1439
Danio_rerio ACC1              EVDQRFHREFPKFFTFRARDKFEEDRIYRHLEPALAFQLELNRMRNFALTAIPCANHKMH   1438
Sparus_aurata ACC1            EVDQRFHREFPKFFTFRARDKFEEDRIYRHLEPALAFQLELNRMRNFALTAIPCANHKMH   1442
Lates_calcarifer ACC1         EVDQRFHREFPKFFTFRARDKFEEDRIYRHLEPALAFQLELNRMRNFALTAIPCANHKMH   1406
Oncorhynchus_mykiss ACC1      EVDQRFHREFPKFFTFRSRDKFEEDRIYRHLEPALAFQLELNRMRNFALTAIPCANHKMH   1412
Larimichthys_crocea ACC1      EVDQRFHREFPKFFTFRARDKFEEDRIYRHLEPALAFQLELNRMRNFALTAIPCANHKMH   1495
Consensus                     evd rfhrefpkfftfr rdkfeedriyrhlepalafqlelnrmrnf ltaipcanhkmh

Homo_sapiens ACC1             LYLGAAKVEVGTEVTDYRFFVRAIIRHSDLVTKEASFEYLQNEGERLLLEAMDELEVAFN   1499
Mus_musculus ACC1             LYLGAAKVEVGTEVTDYRFFVRAIIRHSDLVTKEASFEYLQNEGERLLLEAMDELEVAFN   1461
Bos_taurus ACC1               LYLGAAKVEVGTEVTDYRFFVRAIIRHSDLVTKEASFEYLQNEGERLLLEAMDELEVAFN   1462
Capra_hircus ACC1             LYLGAAKVEVGTEVTDYRFFVRAIIRHSDLVTKEASFEYLQNEGERLLLEAMDELEVAFN   1499
Danio_rerio ACC1              LYLGAARVEVGTEVTDYRFFVRAIIRHSDLVTKEASFEYLHNEAERLLLEAMDELEVAFN   1498
Sparus_aurata ACC1            LYLGAARVEVGTEVTDYRFFVRAIIRHSDLVTKEASFEYLHNEAERLLLEAMDELEVAFN   1502
Lates_calcarifer ACC1         LYLGAARVEVGTEVTDYRFFVRAIIRHSDLVTKEASFEYLHNEAERLLLEAMDELEVAFN   1466
Oncorhynchus_mykiss ACC1      LYLGAARVEVGTEVTDYRFFVRAIIRHSDLVTKEASFEYLHNEAERLLLEAMDELEVAFN   1472
Larimichthys_crocea ACC1      LYLGAARVEVGTEVTDYRFFVRAIIRHSDLVTKEASFEYLHNEAERLLLEAMDELEVAFN   1555
Consensus                     lylgaa vevgtevtdyrffvraiirhsdlvtkeasfeyl ne erllleamdelevafn

Homo_sapiens ACC1             NTNVRTDCNHIFLNFVPTVIMDPSK...................................   1524
Mus_musculus ACC1             NTNVRTDCNHIFLNFVPTVIMDPSK...................................   1486
Bos_taurus ACC1               NTNVRTDCNHIFLNFVPTVIMDPSK...................................   1487
Capra_hircus ACC1             NTNVRTDCNHIFLNFVPTVIMDPSK...................................   1524
Danio_rerio ACC1              NTTVRTDCNHIFLNFVPTVIMDPSKHSAIPSIQTILKTTLAEMQPQTRTNQRLQHVSLKA   1558
Sparus_aurata ACC1            NTTVRTDCNHIFLNFVPTVIMDPSK...................................   1527
Lates_calcarifer ACC1         NTTVRTDCNHIFLNFVPTVIMDPSK...................................   1491
Oncorhynchus_mykiss ACC1      NTTVRTDCNHIFLNFVPTVIMDPSK...................................   1497
Larimichthys_crocea ACC1      NTTVRTDCNHIFLNFVPTVIMDPSK...................................   1580
Consensus                     nt vrtdcnhiflnfvptvimdpsk

Homo_sapiens ACC1             IEESVRSMVMRYGSRLWKLRVLQAELKINIRLTPTGKAIPIRLFLTNESGYYLDISLYKE   1584
Mus_musculus ACC1             IEESVRSMVMRYGSRLWKLRVLQAELKINIRLTTTGKAIPIRLFLTNESGYYLDISLYKE   1546
Bos_taurus ACC1               IEESVRSMVMRYGSRLWKLRVLQAELKINIRLTPTGKAIPIRLFLTNESGYYLDISLYKE   1547
Capra_hircus ACC1             IEESVRSMVMRYGSRLWKLRVLQAELKINIRLTPTGKAIPIRLFLTNESGYYLDISLYKE   1584
Danio_rerio ACC1              IEESVRSMVMRYGSRLWKLRVLQAELKINIRLTPTGKQIPIRLFLTNESGYYLDISLYKE   1618
Sparus_aurata ACC1            IEESVRSMVMRYGSRLWKLRVLQAELKINIRLTPTGKQIPIRLFLTNESGYYLDISLYKE   1587
Lates_calcarifer ACC1         IEESVRSMVMRYGSRLWKLRVLQAELKINIRLTPTGKQIPIRLFLTNESGYYLDISLYKE   1551
Oncorhynchus_mykiss ACC1      IEESVRSMVMRYGSRLWKLRVLQAELKINIRLTPTGKQIPIRLFLTNESGYYLDISLYKE   1557
Larimichthys_crocea ACC1      IEESVRSMVMRYGSRLWKLRVLQAELKINIRLTPTGKQIPIRLFLTNESGYYLDISLYKE   1640
Consensus                     ieesvrsmvmrygsrlwklrvlqaelkinirlt tgk ipirlfltnesgyyldislyke

Homo_sapiens ACC1             VTDSRT.......AQIMFQAYGDKQGPLHGMLINTPYVTKDLLQSKRFQAQSLGTTYIYD   1637
Mus_musculus ACC1             VTDSRT.......AQIMFQAYGDKQGPLHGMLINTPYVTKDLLQSKRFQAQSLGTTYIYD   1599
Bos_taurus ACC1               VTDSRT.......AQIMFQAYGDKQGPLHGMLINTPYVTKDQLQSKRFQAQSLGTTYIYD   1600
Capra_hircus ACC1             VTDSRT.......AQIMFQAYGDKQGPLHGMLINTPYVTKDLLQSKRFQAQSLGTTYIYD   1637
Danio_rerio ACC1              VTDSRTGQVGHKDRQIMFQAYGDKQGPLHGMLINTPYVTKDLLQSKRFQAQSLGTTYVYD   1678
Sparus_aurata ACC1            VTDSRTGQVGPKDRQIMFQAYGDKQGPLHGMLINTPYVTKDLLQSKRFQAQSLGTTYVYD   1647
Lates_calcarifer ACC1         VTDSRTGQVGPKDRQIMFQAYGDKQGPLHGMLINTPYVTKDLLQSKRFQAQSLGTTYVYD   1611
Oncorhynchus_mykiss ACC1      VTDSRTGQLGPKDRQIMFQAYGDTQGPLHGMLINTPYVTKDLLQSKRFQAQSLGTTYVYD   1617
Larimichthys_crocea ACC1      VTDSRTGQVGPKDRQIMFQAYGDKQGPLHGMLINTPYVTKDLLQSKRFQAQSLGTTYVYD   1700
Consensus                     vtdsrt        qimfqaygd qgplhgmlintpyvtkd lqskrfqaqslgtty yd

Homo_sapiens ACC1             IPEMFRQSLIKLWESMSTQAFLESPPLPSDMLTYTELVLDDQGQLVHMNRLPGGNEIGMV   1697
Mus_musculus ACC1             IPEMFRQSLIKLWESMSTQAFLESPPLPSDILTYTELVLDDQGQLVHMNRLPGGNEIGMV   1659
Bos_taurus ACC1               IPEMFRQSLIKLWESMSSQAFLEPPPLPSDILTYTELVLDDQGQLVHMNRLPGGNEIGMV   1660
Capra_hircus ACC1             IPEMFRQSLIKLWESMSSQAFLEPPPLPSDILTYTELVLDDQGQLVHMNRLPGGNEIGMV   1697
Danio_rerio ACC1              FPEMFRQALRKLWQSMDAHANLEKCPLPSELLTFTELVLDSQGQLVQMNRLPGGNEIGMV   1738
Sparus_aurata ACC1            FPEMFRQALKKEWHSSLAYAHLEKSPLPSELLTFTELVLDAQGQLVQMNRLPGGNEIGMV   1707
Lates_calcarifer ACC1         FPEMFRQALKKMWHSSQAHAHLEKCPLPSELLTFTELVLDAQGQLVQMNRLPGGNEIGMV   1671
Oncorhynchus_mykiss ACC1      FPEMVRQALKKLWQSTKAFAHLEKCPLPSELLTFTELVLDPQGQLVQMNRLPGGNEIGMV   1677
Larimichthys_crocea ACC1      FPEMFRQALKKLWHSSQAFAHLERCPLPSELLTFTELVLDAQGQLVQMNRLPGGNEIGMV   1760
Consensus                      pem rq l k w s    a lp  plps  lt telvld qgqlv mnrlpggneigmv

Homo_sapiens ACC1             AWKMTFKSPEYPEGRDIIVIGNDITYRIGSFGPQEDLLFLRASELARAEGIPRIYVSANS   1757
Mus_musculus ACC1             AWKMSLKSPEYPDGRDIIVIGNDITYRIGSFGPQEDLLFLRASELARAEGIPRIYVAANS   1719
Bos_taurus ACC1               AWKMTLKSPEYPDGRDIIVIGNDITYRIGSFGPQEDLLFLRASELARAEGIPRIYVAANS   1720
Capra_hircus ACC1             AWKMTLKSPEYPDGRDIIVIGNDITYRIGSFGPQEDLLFLRASELARAEGIPRIYVAANS   1757
Danio_rerio ACC1              AWRMTLRTPEYPAGREIIVISNDITHKIGSFGPQEDVLFQQASEMARESGIPRIYIAANS   1798
Sparus_aurata ACC1            AWRMTLRTPEYPAGREIIVISNDITHKIGSFGPQEDVLFMRASEMARESGIPRIYIAANS   1767
Lates_calcarifer ACC1         AWRMTLRTPEYPAGREIIVISNDITHKIGSFGPQEDLLFLRASEMARESGIPRIYIAANS   1731
Oncorhynchus_mykiss ACC1      AWRMTIRTPEYPAGREIIVISNDITHKIGSFGPQEDLLFLQASEMSRESGIPRIYISANS   1737
Larimichthys_crocea ACC1      AWRMTLRTPEYPAGREIIVISNDITHKIGSFGPQEDVLFLRASEMARESGIPRIYKAANS   1820
Consensus                     aw m    peyp gr iivi ndit  igsfgpqed lf  ase  r  gipriy  ans

Homo_sapiens ACC1             GARIGLAEEIRHMFHVAWVDPEDPYKGYRYLYLTPQDYKRVSALNSVHCEHVEDEGESRY   1817
Mus_musculus ACC1             GARIGLAEEIRHMFHVAWVDPEDPYKGYKYLYLTPQDYKRVSALNSVHCEHVEDEGESRY   1779
Bos_taurus ACC1               GARIGLAEEIRHMFHVAWVDPEDPYKGYKYLYLTPQDYKRVSALNSVHCEHVEDEGESRY   1780
Capra_hircus ACC1             GARIGLAEEIRHMFHVAWVDPEDPYKGYKYLYLTPQDYKRVSALNSVHCEHVEDEGESRY   1817
Danio_rerio ACC1              GARIGLAEEIRHMFHVAWQDPVDPYKGFKYLYLTPQDYKKVSALNSVHCEHVEDEGESRY   1858
Sparus_aurata ACC1            GARIGLAEEIRHMFHVAWQDPTDPYKGFKYLYLTPQDYKKVSALNSVHCEHVEDEGESRY   1827
Lates_calcarifer ACC1         GARIGLAEEIRHMFHVAWQDPADPYKGFKYLYLTPQDYKKVSALNSVHCEHVEDEGESRY   1791
Oncorhynchus_mykiss ACC1      GARIGLAEEIRHMFHVAWQDTADPYKGFKYLYLTPQDYKKVSALNSVHCEHIEDEGESRY   1797
Larimichthys_crocea ACC1      GARIGLAEEIRHMFHVAWQDPADPYKGFKYLYLTPQDYKKVSALNSVHCEHVEDEGESRY   1880
Consensus                     gariglaeeirhmfhvaw d  dpykg  ylyltpqdyk vsalnsvhceh edegesry
```

```
Homo_sapiens ACC1           KITDIIGKEEGIGPENLRGSGMIAGESSLAYNEIITISLVTCRAIGIGAYLVRLGQRTIQ   1877
Mus_musculus ACC1           KITDIIGKEEGLGAENLRGSGMIAGESSLAYDEVITISLVTCRAIGIGAYLVRLGQRTIQ   1839
Bos_taurus ACC1             KITDIIGKEEGLGAENLRGSGMIAGESSLAYDEIITISLVTCRAIGIGAYLVRLGQRTIQ   1840
Capra_hircus ACC1           KITDIIGKEEGLGAENLRGSGMIAGESSLAYDEIITISLVTCRAIGIGAYLVRLGQRTIQ   1877
Danio_rerio ACC1            KITDIIGKEEGLGVENLRGSGMIAGESSLAYEDIITMNLVTCRAIGIGAYLVRLGQRTIQ   1918
Sparus_aurata ACC1          KITDIIGKDEGLGVENLKGSGMIAGESSLAYEEIITMNLVTCRAIGIGAYLVRLGQRTIQ   1887
Lates_calcarifer ACC1       KITDIIGKDEGLGVENLKGSGMIAGESSLAYEEIITMNLVTCRAIGIGAYLVRLGQRTIQ   1851
Oncorhynchus_mykiss ACC1    KITDIIGKEEGLGVENLKGSGMIAGESSLAYDQVITMNLVTCRAIGIGAYLVRLGQRTIQ   1857
Larimichthys_crocea ACC1    KITDIIGKDEGLGVENLKGSGMIAGESSLAYEEIITMNLVTCRAIGIGAYLVRLGQRTIQ   1940
Consensus                   kitdiigk eg g enl gsgmiagesslay   it  lvtcraigigaylvrlgqrtiq

Homo_sapiens ACC1           VENSHLILTGAGALNKVLGREVYTSNNQLGGIQIMHNNGVTHCTVCDDFEGVFTVLHWLS   1937
Mus_musculus ACC1           VENSHLILTGAGALNKVLGREVYTSNNQLGGIQIMHNNGVTHSTVCDDFEGVFTVLHWLS   1899
Bos_taurus ACC1             VENSHLILTGAGALNKVLGREVYTSNNQLGGIQIMHNNGVTHSTVCDDFEGVFTVLHWLS   1900
Capra_hircus ACC1           VENSHLILTGAGALNKVLGREVYTSNNQLGGIQIMHNNGVTHSTVCDDFEGVFTVLHWLS   1937
Danio_rerio ACC1            VENSHIILTGAGALNKVLGREVYTSNNQLGGVQIMHNNGVTHTNVCDDFEGVYTLLHWLS   1978
Sparus_aurata ACC1          VDNSHIILTGAGALNKVLGREVYTSNNQLGGIQIMHNNGVTHSTVCDDFEGVFTLLQWLS   1947
Lates_calcarifer ACC1       VDNSHIILTGAGALNKVLGREVYTSNNQLGGIQIMHNNGVTHSTVCDDFEGVFTLLQWLS   1911
Oncorhynchus_mykiss ACC1    VENSHIILTGAGALNKVLGREVYTSNNQLGGIQIMHNNGVTHSTVCDDFEGVHMLLHWLS   1917
Larimichthys_crocea ACC1    VDNSHIILTGAGALNKVLGREVYTSNNQLGGIQIMHNNGVTHCTVCDDFEGVFTLLQWLS   2000
Consensus                   v nsh iltgagalnkvlgrevytsnnqlgg qimhnngvth  vcddfegv   l wls

Homo_sapiens ACC1           YMPKSVHSSVPLLNSKDPIDRIIEFVPTKTPYDPRWMLAGRPHPTQKGQWLSGFFDYGSF   1997
Mus_musculus ACC1           YMPKSVHSSVPLLNSKDPIDRIIEFVPTKAPYDPRWMLAGRPHPTQKGQWLSGFFDYGSF   1959
Bos_taurus ACC1             YMPKSVYSSVPLLNSKDPIDRVIEFVPTKAPYDPRWMLAGRPHPTQKGQWLSGFFDYGSF   1960
Capra_hircus ACC1           YMPKSVYSSVPLLNSKDPIDRVIEFVPTKAPYDPRWMLAGRPHPTQKGQWLSGFFDYGSF   1997
Danio_rerio ACC1            YMPKNMSSPVPILSAKDPIDRAIEFVPTKAPYDPRWMLAGRPNQNIKGAWVSGFFDQGSF   2038
Sparus_aurata ACC1          YMPMNKASPVPILNAKDPIDRLVEFVPTKAPYDPRWMIAGRPSQTPKGSWQSGFFDHGSF   2007
Lates_calcarifer ACC1       YMPKCKSSPVPILSAKDPIDRPVEFVPTKAPYDPRWMLAGRPSQTPKGSWQSGFFDHGSF   1971
Oncorhynchus_mykiss ACC1    YMPKDRSSPVPIINAKDPIDRLVEFMPTKAPYDPRWMLAGRPGQTPKGPWQSGFFDHGSF   1977
Larimichthys_crocea ACC1    YMPMCKSSPVPIVHSKDPIDRPVEFVPTKAPYDPRWMLAGRPSQTPKGSWQNGFFDHGSF   2060
Consensus                   ymp    s vp    kdpidr  ef ptk pydprwm agrp     kg w  gffd gsf

Homo_sapiens ACC1           SEIMQPWAQTVVVGRARLGGIPVGVVAVETRTVELSIPADPANLDSEAKIIQQAGQVWFP   2057
Mus_musculus ACC1           SEIMQPWAQTVVVGRARLGGIPVGVVAVETRTVELSIPADPANLDSEAKIIQQAGQVWFP   2019
Bos_taurus ACC1             SEIMQPWAQTVVVGRARLGGIPVGVVAVETRTVELSIPADPANLDSEAKIIQQAGQVWFP   2020
Capra_hircus ACC1           SEIMQPWAQTVVVGRARLGGIPVGVVAVETRTVELSIPADPANLDSEAKIIQQAGQVWFP   2057
Danio_rerio ACC1            LEIMQPWAQSVVVGRARLGGIPTGVVAVETRSVELSIPADPANLDSEAKIIQQAGQVWFP   2098
Sparus_aurata ACC1          MEIMQPWAQSVVVGRARLGGIPTGVVAVETRSVELSIPADPANLDSEAKIIQQAGQVWFP   2067
Lates_calcarifer ACC1       MEIMQPWAQSVVVGRARLGGIPTGVVAVETRSVELSIPADPANLDSEAKIIQQAGQVWFP   2031
Oncorhynchus_mykiss ACC1    MEIMQPWAQSVVVGRARLGGIPTGVVAVETRSVELSIPADPANLDSEAKIIQQAGQVWFP   2037
Larimichthys_crocea ACC1    MEIMQPWAQSVVVGRARLGGIPTGVVAVETRSVELSIPADPANLDSEAKIIQQAGQVWFP   2120
Consensus                    eimqpwaq vvvgrarlggip gvvavetr velsipadpanldseakiiqqagqvwfp

Homo_sapiens ACC1           DSAFKTYQAIKDFNREGLPLMVFANWRGFSGGMKDMYDQVLKFGAYIVDGLRECCQPVLV   2117
Mus_musculus ACC1           DSAFKTYQAIKDFNREGLPLMVFANWRGFSGGMKDMYDQVLKFGAYIVDGLRECSQPVMV   2079
Bos_taurus ACC1             DSAFKTYQAIKDFNREGLPLMVFANWRGFSGGMKDMYDQVLKFGAYIVDGLRECSQPVMV   2080
Capra_hircus ACC1           DSAFKTYQAIKDFNREGLPLMVFANWRGFSGGMKDMYDQVLKFGAYIVDGLRECSQPVMV   2117
Danio_rerio ACC1            DSAFKTAQAIKDFNREGLPLIVFANWRGFSGGMKDMYDQVLKFGAYIVDGLREYRQPVLV   2158
Sparus_aurata ACC1          DSAFKTAQAIKDLNREGLPLIVFANWRGFSGGMKDMYDQVLKFGAYIVDGLREYRQPVLV   2127
Lates_calcarifer ACC1       DSAFKTAQAIKDMNREGLPLIVFANWRGFSGGMKDMYDQVLKFGAYIVDGLREYKQPVLV   2091
Oncorhynchus_mykiss ACC1    DSAFKTSQAIKDLNREGLPLMVFANWRGFSGGMKDMYDQVLKFGAYIVDGLREYCQPVLV   2097
Larimichthys_crocea ACC1    DSAFKTAQAIKDLNREGLPLIVFANWRGFSGGMKDMYDQVLKFGAYIVDGLREYQQPVLV   2180
Consensus                   dsafkt qaikd nreglpl vfanwrgfsggmkdmydqvlkfgayivdglre  qpv v

Homo_sapiens ACC1           YIPPQAELRGGSWVVIDSSINPRHMEMYADRESRGSVLEPEGTVEIKFRRKDLVKTMRRV   2177
Mus_musculus ACC1           YIPPQAELRGGSWVVIDPTINPRHMEMYADRESRGSVLEPEGTVEIKFRKKDLVKTMRRV   2139
Bos_taurus ACC1             YIPPQAELRGGSWVVIDPTINPRHMEMYADRESRGSVLEPEGTVEIKFRRKDLVKTMRRV   2140
Capra_hircus ACC1           YIPPQAELRGGSWVVIDPTINPRHMEMYADRESRGSVLEPEGTVEIKFRRKDLVKTMRRV   2177
Danio_rerio ACC1            YIPPQAELRGGSWVVIDPTINPRHMEMYADKDSRGGVLEPEGTVEIKFRKKDLVKTMRRV   2218
Sparus_aurata ACC1          YIPPQAELRGGSWVVIDPTINPRHMEMYADKDSRGGVLEPEGTVEIKFRRKDLVKTMRRV   2187
Lates_calcarifer ACC1       YIPPQAELRGGSWVVIDPTINPRHMEMYADKDSRGGVLEPEGTVEIKFRRKDLVKTMRRV   2151
Oncorhynchus_mykiss ACC1    YIPPQAELRGGSWVVIDPTINPRHMEMYADKDSRGGVLEPEGTVEIKFRKKDLVKTMRRV   2157
Larimichthys_crocea ACC1    YIPPQAELRGGSWVVIDPTINPRHMEMYADKDSRGGVLEPEGTVEIKFRRKDLVKTMRRV   2240
Consensus                   yippqaelrggswvvid  inprhmemyad  srg vlepegtveikfr kdlvktmrrv

Homo_sapiens ACC1           DPVYIHLAERLGTPELSTAERKELENKLKEREEFLIPIYHQVAVQFADLHDTPGRMQEKG   2237
Mus_musculus ACC1           DPVYIRLAERLGTPELSPTERKELESKLKEREEFLIPIYHQVAVQFADLHDTPGRMQEKG   2199
Bos_taurus ACC1             DPVYIHLAERLGTPELSVAERKELESKLKEREEFLLPIYHQVAVQFADLHDTPGRMQEKG   2200
Capra_hircus ACC1           DPVYIHLAERLGTPELSAAERKELESKLKEREEFLLPIYHQVAVQFADLHDTPGRMQEKG   2237
Danio_rerio ACC1            DPVYMGLAERLGTPELSVSERKELESKLKEREEFLLPIYHQVAVQFADLHDTPGRMQEKG   2278
Sparus_aurata ACC1          DPVYTSLAERLGTPELSPPDRKELETKLKEREEFLLPIYHQVAVQFADLHDTPGRMQEKG   2247
Lates_calcarifer ACC1       DPVYTGLAERLGTPELSPPDRKELETKLKEREEFLLPIYHQVAVQFADLHDTPGRMQEKG   2211
Oncorhynchus_mykiss ACC1    DPVYMGLAERLGTPELGLPERKELETKLKEREEFLLPIYHQVAVQFADLHDTPGRMQEKG   2217
Larimichthys_crocea ACC1    DPVYMGLAEKLGTPELSPPDRKELETKLKEREEFLLPIYHQVAVQFADLHDTPGRMQEKG   2300
Consensus                   dpvy  lae lgtpel    rkele klkereefl piyhqvavqfadlhdtpgrmqekg

Homo_sapiens ACC1           VISDILDWKTSRTFFYWRLRRLLLEDLVKKKIHNANPELTDGQIQAMLRRWFVEVEGTVK   2297
Mus_musculus ACC1           VINDILDWKTSRTFFYWRLRRLLLEDLVKKKIHNANPELTDGQIQAMLRRWFVEVEGTVK   2259
Bos_taurus ACC1             VINDILDWKTSRTFFYWRLRRLLLEDLVKKKIHNANPELTDGQIQAMLRRWFVEVEGTVK   2260
Capra_hircus ACC1           VINDILDWKTSRTFFYWRLRRLLLEDLVKKKIHNANPELTDGQIQAMLRRWFVEVEGTVK   2297
Danio_rerio ACC1            VITDILEWSTSRQFFYWRLRRLLLEETVKRKIQCANSELTDGQVQAMLRRWFVEAEGAVK   2338
Sparus_aurata ACC1          VITDILEWQTSRQFFYWRLRRLLLEETVKRKIQAANSELTDGQVQAMLRRWFVEAEGAVK   2307
Lates_calcarifer ACC1       VITDILEWQTSRQFFYWRLRRLLLEDTVKRKIQVANSELTDGQIQAMLRRWFVEAEGAVK   2271
Oncorhynchus_mykiss ACC1    VITDILEWRTSRHFFYWRLRRLLLEDTVKRKIQVANSELTDGQIQAMLRRWFVEAEGTVK   2277
Larimichthys_crocea ACC1    VITDILEWQTSRQFFYWRLRRLLLEETVKRKIQAANSELTDGQVQAMLRRWFVEAEGAVK   2360
Consensus                   vi dil w tsr ffywrlrrllle  vk ki  an eltdgq qamlrrwfve eg vk
```

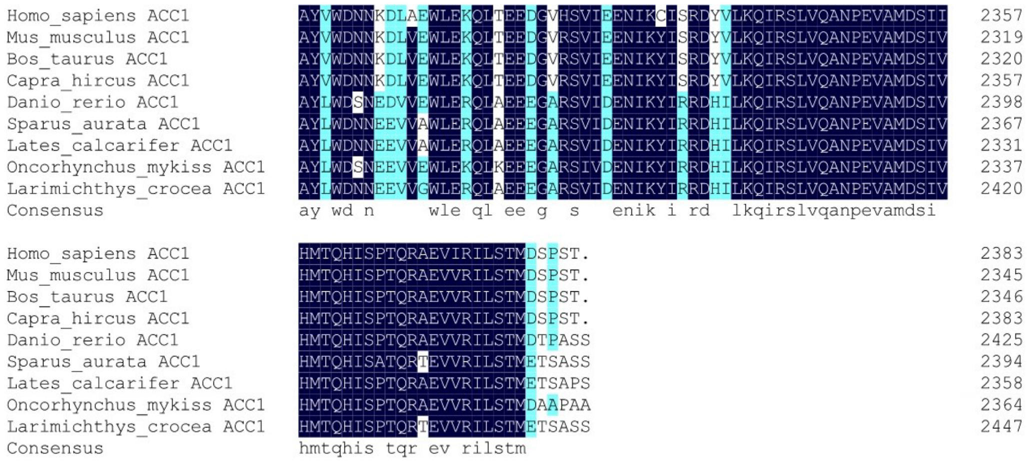

Figure S3 Multiple sequence alignment of the deduced AA sequences of large yellow croaker ACC1 with other fish species or mammals. Sequences comparison was performed using DNAMAN, and the identity/similarity shading is based on a 75% identity threshold. Identical residues are shown in dark blue shading and similar residues are shown in wathet blue shading. The identity shading is based on 89% identity threshold.

```
Homo_sapien ACC2            MVLLLCLSCLIFSCLTFSWLKIWGKMTDSKPITKSKSEANLIPSQEPFPASDNSGETPQR    60
Mus_musculu ACC2            MVLLLFLTCLVFSCLTFSWLKIWGKMTDSKPLTNSKVEANLLSSEESLSASELSGEQLQE    60
Bos_taurus ACC2             .........................MSDSKQVTKSKRKVSFTPSQEPIPASEGSQEPPQE    35
Capra_hircus ACC2           .........................MSDSKQVTKSKRKVSFTPSQEPIPASDGSQEPPQE    35
Danio_rerio ACC2            ............................................................     0
Sparus_aura ACC2            .......................MLPLAGLGLILWILLLLWWINTKTVAMPVKGEESPSG    37
Lates calcarifer ACC2       .......................MLPFAVLGLILWILLLLWRINTKTVAMPVKGEESPSG    37
Oncorhynchus mykiss ACC2    .......................MLPLLLSGFLLVLWLL.WRIKDKMMLDQGVETPTGCG    36
Larimichthys_crocea ACC2    .......................MLPFAVLGLILWILLLLWRINTKTVAMPVKGEESPSG    37
Consensus

Homo_sapien ACC2            NGEGHTLPKTPSQAEP.ASHKGPKDAGRRRNSLPPSHQKPPRNPLSSSDAAPSPE.LQAN   118
Mus_musculu ACC2            HGDHSCL...........SYRGPRDASQQRNSLPSSCQRPPRNPLSSNDTWPSPE.LQTN   108
Bos_taurus ACC2             DCNSSPLPKTPSQAEL.ASHKGPQDTCQQRPSQSPLPQNPQGNPLSSDDASPAR...QAN    91
Capra_hircus ACC2           GCNSSPLPETPRQAEL.ASHKGPQDTCQQRTSRSPPPQNPQGNPLSSDDASRAR...QAN    91
Danio_rerio ACC2            .........MPSQTP.....ISDCAGEHSPPMDPGLSSTSPRN.................    29
Sparus_aura ACC2            CDPPPAEEEVPSTQ.......SPHPGEHSSSAAPATAKRDGDN.SLQVGGASG.....VK    84
Lates calcarifer ACC2       RGSSAAKEDMPATH.......TRHSGEHSLSAVPTTSAHNDNNSALSDGTSAGPC...VK    87
Oncorhynchus mykiss ACC2    SSAAAAQEEMQAADGPTAAVHTPAPVEHSTPMVLASSTSDSGANSPQTHIDPSEGPMIPR    96
Larimichthys_crocea ACC2    CGPPAAEEDMPVTQ.......SPHPGEHFLSTAPTTSAHNEDT.SSQAGCAPAGQ...VK    86
Consensus

Homo_sapien ACC2            GTGTQGLEATDT......NGLSSSARPQGQQAGSPSKEDKKQANIKRQLMTNFILGSFDD   172
Mus_musculu ACC2            WTAAPGPEVPDA......NGLSFPARPPSQRTVSPSREDRKQAHIKRQLMTSFILGSLDD   162
Bos_taurus ACC2             GSETEGPEIPNT......NDLSAPARPQGQRARTLSREDRKQAHIKRQLMTNFILGSFDD   145
Capra_hircus ACC2           GSGTEGPEIPNT......NDLSAPARPQGQRARTLSREDRKQAHIKRQLMTNFILGSFDD   145
Danio_rerio ACC2            ..EGIPPSATSD......PTDQ...SNITVSHIESHRPRFGSGAQAK.EKLKFILGASED    77
Sparus_aura ACC2            TLDAEAPQASSE.....VNSEKPSLPPTAQNPSRTKVPMLSSGPEAR.ERLKFILGASED   138
Lates calcarifer ACC2       TVDSEPPQSSSE.....INSELPSSPQTTQKPYRTNVPMLSSGPEAR.ERLKFILGASED   141
Oncorhynchus mykiss ACC2    AGGSQPPQASSEGMTEQYTQTQPSPFAVPQPPSRTTRPKLSSGPEVRRACLKFILGASED   156
Larimichthys_crocea ACC2    AVDTEALQESSE.....VNSEK...PPTTQTSSKTKVPMLKSGPEGR.ERLKFILGASED   137
Consensus                                                                       filg   d

Homo_sapien ACC2            YSSDEDSVAGSSRESTRKGS............RASLGALSLEAYLTTGEAETRVPTMR..   218
Mus_musculu ACC2            NSSDEDPSAGSFQNSSRKSS............RASLGTLSQEAALNTSDPESHAPTMR..   208
Bos_taurus ACC2             NSSDEDAGAALFRQSSRKGS............RASLGTLSLETAHAAGETETCAPVIR..   191
Capra_hircus ACC2           NSSDEDTGAALFRQSSRKGS............RASLGTLSLETAQAAGETETCAPVIR..   191
Danio_rerio ACC2            NSSDDEP..LVKNQTTTHQPK...........NTETLNAEPTASSEMPPTPVPSTGLR..   122
Sparus_aura ACC2            NSSDEEP.LVTKPPSGASQP............QTSTDRASPQQASSAAPQSS.SSGIK..   182
Lates calcarifer ACC2       NSSDEEP.LVAKPPSGASQP............QTSSLKSSPQQEAPAVPQSS.SSGIK..   185
Oncorhynchus mykiss ACC2    NSSDDEPPLTMKPPTGSSSSEATASTVRQTASATSTAFSPPQQVQSVTLTTT.SPGMR..   213
Larimichthys_crocea ACC2    NSSDEEP.LVSKPPSGASQP............PASTNKSYPQEASTAARRSS.SSGIK..   181
Consensus                    ssd

Homo_sapien ACC2            .....................PSMSGLHLVKRGREHKKLDLHRDFTVASPAEFVTRFGGD   257
Mus_musculu ACC2            .....................PSMSGLHLVKRGREHKKLDLHRDFTVASPAEFVTRFGGN   247
Bos_taurus ACC2             .....................PSMSGLHLARRGREQKKVDLHRDFTVASPAEFVTRFAGH   230
Capra_hircus ACC2           .....................PSMSGLHLARRGREQRKVDLHRDFTVASPAEFVARFGGH   230
Danio_rerio ACC2            .....................PSMSGPHLLKKGKEHRKMEVHRDFTVASPAEFVTRFGGN   161
Sparus_aura ACC2            .....................PSMSGLHLVKKGREHRKMDLQRDFTVASPAEFVTRFGGN   221
Lates calcarifer ACC2       .....................PSMSGPHLVKKGREHRKMDLQRDFTVASPAEFVTRFGGN   224
Oncorhynchus mykiss ACC2    .....................PSMSHPHLVKKVREIRKMELQRDFTVASPAEFVTRFGGN   252
Larimichthys_crocea ACC2    .....................PSMSGLHLVRKGREHRKMDLQRDFTVASPAEFVTRFGGN   220
Consensus                                        psms  hl     e  k    rdftvaspaefv rf g

Homo_sapien ACC2            RVIEKVLIANNGIAAVKCMRSIRRWAYEMFRNERAIRFVVMVTPEDLKANAEYIKMADHY   317
Mus_musculu ACC2            RVIEKVLIANNGIAAVKCMRSIRRWAYEMFRNERAIRFVVMVTPEDLKANAEYIKMADQY   307
Bos_taurus ACC2             LVIEKVLIANNGIAAVKCMRSIRRWAYEMFRNERAIRFVVMVTPEDLKANAEYIKMADQY   290
Capra_hircus ACC2           LVIEKVLIANNGIAAVKCMRSIRRWAYEMFRNERAIRFVVMVTPEDLKANAEYIKMADQY   290
Danio_rerio ACC2            RIIDKVLIANNGIAAVKCMRSIRRWSYEMFRNERTIRFVVMVTPEDLKANAEYIKMADHY   221
Sparus_aura ACC2            RVIEKVLIANNGIAAVKCMRSIRRWSYEMFRNERTIRFVVMVTPEDLKANAEYIKMADHY   281
Lates calcarifer ACC2       RVIEKVLIANNGIAAVKCMRSIRRWSYEMFRNERTIRFVVMVTPEDLKANAEYIKMADHY   284
Oncorhynchus mykiss ACC2    RVINKVLIANNGIAAVKCMRSIRRWSYEMFRNERTIRFVVMVTPEDLKANAEYIKMADHY   312
Larimichthys_crocea ACC2    RVIEKVLIANNGIAAVKCMRSIRRWSYEMFRNERTIRFVVMVTPEDLKANAEYIKMADHY   280
Consensus                     i kvlianngiaavkcmrsirrw yemfrner irfvvmvtpedlkanaeyikmad y

Homo_sapien ACC2            VPVPGGPNNNNYANVELIVDIAKRIPVQAVWAGWGHASENPKLPELLCKNGVAFLGPPSE   377
Mus_musculu ACC2            VPVPGGPNNNNYANVELIIDIAKRIPVQAVWAGWGHASENPKLPELLCKHEIAFLGPPSE   367
Bos_taurus ACC2             VPVPGGPNNNNYANVELIVDIAKRIPVQAVWAGWGHASENPKLPELLRKHEIAFLGPPSE   350
Capra_hircus ACC2           VPVPGGPNNHNYANVELIVDIAKRIPVQAVWAGWGHASENPKLPELLHKHEIAFLGPPSE   350
Danio_rerio ACC2            VPVPGGPNNNNYANVEMIVDIAKRIPVQAVWAGWGHASENPKLPELLHKSGICFLGPSSK   281
Sparus_aura ACC2            VPVPGGANNNNYANVELIVDIAKRIPVQAVWAGWGHASENPKLPDLLNKAGISFLGPSCK   341
Lates calcarifer ACC2       VPVPGGPNNNNYANVELIVDIAKRIPVQAVWAGWGHASENPKLPELLNKAGISFLGPSSK   344
Oncorhynchus mykiss ACC2    VPVPGGANNNNYANVELIVDIAKRIPVQGVWAGWGHASENPKLPELLDKNGISFLGPPSK   372
Larimichthys_crocea ACC2    VPVPGGPNNNNYANVELIVDIAKRIPVQAVWAGWGHASENPKLPELLNKAGISFLGPSSK   340
Consensus                   vpvpgg nn nyanve i diakripvq vwagwghasenpklp ll k    flgp

Homo_sapien ACC2            AMWALGDKIASTVVAQTLQVPTLPWSGSGLTVEWTEDDLQQGKRISVPEDVYDKGCVKDV   437
Mus_musculu ACC2            AMWALGDKIASTIVAQTLQIPTLPWSGSGLTVEWTEDSRHQGKCISVPEDVYEQGCVKDV   427
Bos_taurus ACC2             AMWALGDKIASTIVAQTLQIPTLPWNGSGLTVEWAEHSLQEGQRISIPESVYNNGCVKDV   410
Capra_hircus ACC2           AMWALGDKIASTIVAQTLQIPTLPWSGSGLTVEWEEHSLQEGQRISIPESIYNNGCVKDV   410
Danio_rerio ACC2            AMWALGDKVASSIVAQSAGIPTLPWSGTGLSVEWAEEEQRQGRLISVPPELYVQGCVKDV   341
Sparus_aura ACC2            AMWALGDKVASSIVAQSADIPTLPWSGSGLRVDWAEEDQKQGRVISVPPEVYTKGCVQDV   401
Lates calcarifer ACC2       AMWALGDKVASSIVAQSADIPTLPWSGSGLRVDWAGVDQGLGNVISVPPEIYTKGCVCDV   404
Oncorhynchus mykiss ACC2    AMWALGDKVASSIVAQSADIPTLPWSGSGLRVDWTEDDQRLGHVISVPPEVYVHGCVRDA   432
Larimichthys_crocea ACC2    AMWALGDKVASSIVAQSADIPTLPWSGSGLRVDWTEEDQKLGNVISVPSEIYANGCVRDV   400
Consensus                   amwalgdk as  vaq    ptlpw g gl v w       g  is p   y  gcv d
```

```
Homo_sapien ACC2              DEGLEAAERIGFPLMIKASEGGGGKGIRKAESAEDFPILFRQVQSEIPGSPIFLMKLAQH   497
Mus_musculu ACC2              DEGLQAAEKIGFPLMIKASEGGGGKGIRKAESAEDFPMLFRQVQSEIPGSPIFLMKLAQN   487
Bos_taurus ACC2               DEGLEAAEKIGFPLMIKASEGGGGKGIRKAETAEDFPILFRQVQSETPGSPVFLMKLAQR   470
Capra_hircus ACC2             DEGLEAAEKIGFPLMIKASEGGGGKGIRKAETAEDFPILFRQVQSETPGSPIFLMKLAQH   470
Danio_rerio ACC2              DEGLASAEKIGYPVVIKASEGGGGKGIRKVESSEDFPSLFRQVQAEVPGSPIFIMQLAEH   401
Sparus_aura ACC2              DDGLAGAERIGYPVVIKASEGGGGKGIRKVESSEDFATFFRQVQTEVPGSPIFVMQLAQH   461
Lates calcarifer ACC2         DDGLAGAERIGYPVIIKASEGGGGKGIRKVESSEDFPSSFRQVQTEVPGSPIFIMQLAQH   464
Oncorhynchus mykiss ACC2      DDGLAGADRIGYPVVIKASEGGGGKGIRKVDNAEDFASFFRQVQAEVPGSPIFVMQLAQH   492
Larimichthys_crocea ACC2       DDGLAGAERIGYPVVIKASEGGGGKGIRKVECSEDFPGFFRQVQTEVPGSPIFVMQLAQH   460
Consensus                     d gl  a  ig p  ikaseggggkgirk    edf   frqvq e pgsp f m la

Homo_sapien ACC2              ARHLEVQILADQYGNAVSLFGRDCSIQRRHQKIVEEAPATIAPLAIFEFMEQCAIRLAKT   557
Mus_musculu ACC2              ARHLEVQVLADQYGNAVSLFGRDCSIQRRHQKIIEEAPATIAAPAVFEFMEQCAVLLAKM   547
Bos_taurus ACC2               ARHLEVQILADQYGNAVSLFGRDCSIQRRHQKIIEEAPATIATPAVFEFMEQCAVRLAKT   530
Capra_hircus ACC2             ARHLEVQILADQYGNAVSLFGRDCSIQRRHQKIIEEAPATIAMPAVFEFMEQCAVRLAKT   530
Danio_rerio ACC2              ARHLEVQILADQYGNAISLFGRDCSIQRRHQKIIEEAPASIASTITFEQMEQYAVRLAKM   461
Sparus_aura ACC2              ARHLEVQILADEYGNAISLFGRDCSIQRRHQKIIEEAPATIAASSTFEQMEQYAVRLAKM   521
Lates calcarifer ACC2         ARHLEVQILADEYGNAISLFGRDCSIQRRHQKIIEEAPATIAALSTFEQMERYAVRLAKM   524
Oncorhynchus mykiss ACC2      ARHLEVQILADQYGNAISLFGRDCSIQRRHQKIIEEAPATIASATTFEHMEQYAVRLAKM   552
Larimichthys_crocea ACC2       ARHLEVQILADEYGNAISLFGRDCSIQRRHQKIIEEAPATIAATSTFEQMEWCAVRLAKM   520
Consensus                     arhlevq lad ygna slfgrdcsiqrrhqki eeapa ia    fe me  a  lak

Homo_sapien ACC2              VGYVSAGTVEYLYSQDGSFHFLELNPRLQVEHPCTEMIADVNLPAAQLQIAMGVPLHRLK   617
Mus_musculu ACC2              VGYVSAGTVEYLYSQDGSFHFLELNPRLQVEHPCTEMIADVNLPAAQLQIAMGVPLHRLK   607
Bos_taurus ACC2               VGYVSAGTVEYLYSQDGSFHFLELNPRLQVEHPCTEMIADVNLPAAQLQIAMGVPLHRLK   590
Capra_hircus ACC2             VGYVSAGTVEYLYSQDGSFHFLELNPRLQVEHPCTEMIADVNLPAAQLQIAMGVPLYRLK   590
Danio_rerio ACC2              VGYVSAGTVEYLFSEDGSFHFLELNPRLQVEHPCTEMIADVNLPAAQLQIAMGIPLYRIK   521
Sparus_aura ACC2              VGYVSAGTVEYLFSEDGSFHFLELNPRLQVEHPCTEMIGDVNLPAAQLQIAMGIPLYRIK   581
Lates calcarifer ACC2         VGYVSAGTVEYLFSEDGSFHFLELNPRLQVEHPCTEMIGDVNLPAAQLQIAMGIPLHRIK   584
Oncorhynchus mykiss ACC2      VGYVSAGTVEYLFSEDGSFHFLELNPRLQVEHPCTEMIGDVNLPAAQLQIAMGIPLHRIK   612
Larimichthys_crocea ACC2       VGYVSAGTVEYLFSEDGNFHFLELNPRLQVEHPCTEMIGDVNLPAAQLQIAMGIPLYRIK   580
Consensus                     vgyvsagtveyl s dg fhflelnprlqvehpctemi dvnlpaaqlqiamg pl r k

Homo_sapien ACC2              DIRLLYGESPWGVTPISFETPSNPPLARGHVIAARITSENPDEGFKPSSGTVQELNFRSS   677
Mus_musculu ACC2              DIRLLYGESPWGVTPIPFETPLSPPIARGHVIAARITSENPDEGFKPSSGTVQELNFRSN   667
Bos_taurus ACC2               DIRLLYGESPWGVTPISFETPSNPPIPRGHVIAARITSENPDEGFKPSSGTVQELNFRSS   650
Capra_hircus ACC2             DIRLLYGESPWGMTPISFETPSNPPIARGHVIAARITSENPDEGFKPSSGTVQELNFRSS   650
Danio_rerio ACC2              DIRVLFGEAPWGDTTINFESPECMPCPRGHVIAARITSENPDEGFKPSSGTVQELNFRSS   581
Sparus_aura ACC2              DIRLLYGEPPWGDTVINFETPECMPSPRGHVIAARITSENPDEGFKPSSGTVQELNFRSS   641
Lates calcarifer ACC2         DIRLLYGETPWGDSIINFETPECMPSPRGHVIAVRITSENPDEGFKPSSGTVQELNFRSS   644
Oncorhynchus mykiss ACC2      DIRVLYGETPWGDTTINFEAPDCVPSPRGHVIAARITSENPDEGFKPSSGTVQELNFRSS   672
Larimichthys_crocea ACC2       DIRLLYGETPWGDTIINFETPDCIPSPRGHVIAARITSENPDEGFKPSSGTVQELNFRSS   640
Consensus                     dir l ge pwg   i fe p   p  rghvia ritsenpdegfkpssgtvqelnfrs

Homo_sapien ACC2              KNVWGYFSVAATGGLHEFADSQFGHCFSWGENREEAISNMVVALKELSIRGDFRTTVEYL   737
Mus_musculu ACC2              KNVWGYFSVAAAGGLHEFADSQFGHCFSWGENREEAISNMVVALKELSIRGDFRTTVEYL   727
Bos_taurus ACC2               KNVWGYFSVAATGGLHEFADSQFGHCFSWGENREEAISNMVVALKELSIRGDFRTTVEYL   710
Capra_hircus ACC2             KNVWGYFXVAATGGLHEFTDSQFGHCFSWGENREEAISNMVVALKELSIRGDFRTTVEYL   710
Danio_rerio ACC2              KNVWGYFSVGATGGLHEFADSQFGHCFSWGENREEAISNMVVAMKELSIRGDFRTTVEYL   641
Sparus_aura ACC2              KNVWGYFSVGATGGLHEFADSQFGHCFSWGENREEAISNMVVAMKELSIRGDFRTTVEYL   701
Lates calcarifer ACC2         KNVWGYFSVGATGGLHEFADSQFGHCFSWGENREEAISNMVVAMKELSIRGDFRTTVEYL   704
Oncorhynchus mykiss ACC2      KNVWGYFSVGAAGGLHEFADSQFGHCFSWGENREEAISNMVVAMKELCIRGDFRTTVEYL   732
Larimichthys_crocea ACC2       KNVWGYFSVGATGGLHEFADSQFGHCFSWGENREEAISNMVVAMKELSIRGDFRTTVEYL   700
Consensus                     knvwgyf v a gglhef dsqfghcfswgenreeaisnmvva kel irgdfrttveyl

Homo_sapien ACC2              INLLETESFQNNDIDTGWLDYLIAEKVQAEKPDIMLGVVCGALNVADAMFRTCMTDFLHS   797
Mus_musculu ACC2              VNLLETESFQNNDIDTGWLDHLIAQRVQAEKPDIMLGVVCGALNVADAMFRTCMTEFLHS   787
Bos_taurus ACC2               TNLLETENFQNNDIDTSWLDNLIAEKVQAEKPDIMLGVVCGALNVADSMFRTCMTDFLHS   770
Capra_hircus ACC2             TNLLETENFQSNDIDTSWLDNLIAEKVQAEKPDIMLGVVCGALNVADSMFRTCMTDFLHS   770
Danio_rerio ACC2              IKLLETESFRNNDIDTGWLDHLIADKVQAERPDTMLGVVCGALQVADASFRESMSDFLHS   701
Sparus_aura ACC2              IKLLETESFRNNDIDTGWLDHLIAEKVQAERPDTMLGIVCGALHVADAGFRKSMSDFLHS   761
Lates calcarifer ACC2         IKLLETESFRNNDIDTGWLDHLIAEKVQAERPDTMLGIVCGALHVADASFRKSMSDYLHS   764
Oncorhynchus mykiss ACC2      IKLLETESFRNNDIDTGWLDNLIADKVQAERPDTTLGVVCGALHVADASFRKSMSDFLHS   792
Larimichthys_crocea ACC2       IKLLETECFRNNDIDTGWLDHLIAEKVQAERPDTMLGIVCGALHVADASFRKSMSDYLHS   760
Consensus                       llete f  ndidt wld lia  vqae pd  lg vcgal vad  fr  m   lhs

Homo_sapien ACC2              LERGQVLPADSLLNLVDVELIYGGVKYILKVARQSLTMFVLIMNGCHIEIDAHRLNDGGL   857
Mus_musculu ACC2              LERGQVLPADSLLNIVDVELIYGGIKYALKVARQSLTMFVLIMNGCHIEIDAHRLNDGGL   847
Bos_taurus ACC2               LERGQVLPADSLLNIVDVELIYGGVKYILKVARQSPTMFVLIMNGSHIEIDAHRLNDGGL   830
Capra_hircus ACC2             LERGQVLPADSLLNIVDVELIYGGVKYILKVARQSPTMFVLIMNGSHIEIDAHRLNDGGL   830
Danio_rerio ACC2              LERGQVLPAASLVNTVNVDLIYDGVKYCLKVARQSPTTYVIIMNDSDIEVDVHRLSDGGL   761
Sparus_aura ACC2              LERGQVLPAASLLNSVTVDLIYEGVKYCLKVARQSPTTYVIMMNGSNIEIDVHRLNDGGL   821
Lates calcarifer ACC2         LERGQVLPAASLLNSVNVDLIYEGVKFCLKVARQSPTTYVIMMNGSNIEIDVHRLSDGGL   824
Oncorhynchus mykiss ACC2      LERGQVLPAASLLNTVDVDLIYEGVKYCLKVARQSPTTYVIMMNGSDIEIDVHRLSDGGL   852
Larimichthys_crocea ACC2       LERGQVLPAASLLNSVSVDLIYEGVKYCLKVARQSPTTYVIMMNGSNIEIDVHRLSDGGL   820
Consensus                     lergqvlpa sl n v v liy g k  lkvarqs t  v  mn   ie d hrl dggl

Homo_sapien ACC2              LLSYNGNSYTTYMKEEVDSYRITIGNKTCVFEKENDPTVLRSPSAGKLTQYTVEDGGHVE   917
Mus_musculu ACC2              LLSYNGSSYTTYMKEEVDSYRITIGNKTCVFEKENDPTVLRSPSAGKLMQYTVEDGDHVE   907
Bos_taurus ACC2               LLSCSGNSYTTYLKEEVDSYRITIGNKTCVFEKENDPTVLRSPSAGKLVKYTVADGEHVE   890
Capra_hircus ACC2             LLSCSGNSYTTYMKEEVDSYRITIGNKTCVFEKENDPTVLRSPSAGKLVKYTVADGEHVE   890
Danio_rerio ACC2              LLSYGGSSYTTYMKEEIDRYRVTVGNKTCVFEKERDPTVLRSPSAGKLLQYVVTDGSHVS   821
Sparus_aura ACC2              LLSYDGSSHTTYMKEEVDSYRITVGNKTCVFEKETDPTVLRSPSAGKLLQYIIEDGDHVC   881
Lates calcarifer ACC2         LLSYDGSSHTTYMKEEVDSYRITVGNKTCVFEKEKDPTVLRSPSAGKLLQYLVEDGGHIF   884
Oncorhynchus mykiss ACC2      LLSYNGSSYTTYMKEEVDSYRITVGNKTCVFEKERDPTVLRSSSAGKLLQYLVEDGGHIC   912
Larimichthys_crocea ACC2       LLSYDGSSHTTYMKEEVDSYRITVGNKTCVFEKEKDPTVLRSPSAGKLLQYICEDGAHVC   880
Consensus                     lls  g s tty kee d yr t gnktcvfeke dptvlrs sagkl  y   dg h
```

```
Homo_sapien ACC2               AGSSYAEMEVMKMIMTLNVQERGRVKYIKRPGAVLEAGCVVARLELDDPSKVHPAEPFTG    977
Mus_musculu ACC2               AGSSYAEMEVMKMIMTLNVQESGRVKYIKRPGVILEAGCVVARLELDDPSKVHAAQPFTG    967
Bos_taurus ACC2                AGGSYAEIEVMKMTTTLNVQESGRVKYVRRPGAVLEAGCVVARLELDDPSKVRPAEPFTG    950
Capra_hircus ACC2              AGGSYAEIEVMKMTTTLNVQESGRVKYVRRPGAVLEAGCLVARLELDDPSKVRPAEPFAG    950
Danio_rerio ACC2               ASQPYAEIEVMKMVMTLHVQHSGCIRFLKRPGTVLEPGCIVALMDLDDPSCIHQVKPNTE    881
Sparus_aura ACC2               AGETYAEIEVMKMVMTLTVQQSGCIHFVKRPGAVLEPGSVVARIDLDDHSSVHRVELNTA    941
Lates calcarifer ACC2          AGETYAEIEVMKMVMTLTVQQSGCIHFVKRPGAVLEPGCVMAHMDLDDPSSIHRVELNTA    944
Oncorhynchus mykiss ACC2       AGNSYAEIEVMKMVMTLTVVESGCVHYVKRHGAVLEPGCVVARLDLDDPSNIQPVELNTA    972
Larimichthys_crocea ACC2       AGETYAEIEVMKMVMTLTVQQSGCIHFVKRPGAVLEPGCVVAHIELDDPSSIHLVELNTA    940
Consensus                      a   yae evmkm  tl v   g       r g  le g   a   ldd s

Homo_sapien ACC2               ELPAQQTLPILGEKLHQVFHSVLENLTNVMSGFCLPEPVFSIKLKEWVQKLMMTLRHPSL   1037
Mus_musculu ACC2               ELPAQQTLPILGEKLHQVFHGVLENLTNVMSGYCLPEPFFSMKLKDWVQKLMMTLRHPSL   1027
Bos_taurus ACC2                ELPSQPTLPILGEKLHQVFHSVLENLTNIMNGYCLPEPIFSIKLKDWVQKLLRALRHPSL   1010
Capra_hircus ACC2              ELPSQPMLPILGEKLHQVFHSVLENLTNIMNGYCLPEPIFSIKLKDWVQKLLRTLRNPSL   1010
Danio_rerio ACC2               PLPAQEPLPMVGERLHQVFHNVLENLVKIMDGYCLPEPYFSQKLKNWVDTLMKTLRDPSL    941
Sparus_aura ACC2               ALPPQQPLPIVGEKLHQVFHSVLENLGKVMDGFCLEEPFFSSKLKQWVATLMKTLRDPSL   1001
Lates calcarifer ACC2          ILPPQQPLPMVGEKLHQVFHSVLENLVKVMDGYCLEEPYFSSKLKQWVATLMKTLRDPSL   1004
Oncorhynchus mykiss ACC2       SFPAQQPLPIVGEKLHQVFHMVLENLVKVMAGYCLTEPYFSNKVKQWVDTLMKTLRDPSL   1032
Larimichthys_crocea ACC2       ILPPQQPLPIVGEKLHQVFHSVLENLGKVMDGYCLEEPLFSSKLKQWVTTLMKTLRDPSL   1000
Consensus                        p q  lp  ge lhqvfh vlenl   m g cl ep fs k k wv  l   lr psl

Homo_sapien ACC2               PLLELQEIMTSVAGRIPAPVEKSVRRVMAQYASNITSVLCQFPSQ...............   1082
Mus_musculu ACC2               PLLELQEIMTSVAGRIPAPVEKAVRRVMAQYASNITSVLCQFPSQQCWASGTLGKHSTTE   1087
Bos_taurus ACC2                PLLELQEIMTSVSGRVPAPVEKAVRRVMAQYASNITSVLCQFPSQ...............   1055
Capra_hircus ACC2              PLLELQEIMTSVSGRIPAPVEKAVRRVMAQYASNITSVLCQFPSQ...............   1055
Danio_rerio ACC2               PLLELQEIMTSVAGRIPVTVEKAIRKVMAQYASNITSVLCQFPSQ...............    986
Sparus_aura ACC2               PLLELQEIMTSVAGRIPGVEKDIRKVMAQYASNITSVLCQFPSQ...............   1046
Lates calcarifer ACC2          PLLELQEIMTSVAGRIPPSVEKDIRKVMAQYASNITSVLCQFPSQ...............   1049
Oncorhynchus mykiss ACC2       PLLELQELMTSVASRIPPSVEKAIRKVMAQYASNITSVLCQFPSQ...............   1077
Larimichthys_crocea ACC2       PLLELQEIMTSVGGRIPPGVEKDIRKVMAQYASNITSVLCQFPSQ...............   1045
Consensus                      pllelqe mtsv  r p  vek  r vmaqyasnitsvlcqfpsq

Homo_sapien ACC2               ......QIATILDCHAATLQRKADREVFFINTQSIVQLVQRYRSGIRGYMKTVVLDLLRR   1136
Mus_musculu ACC2               LHPELLKIATILDCHAATLQRKADREVFFMNTQSIVQLVQRYRSGTRGYMKAVVLDLLRK   1147
Bos_taurus ACC2                ......QIATILDCHAATLQRKADREVFFMNTQSIVQLVQKYRSGARGYMKAVVLDLLRR   1109
Capra_hircus ACC2              ......QIATILDCHAATLQRKADREVFFMNTQSIVQLVQKYRSGARGYMKAVVLELLRK   1109
Danio_rerio ACC2               ......RIANILDSHAATLQRKADREIFFMNTQSIVQLVQRYRSGIRGYMKSVVLDLLKH   1040
Sparus_aura ACC2               ......RIANVLDSHAATLQRKADREVFFMNTQSIVQLVQRYRSGIRGYMKSVVLDLLKR   1100
Lates calcarifer ACC2          ......RIANILDSHAATLQRKADREVFFMNTQSIVQLVQRYRSGIRGYMKSVVLDLLRR   1103
Oncorhynchus mykiss ACC2       ......RIACILDSHAATLQKKADREVFFMNTQSIVQLVQRYRSGIRGYMKSVVLDLLKC   1131
Larimichthys_crocea ACC2       ......RIANVLDSHAATLQRKADREVFFMNTQSIVQLVQRYRSGIRGYMKSVVLDLLKR   1099
Consensus                            ia   ld haatlq kadre ff ntqsivqlvq yrsg rgymk vvl ll

Homo_sapien ACC2               YLRVEHHFQQAHYDKCVINLREQFKPDMSQVLDCIFSHAQVAKKNQLVIMLIDELCGPDP   1196
Mus_musculu ACC2               YLNVEHHFQQAHYDKCVINLREQFKPDMTQVLDCIFSHSQVAKKNQLVTMLIDELCGPDP   1207
Bos_taurus ACC2                YLLVEHHFQQAHYDKCVINLREQLKPDMAQVLDCIFSHSQVAKKNQLVIMLIDELCGPDP   1169
Capra_hircus ACC2              YLLVEHHFQQAHYDKCVINLREQLKPDMSQVLDCIFSHSQVAKKNQLVIMLIDELCGPDP   1169
Danio_rerio ACC2               YLQVEMQFQQAHYDKCVINLREQYKPDMTPVLECIFSHAQVSKKNILVTMLIDQLCGRDP   1100
Sparus_aura ACC2               YLEVETQFQQAHYDKCVINLRERHKPDMSPVLEYIFSHAQVSKKNVLVTMLIDQLCGRDP   1160
Lates calcarifer ACC2          YLQVEMQFQQAHYDKCVINLREQHKPDMSPVLEYIFSHAQVSKKNILVIMLIDQLCARDP   1163
Oncorhynchus mykiss ACC2       YLQVEMQFQQAHYDKCVINLREQYKPDMTPVLESIFSHAQVSKKNVLVTILIDQLCGRDP   1191
Larimichthys_crocea ACC2       YLQVEMQFQQAHYDKCVINLREQHKPDMSRVLEYIFSHAQVSKKNILVTMLIDQLCGRDS   1159
Consensus                      yl ve  fqqahydkcvinlre  kpdm  vl  ifsh qv kkn lv  lid lc  d

Homo_sapien ACC2               SLSDELISILNELTQLSKSEHCKVALRARQILIASHLPSYELRHNQVESIFLSAIDMYGH   1256
Mus_musculu ACC2               TLSDELTSILCELTQLSRSEHCKVALRARQVLIASHLPSYELRHNQVESIFLSAIDMYGH   1267
Bos_taurus ACC2                SLSDELTSILDELTQLSKSEHCKVALRARQVLIASHLPSYELRHNQVESIFLSAIDMYGH   1229
Capra_hircus ACC2              SLSDELTSILDELTQLSKSEHCKVALRARQVLIASHLPSYELRHNQVESIFLSAIDMYGH   1229
Danio_rerio ACC2               MLADELMVILDELTQLSKMENSKVALRARQVLIASHLPSYELRHNQVESIFLSAIDMYGH   1160
Sparus_aura ACC2               TLADELMVILNELTQLSKMENSKVALRARQVLIASHLPSYELRHNQVESIFLSAIDMYGH   1220
Lates calcarifer ACC2          TLADELMAILNEFTQLSKMENSKVALRARQVLIASHLPSYELRHNQVESIFLSAIDMYGH   1223
Oncorhynchus mykiss ACC2       TLADELMVILNELTQLNKMENSKVALRARQVLIASHLPSYELRHNQVESIFLSAIDMYGH   1251
Larimichthys_crocea ACC2       TLAEELMAILNELTQLSKMENSKVALRARQVLIASHLPSYELRHNQVESIFLSAIDMYGH   1219
Consensus                       l  el  il e tql    e  kvalrarq liashlpsyelrhnqvesiflsaidmygh

Homo_sapien ACC2               QFCPENLKKLILSETTIFDVLPTFFYHANKVVCMASLEVYVRRGYIAYELNSLQHRQLPD   1316
Mus_musculu ACC2               QFCPENLKKLILSETTIFDVLPTFFYHENKVVCMASLEVYVRRGYIAYELNSLQHRELPD   1327
Bos_taurus ACC2                QFCPENLKKLILSETTIFDVLPTFFYHANKVVCMASLEVYVRRGYIAYELTSLQHRQFPD   1289
Capra_hircus ACC2              QFCPENLKKLILSETTIFDVLPTFFYHANKVVCMASLEVYVRRGYIAYELTSLQHRQFPD   1289
Danio_rerio ACC2               QFCPENLKKLILSETSIFDVLPSFFYHNNRVVCMAALEVYVRRAYIAYELNSLQHHQLQD   1220
Sparus_aura ACC2               QFCPENLKKLILSETSIFDVLPNFFYHSNQVVCMAALEVYVRRAYIAYELNSIQHHQLQD   1280
Lates calcarifer ACC2          QFCPENLKKLILSETSIFDVLPNFFYHSNQVVCMAALEVYVRRAYIAYELNSIQHHQLQD   1283
Oncorhynchus mykiss ACC2       QFCPENLKKLILSETSIFDVLPNFFYHSNRVVCMAALEVYVRRGYIAYELNSLQHHQLQD   1311
Larimichthys_crocea ACC2       QFCPENLKKLILSETSIFDVLPNFFYHSNQVVCMAALEVYVRRAYIAYELNSIQHHQLQD   1279
Consensus                      qfcpenlkklilset ifdvlp ffyh n vvcma levyvrr yiayel s qh    d

Homo_sapien ACC2               GTCVVEFQFMLPSSHPN.............................RMTVPISITNPD.   1345
Mus_musculu ACC2               GTCVVEFQFMLPSSHPN.............................RMAVPISVSNPD.   1356
Bos_taurus ACC2                GTCVVEFQFMLPSSHPH.............................RMTVPISVTNPE.   1318
Capra_hircus ACC2              GTCVVEFQFMLPSSHPH.............................RMTMPISVTNPE.   1318
Danio_rerio ACC2               GTCAVDFQFMLPSSHPNREPNATVSQHMPKSTGSSPTLN........RLTLPVNDSGEFP   1272
Sparus_aura ACC2               GTCAVDFQFMLPSSHPNR.............GSSPTLNSLCVCLVSRVPVPVSGSGQF.   1325
Lates calcarifer ACC2          GTCAVDFQFMLPSSHPNR.............GSSPTLN........RVPVPMNGSGQF.   1320
Oncorhynchus mykiss ACC2       GTCAVDFQFMLPSSHPNR.............GSSPTLN........RLTMPVNSVGQF.   1348
Larimichthys_crocea ACC2       GSCAVDFQFMLPSSHPNR.............GSSPTLN........RVPVPVSGSGQF.   1316
Consensus                      g c v fqfmlpsshp                               r   p
```

```
Homo_sapien ACC2          .LLRHSTELFMDSGFSPLCQRMGAMVAFRRFEDFTRNFDEVISCFANVPKDTPLFSEART  1404
Mus_musculu ACC2          .LLRHSTELFMDSGFSPLCQRMGAMVAFRRFEEFTRNFDEVISCFANVQTDTLLFSKACT  1415
Bos_taurus ACC2           .LLRHSTELFMDSGFSPKSQRMGAMVAFQRFEDFVRNFDEVISCFANMPKDTPLFSEARN  1377
Capra_hircus ACC2         .LLRHSTELFMDSGFSPKSQRIGAMVAFQRFEDFVRNFDEVISCFANVPKDTPLFSKARN  1377
Danio_rerio ACC2          AMRRQGSELFLEGALSPPCQRMGAMVAFHSFDHFKRCFDEVICRFVDPLCESSLFFDGCS  1332
Sparus_aura ACC2          KMRRQSSELFLDGSLSPPCQRMGAMVAFQCVDDFKRNFDEVLSSFAEPLLETAPYSES.S  1384
Lates calcarifer ACC2     KMRRQGSELFLEGAFSPPCQRMGAMVAFQCFDDFRRNFDEVLSSFAEPLLESTPFSESCS  1380
Oncorhynchus mykiss ACC2  EMRRQGSELFLDGAFSPPCQRMGAMVAFQCFEDFKRNFDEVISSFADPLLESPLFPEACP  1408
Larimichthys_crocea ACC2  KMRRQSSELFLEGALSPPCQRMGAMVAFQCFDDFKRNVDEVLSSFAEPLLESSPFSEACS  1376
Consensus                    r   elf     sp  qr gamvaf     f r  dev   f

Homo_sapien ACC2          SLYSEDDCKSLREEPIHILNVSIQCADHLEDEALVPILRTFVQSKKNILVDYGLRRITFL  1464
Mus_musculu ACC2          SLYSEEDSKSLREEPIHILNVAIQCADHMEDEALVPVFRAFVQSKKHILVDYGLRRITFL  1475
Bos_taurus ACC2           SLYSEDDSKSLREEPIHILNVALQHADHQEDEQLVPIFRTFVQSKKNILVECGLRRITFL  1437
Capra_hircus ACC2         SLYSEDDSKSLREEPIHILNVALQHADHQEDEQLVPIFRTFVQSKKNILVECGLRRITFL  1437
Danio_rerio ACC2          TFCDGESCKNMKENPIHIINVSIKQADTEDDDALVTAFTAFAHSKKSLLYDYGIRRVTFL  1392
Sparus_aura ACC2          SLYEEDNFRNKRENPIHIINVSIKTADTEDDDALVTAFTEFAQSKKAVLFEYGIRRITFL  1444
Lates calcarifer ACC2     SLYEEENFKNTRENPIHIINVSIKTADTEDDDALVKAFTAFAQSKRAVLFEYGIRRITFL  1440
Oncorhynchus mykiss ACC2  SLYEEDNCKNMKENPIHIINVSIKSADTEDDDTLVTAFAAFAQSKKVLLFEHGIRRITFL  1468
Larimichthys_crocea ACC2  SLYEDENLKNTRENPIHIINVSIKTADTEDDDTLVTALTAFAQSKKAVLFEYGIRRITFL  1436
Consensus                             e pihi nv    ad   d  lv     f  sk   l   g rr tfl

Homo_sapien ACC2          IAQEKEFPKFFTFRARDEFAEDRIYRHLEPALAFQLELNRMRNFDLTAVPCANHKMHLYL  1524
Mus_musculu ACC2          VAQEREFPKFFTFRARDEFAEDRIYRHLEPALAFQLELSRMRNFDLTAVPCANHKMHLYL  1535
Bos_taurus ACC2           IAQEKEFPKFFTFRARDEFAEDRIYRHLEPALAFQLELSRLRNFDVTAMPCANHKMHLYL  1497
Capra_hircus ACC2         IAQEKEFPKFFTFRARDEFAEDRIYRHLEPALAFQLELSRLRNFDVTAMPCANHKMHLYL  1497
Danio_rerio ACC2          VAQKREFPKYFTFRARDEFHEDRIYRNLEPALAFQLELNRMRNFDLKAVPCAHHRMQLYL  1452
Sparus_aura ACC2          IAQKREFPKFFTFRARDGFQEDRIYRNLEPALAFQLELNRMRNFELTAVPCANHKMQLYL  1504
Lates calcarifer ACC2     IAQKREFPKFFTFRARDGFQEDRIYRNLEPALAFQLELNRMRNFDLTAVPCANHKMHLYL  1500
Oncorhynchus mykiss ACC2  VAQRREFPKFFTFRARDGFQEDRIYRNLEPALAFQLELNRMRNFDLTAVPCANSKMHQYL  1528
Larimichthys_crocea ACC2  IAQKREFPKFFTFRARDGFQEDRIYRNLEPALAFQLELNRMRNFDLTAVPCANHKMQLYL  1496
Consensus                  aq  efpk ftfrard f edriyr lepalafqlel r rnf   a pca   m  yl

Homo_sapien ACC2          GAAKVKEGVEVTDHRFFIRAIIRHSDLITKEASFEYLQNEGERLLLEAMDELEVAFNNTS  1584
Mus_musculu ACC2          GAAKVKEGLEVTDHRFFIRAIIRHSDLITKEASFEYLQNEGERLLLEAMDELEVAFNNTS  1595
Bos_taurus ACC2           GAARVKEGAEVTDHRFFIRVIIRHSDLITKEASFEYLQNEGERLLLEAMDELEVAFNNTS  1557
Capra_hircus ACC2         GAARVKEGAEVTDHRFFIRVIIRHSDLITKEASFEYLQNEGERLLLEAMDELEVAFSNTS  1557
Danio_rerio ACC2          GAARVEEGAEVTDYRFFIRAIIRHSDLITKEASFEYLQNEGERLLLEAMDELEVAFSNTP  1512
Sparus_aura ACC2          GAARVQEGAEVTDYRFFIRAIIRHSDLITKEASFEYLQNEGERLLLEAMDELEVAFSNTS  1564
Lates calcarifer ACC2     GAARVQEGAEVTDYRFFIRAIIRHSDLITKEASFEYLQNEGERLLLEAMDELEVAFSNTS  1560
Oncorhynchus mykiss ACC2  GAARVQEGAEVTDYRYFIRAIMRHSDLITKEASFEYLQNEGERLLLEAMDELEVAFSNTK  1588
Larimichthys_crocea ACC2  GAARVQEGAEVTDYRFFIRAIIRHSDLITKEASFEYLQNEGERLLLEAMDELEVAFSNTS  1556
Consensus                 gaa v eg evtd r fir i rhsdlitkeasfeylqnegerllleamdelevaf nt

Homo_sapien ACC2          VRTDCNHIFLNFVPTVIMDPFKIEESVRYMVMRYGSRLWKLRVLQAEVKINIRQTTTGSA  1644
Mus_musculu ACC2          VRTDCNHIFLNFVPTVIMDPLKIEESVRDMVMRYGSRLWKLRVLQAEVKINIRQTTSDSA  1655
Bos_taurus ACC2           VRTDCNHIFLNFVPTVIMDPSKIEESVRSMVMRYGSRLWKLRVLQAEVKINIRQTTADSA  1617
Capra_hircus ACC2         VRTDCNHIFLNFVPTVIMDPSKIEESVRSMVMRYGSRLWKLRVLQAEVKINIRQTTTDSA  1617
Danio_rerio ACC2          TRTDCNHIFLNFVPTVIMDPYKIEESVRSMVMRYGSRLWKLRVLQAELKINIRLTTTGDV  1572
Sparus_aura ACC2          VRTDCNHIFLNFVPTVIMDPSKIEESVRSMVMRYGSRLWKLRVLQAELKINIRLTPNGNA  1624
Lates calcarifer ACC2     VRTDCNHIFLNFVPTVIMDPSKIEESVRSMVMRYGSRLWKLRVLQAELKINIRLTPTGNA  1620
Oncorhynchus mykiss ACC2  WRTDCNHIFLNFVPTVIMDPSKIEESVRSMVMRYGSRLWKLRVLQAELKINIRLTPTGTA  1648
Larimichthys_crocea ACC2  VRTDCNHIFLNFVPTVIMDPSKIEESVRSMVMRYGSRLWKLRVLQAELKINIRLTPTGNA  1616
Consensus                  rtdcnhiflnfvptvimdp kieesvr mvmrygsrlwklrvlqae kinir t

Homo_sapien ACC2          VPIRLFITNESGYYLDISLYKEVTDSRSGNIMFHSFGNKQGPQHGMLINTPYVTKDLLQA  1704
Mus_musculu ACC2          IPIRLFITNESGYYLDISLYREVTDSRSGNIMFHSFGNKQGSLHGMLINTPYVTKDLLQA  1715
Bos_taurus ACC2           IPIRLFITNESGYYLDISLYKEVTDPRSGNILFHSFGNKQGPQHGMLINTPYVTKDLLQA  1677
Capra_hircus ACC2         IPIRLFITNESGYYLDISLYKEVTDPRSGNILFHSFGSKQGPQHGMLINTPYVTKDLLQA  1677
Danio_rerio ACC2          IPIRLFLTNESGYYLDISLYKEVNDPSSGQIMFQSYGDKQGPLNGMLINTPYVTKDLLQA  1632
Sparus_aura ACC2          IPVRLFLTNESGYYLDISLYKEVTDPSSGQIMFLSYGDKQGPLHGMLINTPYVTKDLLQA  1684
Lates calcarifer ACC2     VPIRLFLTNESGYYLDISLYKEVTDPSSGQIMFQSYGDKQGPLHGMLINTPYVTKDLLQA  1680
Oncorhynchus mykiss ACC2  IPIRLFLTNESGYYLDISLYKEVTDPSTGQIMFQSYGDKQGPLHGMLINHPYVTKDLLQS  1708
Larimichthys_crocea ACC2  IPVRLFLTNESGYYLDISLYKEVTDPSSGQIMFQSYGDKQGPLHGMLINTPYVTKDLLQA  1676
Consensus                  p rlf tnesgyyldisly ev d   g i f s g kqg   gmlin pyvtkdllq

Homo_sapien ACC2          KRFQAQTLGTTYIYDFPEMFRQALFKLWGSPDKYPKDILTYTELVLDSQGQLVEMNRLPG  1764
Mus_musculu ACC2          KRFQAQSLGTTYVYDFPEMFRQALFKLWGSPEKYPKDILTYTELVLDSQGQLVEMNRLPG  1775
Bos_taurus ACC2           KRFQAQSLGTTYVYDFPEMFRQALFKMWPSPDKYPKDILTYTELVLDPQGQLVEMNRLPG  1737
Capra_hircus ACC2         KRFQAQSLGTTYVYDFPEMFRQALFKMWHSSDKYPKDILTYTELVLDPQGQLVEMNRLPG  1737
Danio_rerio ACC2          KRFQAQSLGTTYVYDFPEMFRQALFKLWGPGDSYPKDVLMCNELVLDSQGNLVQMNRLPG  1692
Sparus_aura ACC2          KRFQAQTLGTTYIYDFPEMFRQALFKLWGPGDKHPKDVLMCIELVLDPQGRLVQMNRQPG  1744
Lates calcarifer ACC2     KRFQAQTLGTTYVYDFPEMFRQALFKLWGPGDKCPKDVLMCTELVLDPQGRLVQMNRLPG  1740
Oncorhynchus mykiss ACC2  KRFQAQTLGTTYVYDFPEMFRQALFKLWGPGDSYPKDVLMCTELVLDPHDRLVQMNRLPG  1768
Larimichthys_crocea ACC2  KRFQAQTLGTTYVYDFPEMFRQALFKQWGSGDKHPKDVLMCTELVLDPQGRLVQMNRLPG  1736
Consensus                 krfqaq lgtty ydfpemfrqalfk w      pkd l   elvld    lv mnr pg
```

```
Homo_sapien ACC2               GNEVGMVAFKMRFKTQEYPEGRDVIVIGNDITFRIGSFGPGEDLLYLRASEMARAEGIPK  1824
Mus_musculu ACC2               CNEVGMVAFKMRFKTPEYPEGRDAVVIGNDITFQIGSFGIGEDFLYLRASEMARTEGIPQ  1835
Bos_taurus ACC2                GNEVGMVAFKMTLKTLEYPEGRDIILISNDITFRIGSFGPGEDLLYLRASELARAEGIPR  1797
Capra_hircus ACC2              GNEVGMVAFKMRFKTLEYPEGRDIILISNDITFHIGSFGPGEDLLYLRASELARAEGIPK  1797
Danio_rerio ACC2               DNEVGMVAFRMRMKTPEYPEGRDIIVICNDITHMIGSFGPQEDQLFMRASELARAEGIPR  1752
Sparus_aura ACC2               DNDVGMVAFRMKMKTPEYPEGRDIVVICNDITHMIGSFGPQEDELFLKASELARAEGIPR  1804
Lates calcarifer ACC2          DNDVGMVAFRMKMKTPEYPEGRDIIVICNDITHMIGSFGPQEDELYLRASELARAEGIPR  1800
Oncorhynchus mykiss ACC2       DNEVGMVAFRMRMKTPEYPEGRDIIIICNDITYMIGSFGPQEDQLFLRASEMARAEGIPR  1828
Larimichthys_crocea ACC2       DNDVGMVAFRMKMKTPEYPEGRDIIVICNDITHMIGSFGPPEDELFLKASELARAEGIPR  1796
Consensus                       n vgmvaf m  kt eypegrd   i ndit  igsfg  ed l    ase ar egip

Homo_sapien ACC2               IYVAANSGARIGMAEEIKHMFHVAWVDPEDPHKGFKYLYLTPQDYTRISSLNSVHCKHIE  1884
Mus_musculu ACC2               IYLAANSGARMGLAEEIKQIFQVAWVDPEDPHKGFRYLYLTPQDYTQISSQNSVHCKHIE  1895
Bos_taurus ACC2                VYLAANSGARIGLAEEIKHMFQVAWVDPEDPHKGIKYLYLTPQDYTRISSLNSVHCKHVE  1857
Capra_hircus ACC2              VYLAANSGARIGLAEEIKHMFQVAWVDPEDPHKGIKYLYLTPQDYTRISSLNSVHCKHVE  1857
Danio_rerio ACC2               IYISANSGARIGLAEEIRHMFQVAWIDPEDPYKGFKYLYLTPQDYTRISSSNSVHCHHVE  1812
Sparus_aura ACC2               IYIAANSGARIGLAEEIKHMFQVAWIDPADPYKGFKYLYLTPQDYTRISSTSAVHCHHVE  1864
Lates calcarifer ACC2          VYIAANSGARIGLAEEIKHMFQVAWIDPADPYKGFKYLYLTPQDYTRISSTNAVHCRHVE  1860
Oncorhynchus mykiss ACC2       IYISANSGARIGLAEEIRHMFQVAWTDPQDPYKGFKYLYLTPQDYTRISSTNAVHCHHVE  1888
Larimichthys_crocea ACC2       IYIAANSGARIGLAEEIKHMFQVAWIDESDPYKGFKYLYLTPQDYTRISSTSAVHCRHVE  1856
Consensus                       y  ansgar g aeei   f vaw dp dp kg  ylyltpqdyt iss    vhc h e

Homo_sapien ACC2               EGGESRYMITDIIGKDDGLGVENLRGSGMIAGESSLAYEEIVTISLVTCRAIGIGAYLVR  1944
Mus_musculu ACC2               DEGESRYVIVDVIGKDANLGVENLRGSGMIAGEASLAYEKTVTISMVTCRALGIGAYLVR  1955
Bos_taurus ACC2                EDGESRYVITDIIGKEEGLGVENLRGSGMIAGETSQDYDEIVTISMVSCRALGIGAYLVR  1917
Capra_hircus ACC2              EDGESRYVITDIIGKEEGLGVENLRGSGMIAGETSQDYDEIVTISMVTCRALGIGAYLVR  1917
Danio_rerio ACC2               EGGESRYILTDIIGKEEGIGVENLRGSGTIAGETSQAYKEIITISMVTCRAIGIGAYLVR  1872
Sparus_aura ACC2               EGGESRYIITDIIGNDDGIGVENLRGSGMIAGESSQAYEEIITISMVTCRAIGIGAYLVR  1924
Lates calcarifer ACC2          EGGESRYIITDIIGKVDGLGVENLRGSGTIAGETSQAYEEIITISMVTCRAIGIGAYLVR  1920
Oncorhynchus mykiss ACC2       EGGESRYIITDIIGTEDGLGVENLRGSGTIAGESSQAYDEIITISMVTCRAIGIGAYLVR  1948
Larimichthys_crocea ACC2       EGGESRYIITDIIGKDDGLGVENLRGSGTIAGESSQAYEEIITISMVTCRAIGIGAYLVR  1916
Consensus                        gesry   d ig     gvenlrgsg iage s  y     tis v cra gigaylvr

Homo_sapien ACC2               LGQRVIQVENSHIILTGASALNKVLGREVYTSNNQLGGVQIMHYNGVSHITVPDDFEGVY  2004
Mus_musculu ACC2               LGQRVIQVENSHIILTGAGALNKVLGREVYTSNNQLGGVQIMHTNGVSHVTVPDDFEGVC  2015
Bos_taurus ACC2                LGQRVIQVENSHIILTGATALNKVLGRDVYTSNNQLGGVQIMHHNGVSHVTVPDDFEGVC  1977
Capra_hircus ACC2              LGQRVIQVENSHIILTGATALNKVLGREVYTSNNQLGGVQIMHYNGVSHVTVPDDFEGVC  1977
Danio_rerio ACC2               LGQRVIQVENSHIILTGAGALNKVLGREVYTSNNQLGGVQIMHNNGVTHSTVPDDFEGVL  1932
Sparus_aura ACC2               LGQRVIQVENSHIILTGAGALNKVLGREVYTSNNQLGGIQIMHNNGVTHTTVPDDFEGVF  1984
Lates calcarifer ACC2          LGQRVIQVENSHIILTGAGALNKVLGREVYTSNNQLGGVQIMHNNGVTHTTVPDDFEGVF  1980
Oncorhynchus mykiss ACC2       LGQRVIQVENSHIILTGAGALNKVLGREVYTSNNQLGGVQIMHNNGVTHTTVPDDFEGVF  2008
Larimichthys_crocea ACC2       LGQRVIQVENSHIILTGAGALNKVLGREVYTSNNQLGGIQIMHNNGVTHTTVPDDFEGVF  1976
Consensus                      lgqrviqvenshiiltga alnkvlgr vytsnnqlgg qimh ngv h tvpddfegv

Homo_sapien ACC2               TIIEWLSYMPKDNHSPVPIITPTDPIDREIEFLPSRAPYDPRWMLAGRPHPTLKGTWQSG  2064
Mus_musculu ACC2               TIIEWLSFIPKDNRSPVPITTPSDPIDREIEFTPTKAPYDPRWMLAGRPHPTLKGTWQSG  2075
Bos_taurus ACC2                TIIEWLSYMPKDNRSPVPVVTPKDPIDREIEFQPSRGPYDPRWLLAGRPHPTLKGSWQSG  2037
Capra_hircus ACC2              TIIEWLSYMPKDNRSPVPVITPKDPIDREIEFQPSRGPYNPRWLLAGRPHPTLKGSWQSG  2037
Danio_rerio ACC2               TIIQWLSYMPKSNQSPVPIKPATDPVDREIDFVPTKAPYDPRWLLCGRPHPTVKGAWQSG  1992
Sparus_aura ACC2               TIIKWLSYMPKNKQSPVPVIATADPVDREIDYTPTKAPYDPRWMLAGRPHPSVRGAWQSG  2044
Lates calcarifer ACC2          TIIQWLSYMPKNKHSPVPVIATTDPVDREIEFTPTKAPYDPRWMLAGRPHPTAKGTWQSG  2040
Oncorhynchus mykiss ACC2       TIIRWLSYMPKTKHSPVPVIPPKDPVEREIEFTPTKAPYDPRWMLAGRPHPTVKGAWQSG  2068
Larimichthys_crocea ACC2       TIIRWLSYMPKNKYSPVPVISTTDPVDREIEYTPTKAPYDPRWMLAGRPHPTVRGAWQSG  2036
Consensus                      til wls  pk   spvp     dp rei   p   py prw l grphp   g wqsg

Homo_sapien ACC2               FFDHGSFKEIMAPWAQTVVTGRARLGGIPVGVIAVETRTVEVAVPADPANLDSEAKIIQQ  2124
Mus_musculu ACC2               FFDHGSFKEIMAPWAQTVVTGRARLGGIPVGVIAVETRTVEVAVPADPANLDSEAKIIQQ  2135
Bos_taurus ACC2                FFDQGSFKEIMVPWAQTVVTGRARLGGIPVGVIAAETRTVELVVPADPANLDSEAKIIQQ  2097
Capra_hircus ACC2              FFDQGSFKEIMVPWAQTVVTGRARLGGIPVGVIAAETRTVEVVVPADPANLDSEAKIIQQ  2097
Danio_rerio ACC2               FFDHGSFLEIMATWAQTVVVGRARLGGIPLGVIAVETRTVELAIPADPANLDSEAKLLQQ  2052
Sparus_aura ACC2               FFDQGSFMEIMESWARTVVTGRARLGGIPLGVIAVETRSVELTIPADPANLDSESKVLQQ  2104
Lates calcarifer ACC2          FFDHGSFMEIMGSWAQTVVVGRARLGGIPLGVIAVETRSVEFTVPADPANLDSESKVLQQ  2100
Oncorhynchus mykiss ACC2       FCDHGSFMEVMGSWAQTVVVGRARLGGIPLGVIAVETRTVEVAIPADPANLDSEARLVQQ  2128
Larimichthys_crocea ACC2       FFDHGSFMEIMESWAQTVVVGRARLGGIALGVIAVETRTVEFTVPADPANLDSESKVLQQ  2096
Consensus                      f d gsf e m  wa tvv grarlggi  gvia etr ve   padpanldse    qq

Homo_sapien ACC2               AGQVWFPDSAYKTAQAVKDFNREKLPLMIFANWRGFSGGMKDMYDQVLKFGAYIVDGLRQ  2184
Mus_musculu ACC2               AGQVWFPDSAYKTAQVIRDFNKERLPLMIFANWRGFSGGMKDMYEQMLKFGAYIVDGLRL  2195
Bos_taurus ACC2                AGQVWFPDSAYKTAQAIKDFNREKLPLMIFANWRGFSGGMKDMYDQVLKFGAYIVDGLRK  2157
Capra_hircus ACC2              AGQVWFPDSAYKTAQAINDFNREKLPLMIFANWRGFSGGMKDMYDQVLKFGAYIVDGLRK  2157
Danio_rerio ACC2               AGQVWFPDSAYKTAQAIEDFNREKLPLMVFANWRGFSGGMKDMYDQVLKFGSYIVDALRE  2112
Sparus_aura ACC2               AGQVWFPDSAFKTAQAICDFNREKLPLMVFANWRGFSGGMKDMYDQVLKFGAYIVDALRG  2164
Lates calcarifer ACC2          AGQVWFPDSAFKTAQAICDFNRERLPLMVFANWRGFSGGMKDMYDQVLKFGAYIVDALHG  2160
Oncorhynchus mykiss ACC2       AGQVWFPDSAFKTAQAIRDFNRERLPLMVFANWRGFSGGMKDMYDQVLKYGAYIVDGLRE  2188
Larimichthys_crocea ACC2       AGQVWFPDSAFKTAQAISDFNRERLPLMVFANWRGFSGGMKDMYDQILKFGAYIVDALRG  2156
Consensus                      agqvwfpdsa ktaq   dfn e lplm fanwrgfsggmkdmy q lk g yivd l

Homo_sapien ACC2               YKQPILIYIPPYAELRGGSWVVIDATINPLCIEMYADKESRGGVLEPEGTVEIKFRKKDL  2244
Mus_musculu ACC2               YEQPILIYIPPCAELRGGSWVVLDSTINPLCIEMYADKESRGGVLEPEGTVEIKFRKKDL  2255
Bos_taurus ACC2                YRQPVLIYIPPYAEVRGGSWAVMDTSINPLCIEMYADRESRASVLEPEGTVEIKYQKKDL  2217
Capra_hircus ACC2              YRQPVLIYIPPHAEVRGGSWAVMDASINPLCIEIYADRDSRASVLEPEGTVEIKYRKKDL  2217
Danio_rerio ACC2               FSQPVLVYIPPNAELRGGSWVVIDPTINLQHMELYADRESRGGVLEAEGTVEIKFRKKDL  2172
Sparus_aura ACC2               FHQPVLVYIPPHAELRGGSWVVIDPTINPLCMELYADKDSRGGVLEAEGTVEIKFRRKDL  2224
Lates calcarifer ACC2          FHQPVLVYIPPQAELRGGSWVVIDPTINPLCMELYADRESRGGVLEAEGTVEIKFRRKDL  2220
Oncorhynchus mykiss ACC2       FRQPVLIYIPPHAELRGGSWVVIDPTINLLCMELYADRESRGGVLEAEGTVEIKYRRKDL  2248
Larimichthys_crocea ACC2       FHQPVLVYIPPQAELRGGSWVVIDPTINPLCMELYADRESRGGVLEAEGTVEIKFRRKDL  2216
Consensus                        qp l yipp ae rggsw v d  in     e yad  sr   vle egtveik   kdl
```

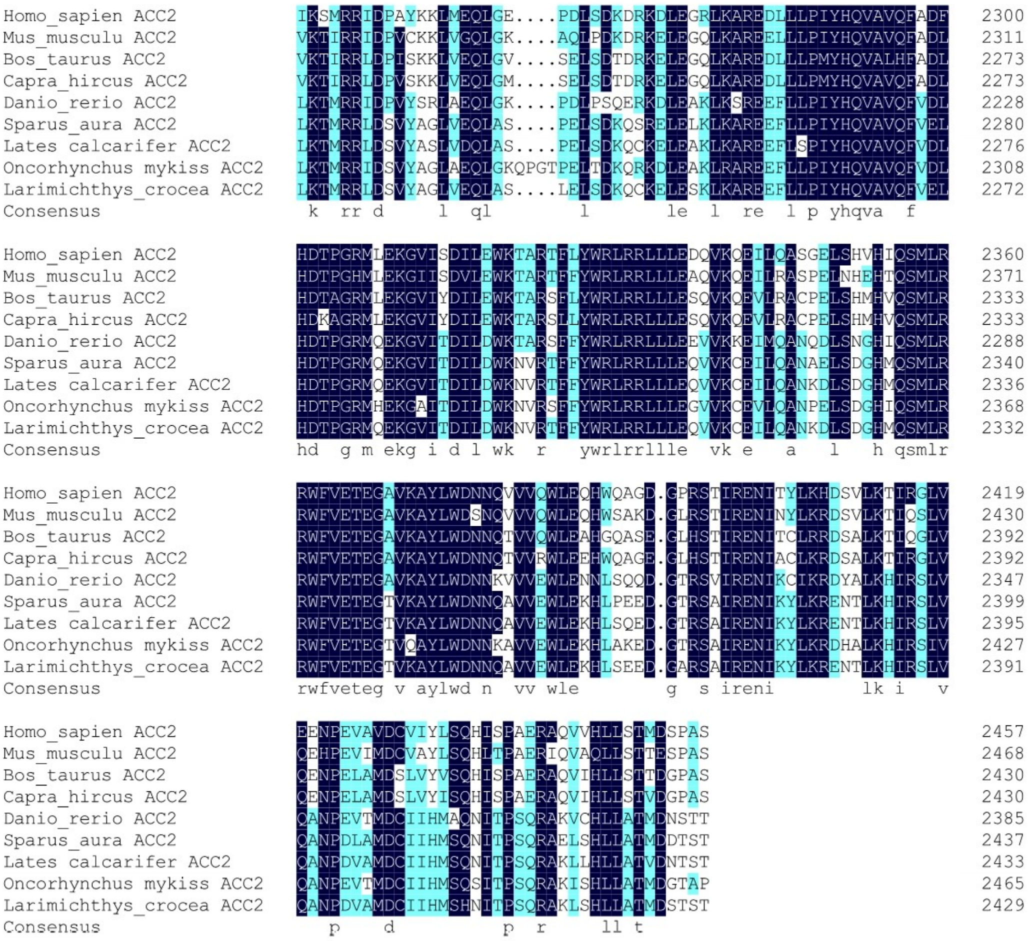

Figure S4 Multiple sequence alignment of the deduced AA sequences of large yellow croaker ACC2 with other fish species or mammals. Sequences comparison was performed using DNAMAN, and the identity/similarity shading is based on a 75% identity threshold. Identical residues are shown in dark blue shading and similar residues are shown in wathet blue shading. The identity shading is based on 81% identity threshold.
